# Supplementary material for: Role of cognitive ability in the association between functional health literacy and mortality in the Lothian Birth Cohort 1936: a prospective cohort study
Source: BMJ Open. 2018 Sep 10;8(9):e022502. doi: 10.1136/bmjopen-2018-022502 (PMC6144414; doi:10.1136/bmjopen-2018-022502)
Supplement: Supplementary data [file bmjopen-2018-022502supp001.pdf]

Supplementary material for: The role of cognitive ability in the association between functional health literacy and mortality in the Lothian Birth Cohort 1936: a prospective cohort study

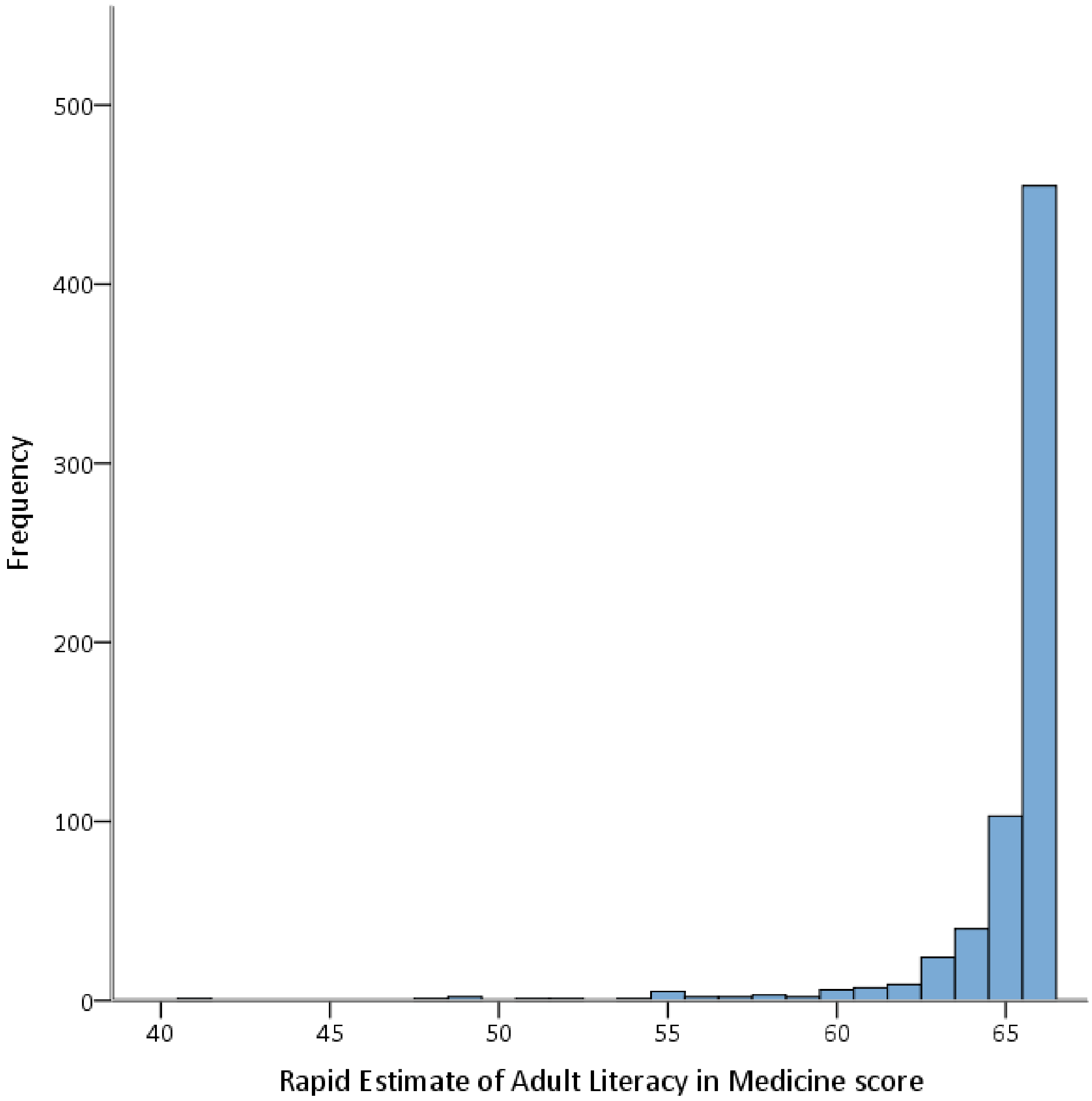

Supplementary Figure 1 Distribution of scores on the Rapid Estimate of Adult Literacy in Medicine for participants who were alive at censoring date

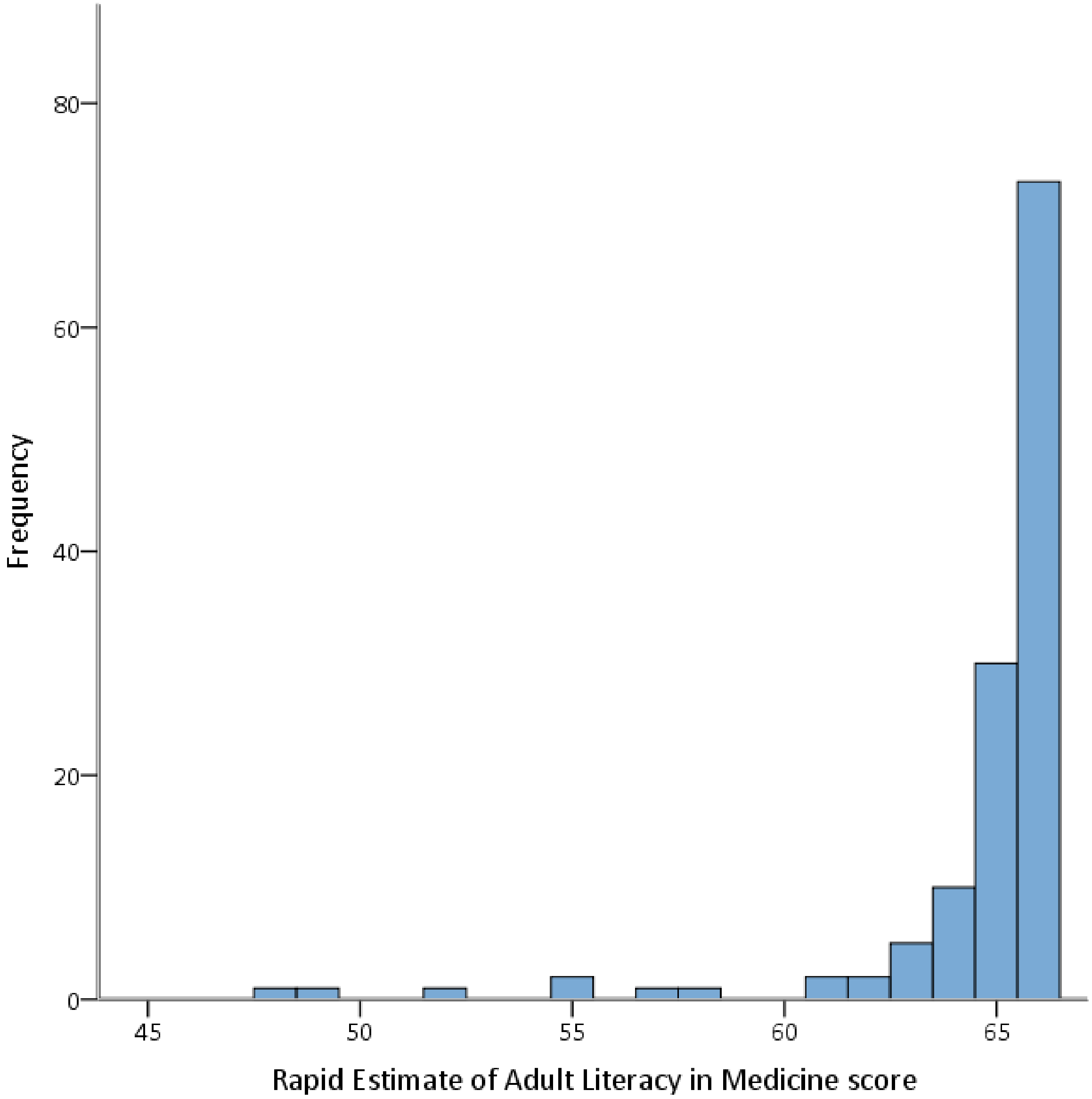

Supplementary Figure 2 Distribution of scores on the Rapid Estimate of Adult Literacy in Medicine for participants who had died by censoring date

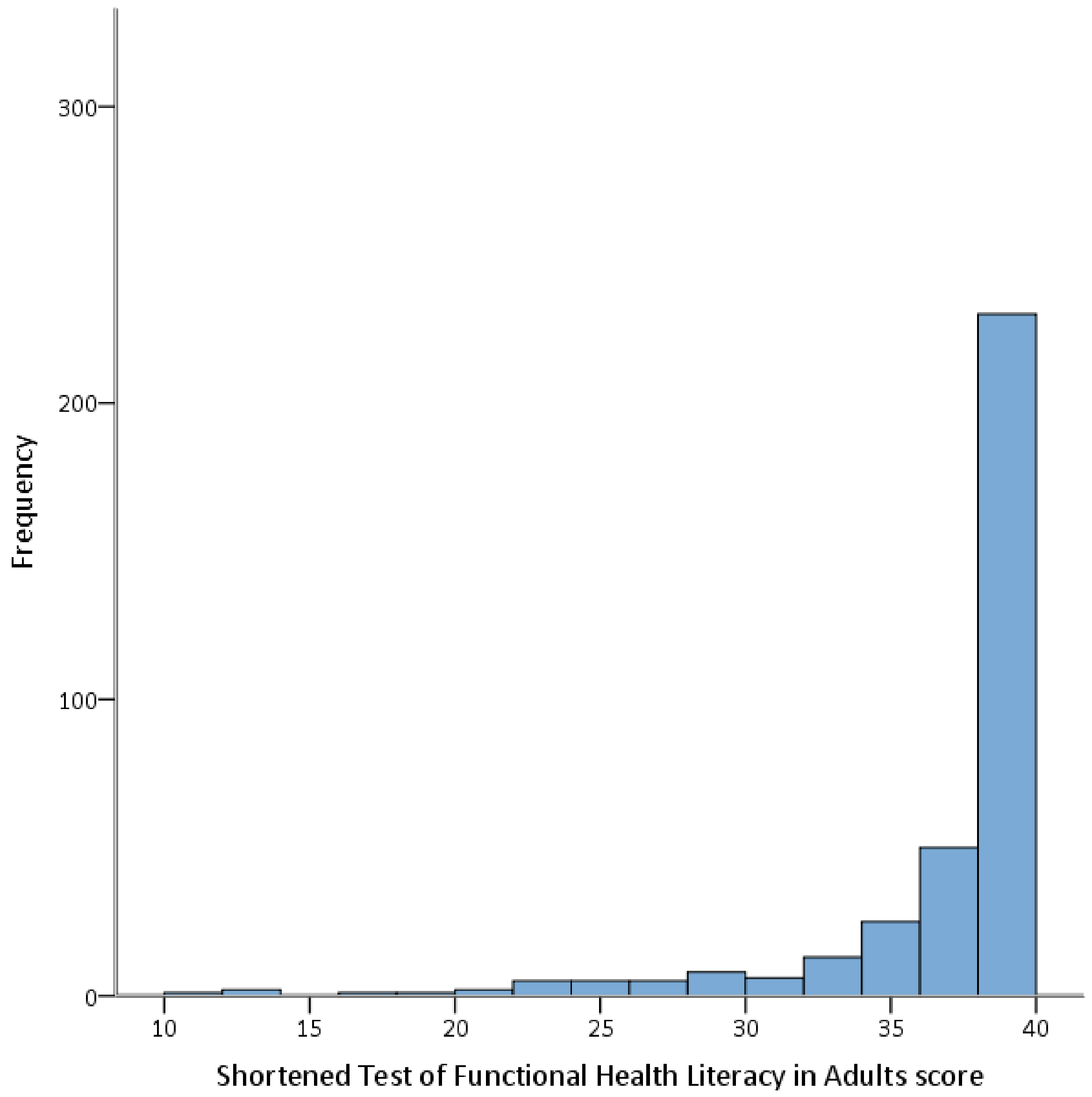

Supplementary Figure 3 Distribution of scores on the Shortened Test of Functional Health Literacy in Adults for participants who were alive at censoring date

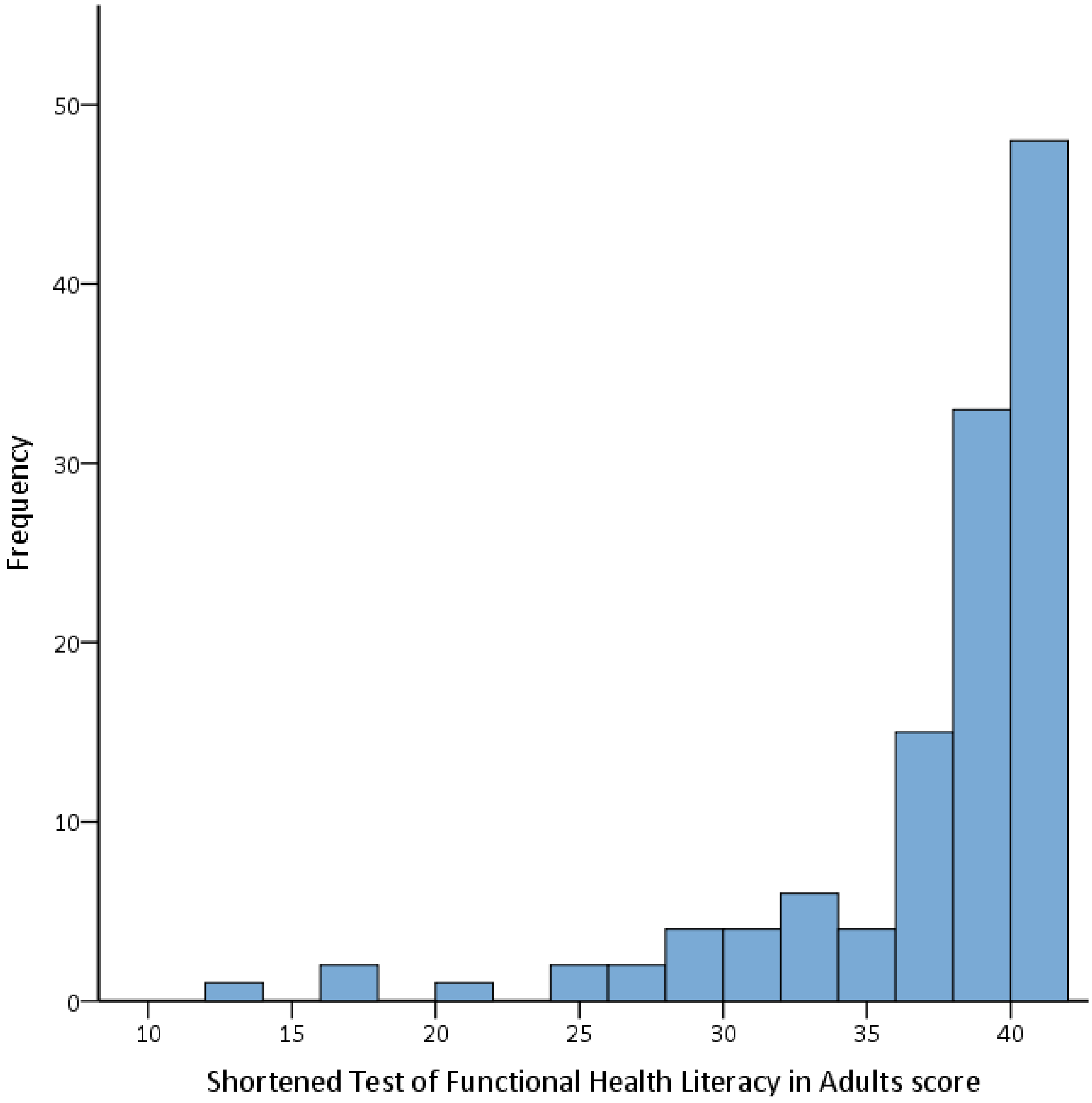

Supplementary Figure 4 Distribution of scores on the Shortened Test of Functional Health Literacy in Adults for participants who has died by censoring date

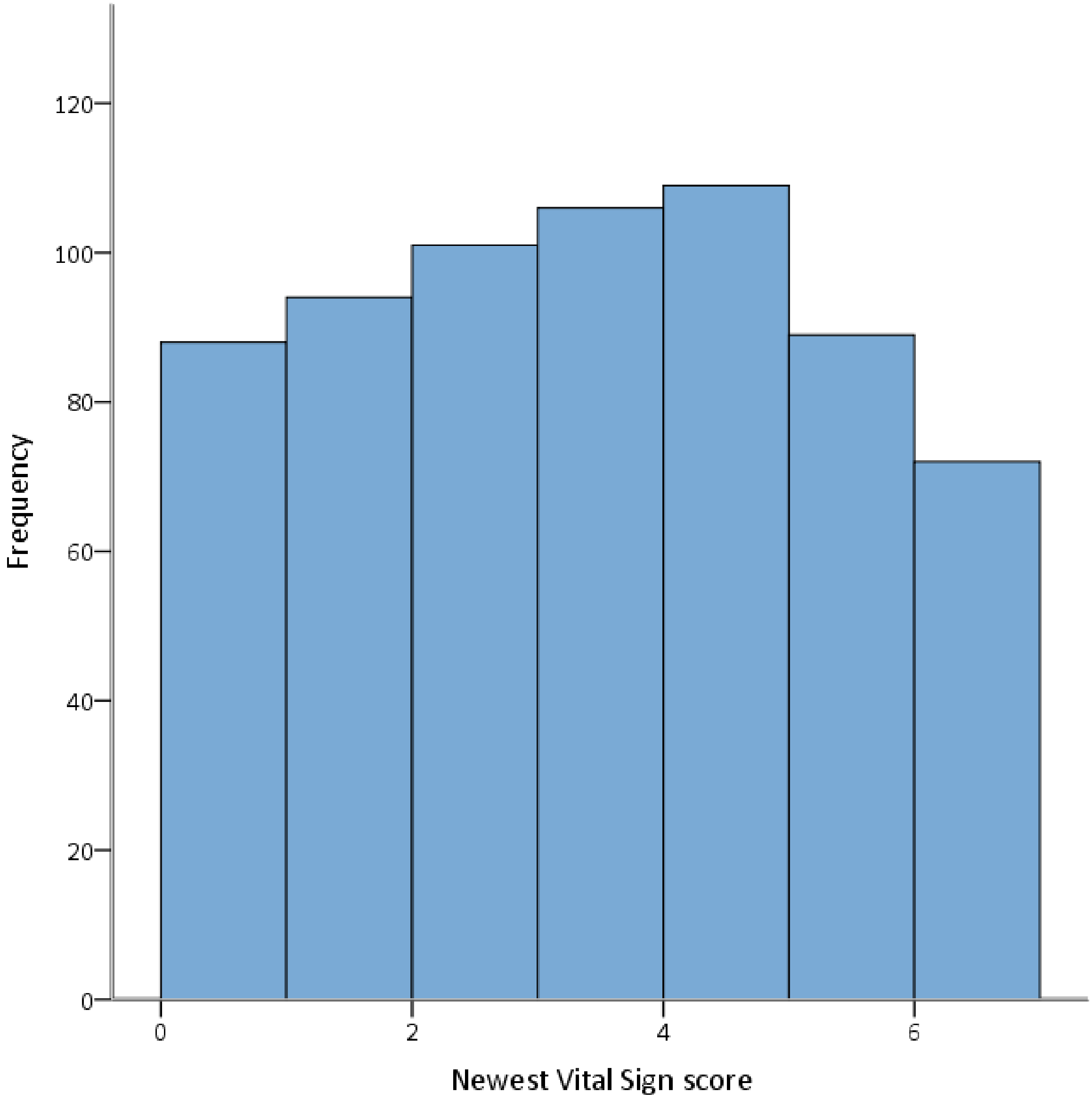

Supplementary Figure 5 Distribution of scores on the Newest Vital Sign for participants who were alive at censoring date

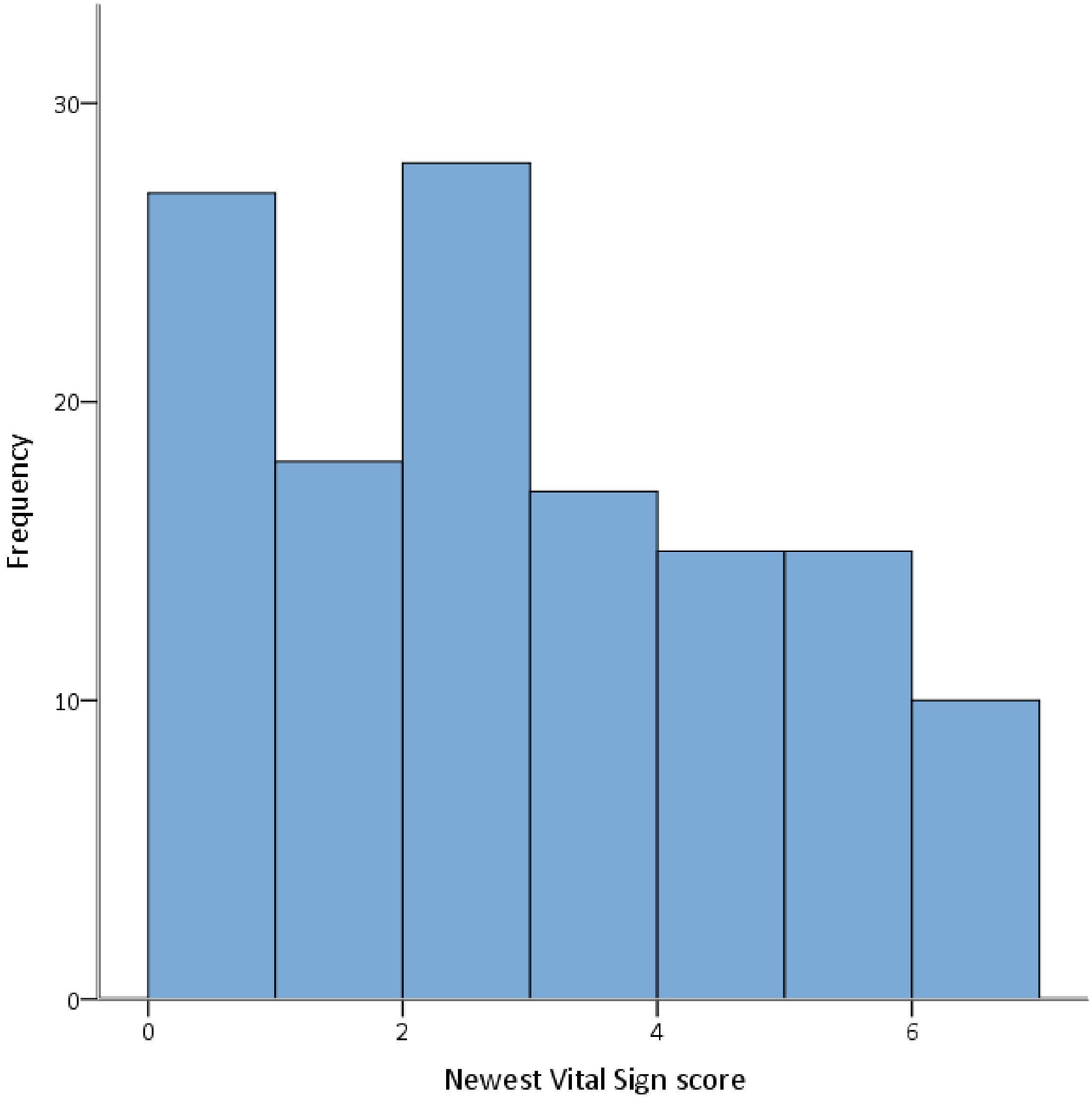

Supplementary Figure 6 Distribution of scores on the Newest Vital Sign for participants who had died by censoring date

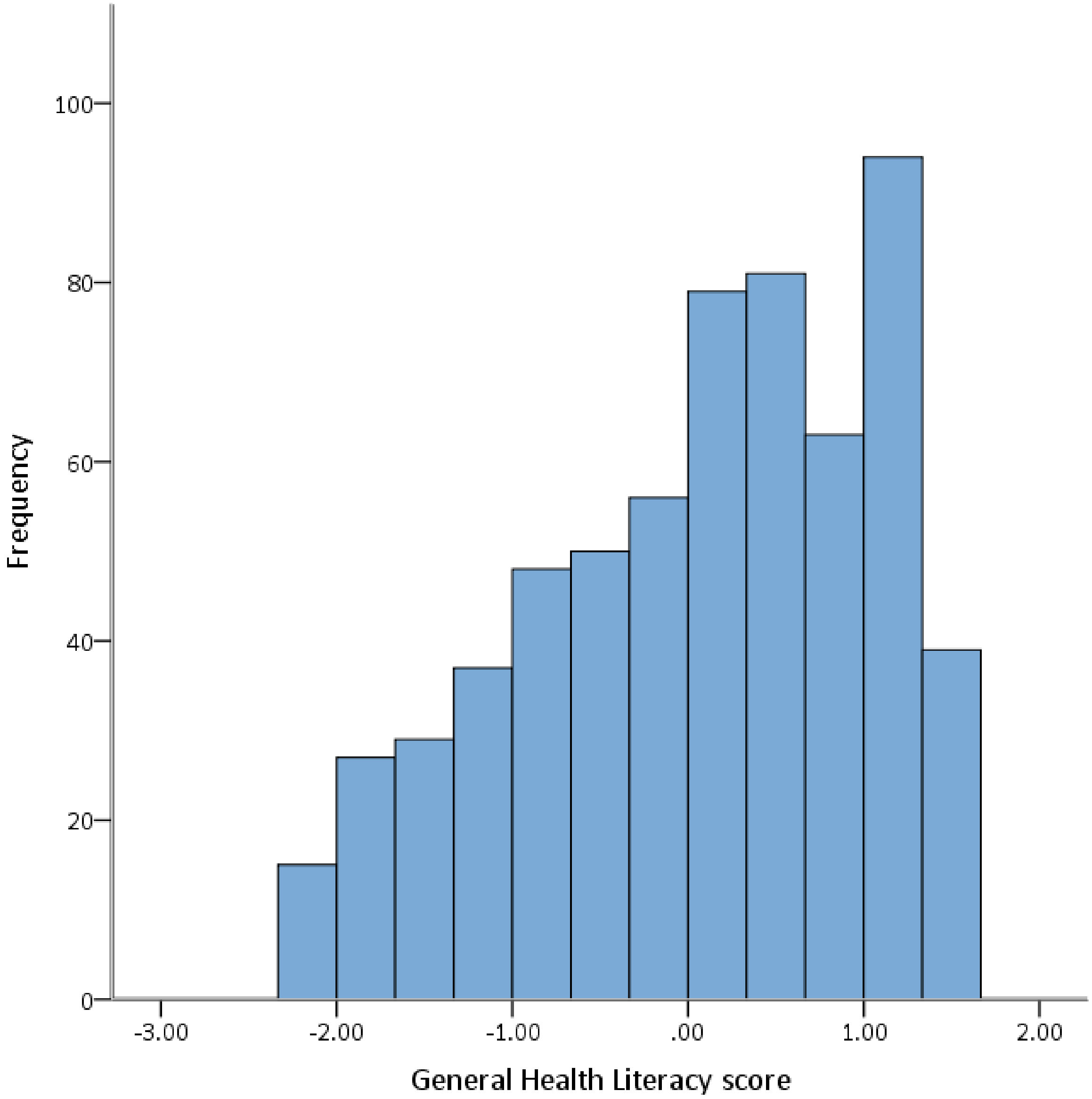

Supplementary Figure 7 Distribution of scores on General Health Literacy for participants who were alive at censoring date

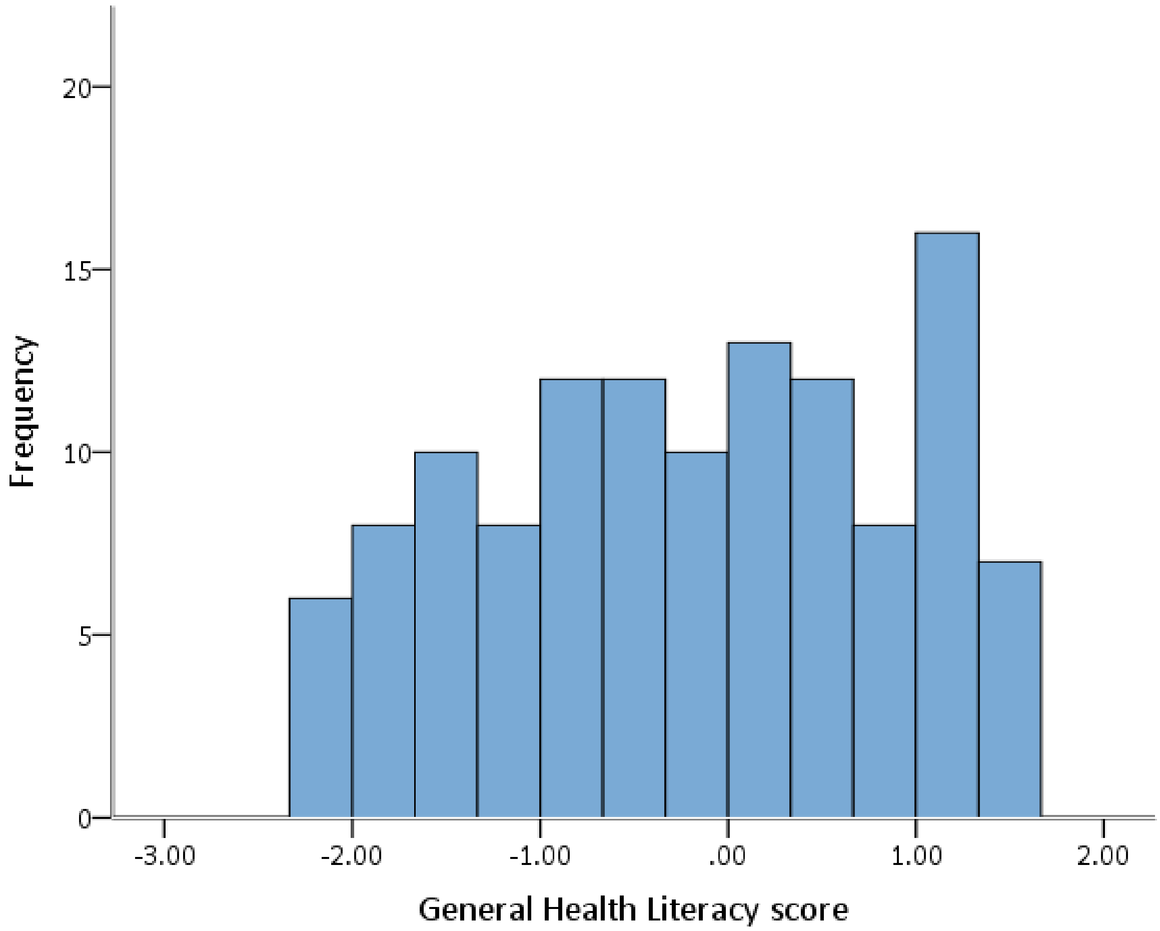

Supplementary Figure 8 Distribution of scores on General Health Literacy for participants who had died by censoring date

Supplementary Table 1 Rank order correlations between sociodemographic, functional health literacy, cognitive and health variables

	1	2	3	4	5	6	7	8	9	10	11	12	13
1 Age	-												
2 Sex†	0.04	-											
3 REALM	-0.12**	0.17***	-										
4 S-TOFHLA	-0.05	0.10**	0.40***	-									
5 NVS	-0.12**	0.01	0.35***	0.44***	-								
6 General functional health literacy	-0.09*	0.14***	0.71***	0.80***	0.78***	-							
7 Age-11 IQ	-0.07*	0.11**	0.44***	0.48***	0.51***	0.61***	-						
8 Fluid ability	-0.13***	0.00	0.38***	0.55***	0.55***	0.63***	0.57***	-					
9 Education	-0.05	0.03	0.31***	0.33***	0.37***	0.45***	0.45***	0.37***	-				
10 Occup class	0.05	-0.15***	-0.31***	-0.31***	-0.32***	-0.39***	-0.40***	-0.35***	-0.47***	-			
11 Self-rated health	-0.02	0.06	0.12**	0.20***	0.11**	0.18***	0.17***	0.24***	0.11**	-0.11**	-		
12 HADS	0.06	0.08*	-0.07	-0.13**	-0.11**	-0.14***	-0.13***	-0.22***	-0.08*	0.08*	-0.32***	-	
13 Townsend	0.13***	0.16***	-0.08*	-0.12**	-0.15***	-0.14***	-0.12**	-0.17***	-0.12**	0.09*	-0.35***	0.22***	-

**p* < .05, ***p* < .01, ****p* < .001.

†Correlations are point-biseral correlations. Female is coded 1 and male is coded 2.

Occupational class (ranging from 1-professional to 4-manual) and self-rated health (ranging from 1-poor/fair to 3-very good/excellent) are entered as ordinal variables.

REALM, Rapid Estimate of Adult Literacy in Medicine; S-TOFHLA, Shortened Test of Functional Health Literacy in Adults; NVS, Newest Vital Sign; IQ, Intelligence Quotient; Occup class, occupational class; HADS, Hospital Anxiety and Depression Scale; Townsend, Townsend Disability Scale.

DETAILED RESULTS

REALM: Hazard ratios (HRs) and 95% confidence intervals (CIs) for the associations between the REALM and mortality are shown in Supplementary Table 2. In Model 1, in which age and sex were controlled, the REALM did not significantly predict mortality (HR = 0.954, 95% CI 0.904 to 1.007), nor did age or sex. The REALM remained a non-significant predictor of mortality in Model 2, with the addition of years of education. Years of education did not predict mortality (HR = 0.963, 95% CI 0.822 to 1.128). Age-11 IQ was added in Model 3, and this did little to change the association between the REALM and mortality. Age-11 IQ did not predict mortality (HR = 0.993, 95% CI 0.980 to 1.006). The REALM remained a non-significant predictor of mortality following the inclusion of current fluid ability in Model 4. A one SD increase in fluid ability score reduced the risk of death by 37.9% (HR = 0.621, 95% CI 0.496 to 0.777). In Model 5, occupational social class was included in the model. The REALM remained non-significant. Individuals with a managerial/technical social class (HR = 2.278, 95% CI 1.161 to 4.470), a skilled non-manual social class (HR = 2.464, 95% CI 1.167 to 5.201) or a skilled manual social class (HR = 3.608, 95% CI 1.647 to 7.907) had a higher risk of death than individuals with a professional social class. Health status variables were additionally added in Model 6. The REALM remained a non-significant predictor of mortality. In this model, individuals with more years of education had a higher risk of dying (HR = 1.232, 95% CI 1.018 to 1.492). Risk of death for those who self-reported their health as fair or poor was over 2 times greater than those who reported their health to be very good or excellent (HR = 2.071, 95% CI 1.147 to 3.739). Whereas HADS score did not predict mortality, Townsend disability did. A one-point increase on the Townsend disability scale increased risk of mortality by 13.3% (HR = 1.133, 95% CI 1.044 to 1.229).

S-TOFHLA: The HRs for the association between S-TOFHLA and mortality are shown in Supplementary Table 3. In Model 1, controlling for age and sex, S-TOFHLA significantly predicted mortality. A one-point increase in S-TOFHLA reduced the risk of death by 5.2% (HR = 0.948, 95% CI 0.919 to 0.978). In this model, age and sex did not predict mortality. Adding years of education in Model 2 did not change the association between the S-TOFHLA and mortality. Years of education did

not predict mortality (HR = 1.020, 95% CI 0.870 to 1.197). The inclusion of age-11 IQ in Model 3 did not change the association between the S-TOFHLA and mortality. Age-11 IQ also did not predict mortality (HR = 0.997, 95% CI 0.983 to 1.011). The association between the S-TOFHLA and mortality was attenuated and became non-significant (HR = 0.967, 95% CI 0.929 to 1.007) in Model 4, additionally accounting current fluid ability. Current fluid ability significantly predicted mortality in this model. A one SD increase in fluid ability reduced the risk of death by 30.5% (HR = 0.695, 95% CI 0.545 to 0.887). Occupational class was included in Model 5, and the association between S-TOFHLA and mortality remained non-significant. Individuals with more years of education, controlling for other sociodemographic variables and cognitive function, had increased risk of death (HR = 1.219, 95% CI 1.004 to 1.481). Risk of dying was three times greater for participants with a skilled manual social class, compared to individuals with a professional social class (HR = 3.096, 95% CI 1.385 to 6.922). S-TOFHLA remained a non-significant predictor or mortality in Model 6, which included health status variables. Self-reporting health as fair or poor, compared to very good or excellent, was associated with increased risk of mortality (HR = 2.209, 95% CI 1.216 to 4.014). Higher scores on the HADS were not associated with mortality, while a higher Townsend disability score increased risk of death (HR = 1.131, 95% CI 1.039 to 1.232).

NVS: HRs for the association between NVS and mortality are shown in Supplementary Table 4. In Model 1, in which age and sex were entered as covariates, NVS significantly predicted mortality. A one point increase in NVS score reduced the risk of death by 11.8% (HR = 0.882, 95% CI 0.805 to 0.966). Age and sex did not predict mortality. Years of education was included in Model 2 and this did not change the association between the NVS and mortality. Years of education did not predict mortality (HR = 1.007, 95% CI 0.855 to 1.186). Age-11 IQ was additionally added to the model in Model 3 and this did little to change the association between NVS and mortality and this association remained significant. Age-11 IQ did not predict mortality (HR = 0.995, 95% CI 0.982 to 1.008). The inclusion of fluid ability in Model 4 greatly attenuated the association between NVS and mortality, and this became non-significant (HR = 0.963, 95% CI 0.860 to 1.078). Fluid ability was strongly associated with risk of death. A one SD increase in fluid ability score reduced risk of dying by 37.0%

(HR = 0.630, 95% CI 0.496 to 0.800). The association between NVS and mortality remained non-significant in Model 5 following inclusion of occupational class in the model. Compared to those with a professional social class, participants with managerial or technical (HR = 2.288, 95% CI 1.166 to 4.490), skilled non-manual (HR = 2.421, 95% CI 1.147 to 5.112), and skilled manual (HR = 3.631, 95% CI 1.658 to 7.951) social class had an increased risk of death. Finally, health status variables were included in Model 6. The inclusion of health status variables did little to change the association between NVS and mortality, which remained non-significant. In this model, having more years of education was associated with increased risk of mortality (HR = 1.242, 95% CI 1.023 to 1.508). Those who reported their health as fair or poor had 2.10 times (HR = 2.099, 95% CI 1.167 to 3.775) increased risk of mortality, compared to those who self-reported their health as very good or excellent. Participants with higher scores on the Townsend disability scale also had an increased risk of mortality (HR = 1.132, 95% CI 1.044 to 1.228).

General functional health literacy: HRs for the association between general functional health literacy and mortality are shown in Supplementary Table 5. General functional health literacy predicted mortality in Model 1 (HR = 0.774, 95% CI 0.650 to 0.922), while age and sex did not. A one point increase in the general functional health literacy score reduced the risk of mortality by 22.6%. Adding years of education (Model 2) did little to change the association between general functional health literacy and mortality and this association remained significant. Years of education was not associated with mortality (HR = 1.080, 95% CI 0.909 to 1.284). General functional health literacy remained a significant predictor of mortality when age-11 IQ was added in Model 3. Age-11 IQ did not predict morality (HR = 0.999, 95% CI 0.984 to 1.014). The inclusion of current fluid ability in Model 4 attenuated the association between general functional health literacy and risk of death, and this association became non-significant (HR = 0.871, 95% CI 0.674 to 1.125). Fluid ability was a significant predictor of mortality, such that a one SD increase in fluid ability reduced risk of death by 31.3% (HR = 0.687, 95% CI 0.531 to 0.887). Including occupational social class in Model 5 did little to change the association between general functional health literacy and mortality, and this association remained non-significant. In Model 5, individuals with more years of education had a greater risk of

death (HR = 1.240, 95% CI 1.019 to 1.508), and those with an occupational social class of skilled manual (HR = 3.134, 95% CI 1.405 to 6.991), when compared to those with a professional occupational class, had an increased risk of mortality. Finally, health status variables were added in Model 6. The association between general functional health literacy and mortality was attenuated further and remained non-significant. Reporting fair or poor health, compared to reporting very good or excellent health increased the risk of mortality (HR = 2.229, 95% CI 1.229 to 4.042). Higher Townsend disability scores were also associated with increased risk of death (HR = 1.128, 95% CI 1.040 to 1.225). In this final model, controlling for sociodemographics and health variables, as well as age-11 IQ, the association between fluid ability and mortality was attenuated and became non-significant (HR = 0.770, 95% CI 0.589 to 1.007).

Supplementary Table 2 Hazard ratios (95% confidence intervals) for the association between REALM and mortality, controlling for sociodemographic, cognitive ability, and health status variables

	Model 1<br>Age and sex<br>N = 794	Model 2<br>+ education<br>N = 794	Model 3<br>+ age-11 IQ<br>N = 752	Model 4<br>+ current fluid ability<br>N = 746	Model 5<br>+ occup class<br>N = 731	Model 6<br>+ health status<br>N = 728
REALM	0.954 (0.904 to 1.007)	0.957 (0.905 to 1.013)	0.962 (0.903 to 1.025)	0.971 (0.907 to 1.039)	0.970 (0.904 to 1.040)	0.996 (0.924 to 1.074)
Age	0.940 (0.725 to 1.219)	0.939 (0.724 to 1.218)	0.944 (0.725 to 1.231)	0.879 (0.669 to 1.154)	0.908 (0.686 to 1.203)	0.933 (0.704 to 1.235)
Sex						
Female	Reference	Reference	Reference	Reference	Reference	Reference
Male	1.297 (0.909 to 1.850)	1.298 (0.910 to 1.852)	1.252 (0.869 to 1.802)	1.333 (0.927 to 1.918)	1.176 (0.787 to 1.756)	1.364 (0.898 to 2.073)
Years of education		0.963 (0.822 to 1.128)	1.022 (0.862 to 1.211)	1.089 (0.916 to 1.295)	1.201 (0.995 to 1.450)	1.232 (1.018 to 1.492)*
Age-11 IQ			0.993 (0.980 to 1.006)	1.008 (0.993 to 1.023)	1.009 (0.994 to 1.024)	1.008 (0.993 to 1.024)
Fluid ability				0.621 (0.496 to 0.777)***	0.662 (0.526 to 0.834)***	0.727 (0.574 to 0.922)**
Occupational class						
Professional					Reference	Reference
Managerial/technical					2.278 (1.161 to 4.470)*	2.218 (1.127 to 4.365)*
Skilled: non-manual					2.464 (1.167 to 5.201)*	2.596 (1.232 to 5.474)*
Skilled: manual					3.608 (1.647 to 7.907)**	3.393 (1.532 to 7.516)**
Partly skilled/ unskilled manual					2.054 (0.651 to 6.473)	2.067 (0.656 to 6.510)
Self-rated health						
Very good/excellent						Reference
Good						1.153 (0.742 to 1.791)
Fair/poor						2.071 (1.147 to 3.739)*
HADS total score						0.972 (0.929 to 1.018)
Townsend disability						1.133 (1.044 to 1.229)**

* $p < .05$, ** $p < .01$, *** $p < .001$.

REALM, Rapid Estimate of Adult Literacy in Medicine; IQ, Intelligence Quotient; occup class, Occupational class; HADS, Hospital Anxiety and Depression Scale.

Supplementary Table 3 Hazard ratios (95% confidence intervals) for the association between S-TOFHLA and mortality, controlling for sociodemographic, cognitive ability, and health variables

	Model 1<br>Age and sex<br>N = 744	Model 2<br>+ education<br>N = 744	Model 3<br>+ age-11 IQ<br>N = 702	Model 4<br>+ current fluid ability<br>N = 697	Model 5<br>+ occup class<br>N = 682	Model 6<br>+ health status<br>N = 680
S-TOFHLA	0.948 (0.919 to 0.978)**	0.947 (0.917 to 0.978)**	0.947 (0.913 to 0.982)**	0.967 (0.929 to 1.007)	0.976 (0.935 to 1.019)	0.998 (0.953 to 1.046)
Age	0.882 (0.665 to 1.170)	0.882 (0.665 to 1.170)	0.889 (0.666 to 1.186)	0.871 (0.652 to 1.164)	0.919 (0.682 to 1.238)	0.936 (0.697 to 1.256)
Sex						
Female	Reference	Reference	Reference	Reference	Reference	Reference
Male	1.307 (0.909 to 1.879)	1.309 (0.910 to 1.881)	1.277 (0.881 to 1.851)	1.349 (0.930 to 1.956)	1.204 (0.797 to 1.818)	1.352 (0.881 to 2.074)
Years of education		1.020 (0.870 to 1.197)	1.066 (0.896 to 1.268)	1.111 (0.932 to 1.326)	1.219 (1.004 to 1.481)*	1.249 (1.026 to 1.520)*
Age-11 IQ			0.997 (0.983 to 1.011)	1.006 (0.991 to 1.022)	1.007 (0.991 to 1.022)	1.006 (0.991 to 1.022)
Fluid ability				0.695 (0.545 to 0.887)**	0.717 (0.557 to 0.922)*	0.759 (0.587 to 0.982)*
Occupational class						
Professional					Reference	Reference
Managerial/technical					1.889 (0.956 to 3.734)	1.844 (0.931 to 3.650)
Skilled: non-manual					2.108 (0.994 to 4.470)	2.207 (1.042 to 4.673)*
Skilled: manual					3.096 (1.385 to 6.922)**	2.881 (1.275 to 6.509)*
Partly skilled/ unskilled manual					1.786 (0.566 to 5.636)	1.773 (0.562 to 5.598)
Self-rated health						
Very good/excellent						Reference
Good						1.147 (0.728 to 1.807)
Fair/poor						2.209 (1.216 to 4.014)**
HADS total score						0.974 (0.930 to 1.021)
Townsend disability						1.131 (1.039 to 1.232)**

$*p < .05$, $**p < .01$, $***p < .001$.

S-TOFHLA, Shortened Test of Functional Health Literacy in Adults; IQ, Intelligence Quotient; occup class, Occupational class; HADS, Hospital Anxiety and Depression Scale.

Supplementary Table 4 Hazard ratios (95% confidence intervals) for the association between NVS and mortality, controlling for sociodemographic, cognitive ability, and health variables

	Model 1<br>Age and sex<br>N = 789	Model 2<br>+ education<br>N = 789	Model 3<br>+ age-11 IQ<br>N = 746	Model 4<br>+ current fluid ability<br>N = 742	Model 5<br>+ occup class<br>N = 727	Model 6<br>+ health status<br>N = 724
NVS	0.882 (0.805 to 0.966)**	0.880 (0.799 to 0.970)*	0.892 (0.802 to 0.992)*	0.963 (0.860 to 1.078)	0.967 (0.861 to 1.086)	0.961 (0.853 to 1.082)
Age	0.942 (0.727 to 1.221)	0.942 (0.726 to 1.221)	0.942 (0.722 to 1.228)	0.890 (0.678 to 1.168)	0.919 (0.694 to 1.217)	0.937 (0.708 to 1.242)
Sex						
Female	Reference	Reference	Reference	Reference	Reference	Reference
Male	1.343 (0.946 to 1.906)	1.343 (0.947 to 1.907)	1.279 (0.892 to 1.834)	1.346 (0.939 to 1.928)	1.180 (0.791 to 1.760)	1.355 (0.893 to 2.057)
Years of education		1.007 (0.855 to 1.186)	1.056 (0.888 to 1.257)	1.093 (0.917 to 1.302)	1.208 (0.998 to 1.463)	1.242 (1.023 to 1.508)*
Age-11 IQ			0.995 (0.982 to 1.008)	1.007 (0.993 to 1.021)	1.008 (0.993 to 1.023)	1.009 (0.994 to 1.023)
Fluid ability				0.630 (0.496 to 0.800)***	0.670 (0.524 to 0.857)**	0.748 (0.580 to 0.966)*
Occupational class						
Professional					Reference	Reference
Managerial/technical					2.288 (1.166 to 4.490)*	2.243 (1.140 to 4.414)*
Skilled: non-manual					2.421 (1.147 to 5.112)*	2.593 (1.231 to 5.463)*
Skilled: manual					3.631 (1.658 to 7.951)**	3.360 (1.522 to 7.415) **
Partly skilled/<br>unskilled manual					2.125 (0.677 to 6.669)	2.086 (0.661 to 6.578)
Self-rated health						
Very good/excellent						Reference
Good						1.175 (0.756 to 1.826)
Fair/poor						2.099 (1.167 to 3.775)*
HADS total score						0.973 (0.930 to 1.018)
Townsend disability						1.132 (1.044 to 1.228)**

*p < .05, **p < .01.

NVS, Newest Vital Sign; IQ, Intelligence Quotient; occup class, Occupational class; HADS, Hospital Anxiety and Depression Scale.

Supplementary Table 5 Hazard ratios (95% confidence intervals) for the association between general functional health literacy and mortality, controlling for sociodemographic, cognitive ability, and health variables

	Model 1<br>Age and sex<br>N = 740	Model 2<br>+ education<br>N = 740	Model 3<br>+ age-11 IQ<br>N = 698	Model 4<br>+ current fluid ability<br>N = 694	Model 5<br>+ occup class<br>N = 679	Model 6<br>+ health status<br>N = 677
General functional health literacy	0.774 (0.650 to 0.922)**	0.746 (0.615 to 0.905)**	0.738 (0.585 to 0.931)*	0.871 (0.674 to 1.125)	0.911 (0.700 to 1.186)	0.950 (0.725 to 1.245)
Age	0.897 (0.678 to 1.187)	0.893 (0.675 to 1.182)	0.902 (0.677 to 1.200)	0.885 (0.663 to 1.182)	0.933 (0.693 to 1.257)	0.942 (0.700 to 1.266)
Sex						
Female	Reference	Reference	Reference	Reference	Reference	Reference
Male	1.276 (0.886 to 1.838)	1.272 (0.883 to 1.833)	1.238 (0.852 to 1.799)	1.327 (0.912 to 1.930)	1.178 (0.778 to 1.784)	1.337 (0.869 to 2.056)
Years of education		1.080 (0.909 to 1.284)	1.119 (0.936 to 1.339)	1.134 (0.948 to 1.357)	1.240 (1.019 to 1.508)*	1.255 (1.030 to 1.528)*
Age-11 IQ			0.999 (0.984 to 1.014)	1.006 (0.991 to 1.022)	1.006 (0.991 to 1.022)	1.007 (0.992 to 1.023)
Fluid ability				0.687 (0.531 to 0.887)**	0.707 (0.543 to 0.921)*	0.770 (0.589 to 1.007)
Occupational class						
Professional					Reference	Reference
Managerial/technical					1.901 (0.962 to 3.756)	1.870 (0.945 to 3.700)
Skilled: non-manual					2.076 (0.979 to 4.401)	2.192 (1.035 to 4.640)*
Skilled: manual					3.134 (1.405 to 6.991)**	2.823 (1.252 to 6.365)*
Partly skilled/ unskilled manual					1.824 (0.580 to 5.741)	1.759 (0.557 to 5.561)
Self-rated health						
Very good/excellent						Reference
Good						1.152 (0.733 to 1.810)
Fair/poor						2.229 (1.229 to 4.042)**
HADS total score						0.975 (0.931 to 1.022)
Townsend disability						1.128 (1.040 to 1.225)**

* $p < .05$, **$p < .01$.

General functional health literacy, general measure of functional health literacy created by entering the REALM, S-TOFHLA and NVS into a PCA; IQ, Intelligence Quotient; occup class, Occupational class; HADS, Hospital Anxiety and Depression Scale.

Supplementary Table 6 Hazard ratios (95% confidence intervals) for the association between REALM and mortality, controlling for sociodemographic, cognitive ability, and health status variables. Models are run on a sub-sample participants with all variables of interest (N = 728).

	Model 1<br>Age and sex	Model 2<br>+ education	Model 3<br>+ age-11 IQ	Model 4<br>+ current fluid ability	Model 5<br>+ occup class	Model 6<br>+ health status
REALM	0.944 (0.894 to 0.997)*	0.946 (0.894 to 1.001)	0.959 (0.900 to 1.021)	0.966 (0.904 to 1.033)	0.969 (0.904 to 1.039)	0.996 (0.924 to 1.074)
Age	1.002 (0.763 to 1.316)	1.001 (0.762 to 1.315)	0.999 (0.761 to 1.312)	0.931 (0.704 to 1.231)	0.930 (0.700 to 1.234)	0.933 (0.704 to 1.235)
Sex						
Female	Reference	Reference	Reference	Reference	Reference	Reference
Male	1.303 (0.897 to 1.892)	1.304 (0.898 to 1.893)	1.289 (0.887 to 1.872)	1.358 (0.935 to 1.971)	1.224 (0.815 to 1.836)	1.364 (0.898 to 2.073)
Years of education		0.981 (0.831 to 1.158)	1.010 (0.848 to 1.204)	1.077 (0.902 to 1.287)	1.203 (0.994 to 1.455)	1.232 (1.018 to 1.492)*
Age-11 IQ			0.993 (0.980 to 1.006)	1.007 (0.992 to 1.023)	1.009 (0.993 to 1.025)	1.008 (0.993 to 1.024)
Fluid ability				0.632 (0.503 to 0.794)***	0.666 (0.528 to 0.841)**	0.727 (0.574 to 0.922)**
Occupational class						
Professional					Reference	Reference
Managerial/technical					2.201 (1.118 to 4.333)*	2.218 (1.127 to 4.365)*
Skilled: non-manual					2.482 (1.175 to 5.245)*	2.596 (1.232 to 5.474)*
Skilled: manual					3.570 (1.627 to 7.837)**	3.393 (1.532 to 7.516)**
Partly skilled/ unskilled manual					2.023 (0.641 to 6.388)	2.067 (0.656 to 6.510)
Self-rated health						
Very good/excellent						Reference
Good						1.153 (0.742 to 1.791)
Fair/poor						2.071 (1.147 to 3.739)*
HADS total score						0.972 (0.929 to 1.018)
Townsend disability						1.133 (1.044 to 1.229)**

*$p < .05$, **$p < .01$, ***$p < .001$.

REALM, Rapid Estimate of Adult Literacy in Medicine; IQ, Intelligence Quotient; occup class, Occupational class; HADS, Hospital Anxiety and Depression Scale.

Supplementary Table 7 Hazard ratios (95% confidence intervals) for the association between S-TOFHLA and mortality, controlling for sociodemographic, cognitive ability, and health variables. Models are run on a subsample of participants with all variables of interest (N = 680).

	Model 1<br>Age and sex	Model 2<br>+ education	Model 3<br>+ age-11 IQ	Model 4<br>+ current fluid ability	Model 5<br>+ occup class	Model 6<br>+ health status
S-TOFHLA	0.947 (0.917 to 0.978)**	0.945 (0.914 to 0.977)**	0.949 (0.913 to 0.985)**	0.969 (0.930 to 1.010)	0.975 (0.934 to 1.018)	0.998 (0.953 to 1.046)
Age	0.924 (0.688 to 1.242)	0.925 (0.688 to 1.242)	0.927 (0.690 to 1.245)	0.911 (0.677 to 1.224)	0.919 (0.681 to 1.240)	0.936 (0.697 to 1.256)
Sex						
Female	Reference	Reference	Reference	Reference	Reference	Reference
Male	1.304 (0.893 to 1.902)	1.306 (0.895 to 1.905)	1.298 (0.889 to 1.896)	1.356 (0.928 to 1.981)	1.233 (0.814 to 1.866)	1.352 (0.881 to 2.074)
Years of education		1.033 (0.874 to 1.222)	1.046 (0.875 to 1.250)	1.092 (0.911 to 1.309)	1.208 (0.994 to 1.469)	1.249 (1.026 to 1.520)*
Age-11 IQ			0.997 (0.983 to 1.011)	1.006 (0.991 to 1.022)	1.007 (0.992 to 1.023)	1.006 (0.991 to 1.022)
Fluid ability				0.699 (0.545 to 0.895)**	0.717 (0.556 to 0.923)*	0.759 (0.587 to 0.982)*
Occupational class						
Professional					Reference	Reference
Managerial/technical					1.853 (0.935 to 3.670)	1.844 (0.931 to 3.650)
Skilled: non-manual					2.105 (0.992 to 4.464)	2.207 (1.042 to 4.673)*
Skilled: manual					3.038 (1.358 to 6.796)**	2.881 (1.275 to 6.509)*
Partly skilled/ unskilled manual					1.755 (0.556 to 5.541)	1.773 (0.562 to 5.598)
Self-rated health						
Very good/excellent						Reference
Good						1.147 (0.728 to 1.807)
Fair/poor						2.209 (1.216 to 4.014)**
HADS total score						0.974 (0.930 to 1.021)
Townsend disability						1.131 (1.039 to 1.232)**

*$p < .05$, **$p < .01$.

S-TOFHLA, Shortened Test of Functional Health Literacy in Adults; IQ, Intelligence Quotient; occup class, Occupational class; HADS, Hospital Anxiety and Depression Scale.

Supplementary Table 8 Hazard ratios (95% confidence intervals) for the association between NVS and mortality, controlling for sociodemographic, cognitive ability, and health variables. Models are run on a sub-sample of participants with all variables of interest (N = 724).

	Model 1<br>Age and sex	Model 2<br>+ education	Model 3<br>+ age-11 IQ	Model 4<br>+ current fluid ability	Model 5<br>+ occup class	Model 6<br>+ health status
NVS	0.880 (0.800 to 0.968)**	0.875 (0.790 to 0.968)*	0.887 (0.796 to 0.989)*	0.953 (0.850 to 1.070)	0.960 (0.854 to 1.079)	0.961 (0.853 to 1.082)
Age	0.993 (0.756 to 1.306)	0.993 (0.756 to 1.306)	0.992 (0.754 to 1.304)	0.944 (0.714 to 1.248)	0.940 (0.709 to 1.248)	0.937 (0.708 to 1.242)
Sex						
Female	Reference	Reference	Reference	Reference	Reference	Reference
Male	1.346 (0.933 to 1.943)	1.346 (0.933 to 1.943)	1.326 (0.916 to 1.919)	1.373 (0.950 to 1.986)	1.228 (0.820 to 1.840)	1.355 (0.893 to 2.057)
Years of education		1.029 (0.866 to 1.222)	1.048 (0.876 to 1.253)	1.084 (0.905 to 1.298)	1.212 (0.999 to 1.470)	1.242 (1.023 to 1.508)*
Age-11 IQ			0.995 (0.982 to 1.008)	1.006 (0.992 to 1.021)	1.008 (0.993 to 1.023)	1.009 (0.994 to 1.023)
Fluid ability				0.645 (0.506 to 0.822)***	0.678 (0.529 to 0.869)**	0.748 (0.580 to 0.966)*
Occupational class						
Professional					Reference	Reference
Managerial/technical					2.211 (1.123 to 4.354)*	2.243 (1.140 to 4.414)*
Skilled: non-manual					2.435 (1.152 to 5.146)*	2.593 (1.231 to 5.463)*
Skilled: manual					3.590 (1.637 to 7.874)**	3.360 (1.522 to 7.415) **
Partly skilled/<br>unskilled manual					2.101 (0.668 to 6.604)	2.086 (0.661 to 6.578)
Self-rated health						
Very good/excellent						Reference
Good						1.175 (0.756 to 1.826)
Fair/poor						2.099 (1.167 to 3.775)*
HADS total score						0.973 (0.930 to 1.018)
Townsend disability						1.132 (1.044 to 1.228)**

*p < .05, **p < .01.

NVS, Newest Vital Sign; IQ, Intelligence Quotient; occup class, Occupational class; HADS, Hospital Anxiety and Depression Scale.

Supplementary Table 9 Hazard ratios (95% confidence intervals) for the association between general functional health literacy and mortality, controlling for sociodemographic, cognitive ability, and health variables. Models are run on a sub-sample of participants with all variables of interest (N = 677).

	Model 1<br>Age and sex	Model 2<br>+ education	Model 3<br>+ age-11 IQ	Model 4<br>+ current fluid ability	Model 5<br>+ occup class	Model 6<br>+ health status
General health literacy	0.769 (0.640 to 0.924)**	0.736 (0.602 to 0.901)**	0.742 (0.586 to 0.939)*	0.868 (0.669 to 1.126)	0.903 (0.694 to 1.176)	0.950 (0.725 to 1.245)
Age	0.940 (0.701 to 1.260)	0.937 (0.699 to 1.256)	0.937 (0.699 to 1.257)	0.925 (0.688 to 1.243)	0.934 (0.692 to 1.260)	0.942 (0.700 to 1.266)
Sex						
Female	Reference	Reference	Reference	Reference	Reference	Reference
Male	1.264 (0.863 to 1.851)	1.256 (0.858 to 1.840)	1.255 (0.857 to 1.839)	1.328 (0.906 to 1.947)	1.205 (0.794 to 1.829)	1.337 (0.869 to 2.056)
Years of Education		1.096 (0.915 to 1.312)	1.098 (0.914 to 1.320)	1.114 (0.927 to 1.340)	1.229 (1.010 to 1.497)*	1.255 (1.030 to 1.528)*
Age-11 IQ			0.999 (0.984 to 1.014)	1.006 (0.991 to 1.022)	1.007 (0.991 to 1.023)	1.007 (0.992 to 1.023)
Fluid ability				0.692 (0.534 to 0.898)**	0.708 (0.543 to 0.922)*	0.770 (0.589 to 1.007)
Occupational class						
Professional					Reference	Reference
Managerial/technical					1.863 (0.941 to 3.689)	1.870 (0.945 to 3.700)
Skilled: non-manual					2.070 (0.976 to 4.390)	2.192 (1.035 to 4.640)*
Skilled: manual					3.072 (1.377 to 6.857)**	2.823 (1.252 to 6.365)*
Partly skilled/ unskilled manual					1.794 (0.570 to 5.649)	1.759 (0.557 to 5.561)
Self-rated health						
Very good/excellent						Reference
Good						1.152 (0.733 to 1.810)
Fair/poor						2.229 (1.229 to 4.042)**
HADS total score						0.975 (0.931 to 1.022)
Townsend disability						1.128 (1.040 to 1.225)**

*$p < .05$, **$p < .01$.

General health literacy, general measure of health literacy created by entering the REALM, S-TOFHLA and NVS into a PCA; IQ, Intelligence Quotient; occup class, Occupational class; HADS, Hospital Anxiety and Depression Scale.
