# Supplementary material for: Role of cognitive ability in the association between functional health literacy and mortality in the Lothian Birth Cohort 1936: a prospective cohort study
Source: BMJ Open. 2018 Sep 10;8(9):e022502. doi: 10.1136/bmjopen-2018-022502 (PMC6144414; doi:10.1136/bmjopen-2018-022502)
Supplement: Author's manuscript [file bmjopen-2018-022502.draft_revisions.pdf]

BMJ Open

BMJ Open is committed to open peer review. As part of this commitment we make the peer review history of every article we publish publicly available.

When an article is published we post the peer reviewers' comments and the authors' responses online. We also post the versions of the paper that were used during peer review. These are the versions that the peer review comments apply to.

The versions of the paper that follow are the versions that were submitted during the peer review process. They are not the versions of record or the final published versions. They should not be cited or distributed as the published version of this manuscript.

BMJ Open is an open access journal and the full, final, typeset and author-corrected version of record of the manuscript is available on our site with no access controls, subscription charges or pay-per-view fees (http://bmjopen.bmj.com).

If you have any questions on BMJ Open's open peer review process please email info.bmjopen@bmj.com

BMJ Open

The role of cognitive ability in the association between health literacy and mortality in the Lothian Birth Cohort 1936: a prospective cohort study

Journal:	*BMJ Open*
Manuscript ID	bmjopen-2018-022502
Article Type:	Research
Date Submitted by the Author:	22-Feb-2018

Complete List of Authors:	Fawns-Ritchie, Chloe; University of Edinburgh, Centre for Cognitive Ageing and Cognitive Epidemiology<br>Starr, John; University of Edinburgh<br>Deary, Ian; Dept of Psychology

Keywords:	EPIDEMIOLOGY, PUBLIC HEALTH, STATISTICS & RESEARCH METHODS

SCHOLARONE™
Manuscripts

For peer review only

The role of cognitive ability in the association between health literacy and mortality in the Lothian Birth Cohort 1936: a prospective cohort study

Chloe Fawns-Ritchie[1,2], John M Starr[1,3], and Ian J Deary[1,2*]

[1]Centre for Cognitive Ageing and Cognitive Epidemiology, University of Edinburgh, Edinburgh, UK

[2]Department of Psychology, University of Edinburgh, Edinburgh, UK

[3]Alzheimer Scotland Dementia Research Centre, University of Edinburgh, Edinburgh, UK

*Corresponding author: Ian J Deary, Centre for Cognitive Ageing and Cognitive Epidemiology, Department of Psychology, University of Edinburgh

7 George Square

Edinburgh, EH8 9JZ

Scotland, UK

Telephone: +44 131 650 3452

i.deary@ed.ac.uk

Word count: 4011

For peer review only - http://bmjopen.bmj.com/site/about/guidelines.xhtml

ABSTRACT

Objectives We investigated the role that childhood and old age cognitive ability play in the association between health literacy and mortality.

Design Prospective cohort study.

Setting This study used data from the Lothian Birth Cohort 1936 study, which recruited participants living in the Lothian region of Scotland when aged 70 years, most of whom had completed an intelligence test at age 11.

Participants 795 members of the Lothian Birth Cohort 1936 with scores on measures of health literacy and cognitive ability in childhood and older adulthood.

Primary and secondary outcome measures Participants were followed up for 8 years to determine mortality. Time to death in days was used as the primary outcome measure.

Results Using Cox regression, higher health literacy was associated with lower risk of mortality adjusting for age and sex, using the Shortened Test of Functional Health Literacy in Adults (HR = 0.948, 95% CI 0.919 to 0.978), the Newest Vital Sign (HR = 0.882, 95% CI 0.805 to 0.966), and a general health literacy measure (HR = 0.774, 95% CI 0.650 to 0.922), but not the Rapid Estimate of Adult Literacy in Medicine (HR = 0.954, 95% CI 0.904 to 1.007). Adjusting for childhood intelligence did not change these associations. When additionally adjusting for fluid-type cognitive ability in older age associations between health literacy and mortality were attenuated and non-significant.

Conclusions Current fluid ability but not childhood intelligence attenuated the association between health literacy and mortality. Health literacy measures may, in part, assess fluid-type cognitive abilities and this may account for the association between health literacy and mortality.

For peer review only - http://bmjopen.bmj.com/site/about/guidelines.xhtml

Strengths and limitations of this study

- This study had multiple tests of health literacy which measure different components of health literacy.
- This study had comprehensive measures of cognitive ability measured in both childhood and old age which allowed us to investigate whether childhood and old age cognitive ability independently played a role in the relationship between health literacy and mortality.
- Larger samples and a longer follow-up time are needed to determine the role of cognitive ability in the association between health literacy and cause-specific mortality.

For peer review only

For peer review only - http://bmjopen.bmj.com/site/about/guidelines.xhtml

INTRODUCTION

Health literacy is "the degree to which individuals have the capacity to obtain, process and understand basic health information and services needed to make basic health decisions".[1] This ability is thought to be multifaceted and encompass the set of skills required to navigate the health care environment, including reading, numeracy, and knowledge relating to health.[2, 3] Health literacy is thought to be important at all levels of health care, including making decisions about seeking and following medical advice, self-management of chronic illnesses, and undertaking health-promoting behaviours. These activities require individuals to find, understand, and act upon health information.[4]

Tests have been developed to measure these skills, including the commonly used Test of Functional Health Literacy in Adults.[5] Performance on health literacy tests have been associated with a range of health outcomes. Individuals with lower health literacy are more likely to require emergency care and have poorer skills in relation to correctly taking medication and interpreting written health materials.[6] Individuals with higher health literacy are more likely to take part in health-promoting behaviours such as eating a healthy diet, and are more likely to take part in routine cancer screening.[7, 8]

Successful completion of health literacy measures rely on cognitive functions, such as processing capacity and reasoning.[2, 3] One dominant theory in intelligence research is that there is a distinction between fluid ability, the ability to problem solve using novel material, which tends to decline with increasing age, and crystallised ability, which is the knowledge acquired throughout life which remains relatively stable across the lifespan.[9-13] Completing tests of health literacy requires both crystallised abilities such as specific knowledge relating to health, and fluid abilities such as reasoning.[2, 3] Performance on tests of health literacy and cognitive ability are strongly related.[14-21] Some tests of health

For peer review only - http://bmjopen.bmj.com/site/about/guidelines.xhtml

literacy have been found to correlate more strongly with measures of cognitive ability than with each other.[20, 22, 23] This overlap is so strong that some have proposed that health literacy should not be considered a unique construct but, instead, should be thought of as a specific component of cognitive function.[23]

Researchers have investigated the role that cognitive function plays in the association between health literacy and health status. Whereas most evidence suggests that cognitive function explains a large proportion of the association between health literacy and health, the degree of attenuation varies.[22, 24, 25] A study using participants from the Lothian Birth Cohort 1936[22]—the same sample used in the current study—investigated whether cognitive ability in childhood and late adulthood attenuated the association between health literacy and measures of physical health. In models without cognitive measures, health literacy was associated with all three of the measures of physical health assessed. Addition of cognitive ability in older age significantly attenuated the association between health literacy with physical fitness and number of natural teeth; however, it did not attenuate the association between health literacy and body mass index (BMI). Conversely, while childhood cognitive ability did not attenuate the association between health literacy and physical fitness, it attenuated the association between health literacy and number of teeth by 30%, and BMI by 88%. In the fully adjusted model which included childhood and late adulthood cognitive ability, as well as other early-life factors, the association between health literacy and physical fitness, though attenuated, remained significant.[22]

Mortality is arguably one of the most important health outcomes to examine. Both cognitive ability[26, 27] and health literacy[28] have been found to predict mortality. Researchers have therefore investigated the degree to which cognitive function explains the association between health literacy and mortality. In two studies,[29, 30] cognitive function was found to attenuate the association between health literacy and risk of dying; however, the association

For peer review only - http://bmjopen.bmj.com/site/about/guidelines.xhtml

between health literacy and mortality remained, thus, cognitive function did not fully explain this relationship. These two studies, however, used brief measures of health literacy and cognitive function.

The current analysis sought to better understand the relationship between health literacy, cognitive ability and mortality using comprehensive measures of health literacy and cognitive ability. This study used the LBC1936—the same sample as used in Mõttus et al.[22]—which has a wealth of cognitive data, including measures of childhood and old age cognitive ability. The aim was to understand the role that both the trait of lifelong intelligence, measured using an intelligence test administered at age 11 years, and current cognitive ability in older age, measured at approximately 73 years and contemporaneously with health literacy, play in any association between health literacy and mortality.

METHODS

Participants

LBC1936 is a cohort study of 1091 older adults born in 1936, most of whom reside in the Lothian area in Scotland. Most had taken part in the Scottish Mental Survey 1947, which tested the intelligence of almost all children born in 1936 and attending Scottish schools on 4th June 1947.[31] LBC1936 consists of a sample of these individuals who were subsequently followed-up, for the first time, at age 70 years (wave 1). To date, these participants have been followed-up a further three times at approximately 3 year intervals (waves 2-4). LBC1936 was designed to investigate healthy cognitive ageing. Detailed information on this cohort is provided elsewhere.[32, 33] The present study used a sub-sample of 795 (413 male, 382 female) LBC1936 participants who completed tests of health literacy at wave 2 when participants were approximately aged 73 years. Ethical approval was obtained from the

For peer review only - http://bmjopen.bmj.com/site/about/guidelines.xhtml

Scotland A Research Ethics Committee (07/MRE00/58). This study conformed to the principles embodied in the Declaration of Helsinki.

Measures

Mortality and survival time

The General Register Office for Scotland was used to identify deaths. Deaths through to end of March 2017 were recorded and this date is used as the censoring date for participants who survived. Survival time was measured in days from date of attending study visit at wave 2 to date of death or censoring date.

Health literacy

Health literacy tests were administered at wave 2.

Rapid Estimate of Adult Literacy in Medicine (REALM):[34] This test measures participants' ability to read and correctly pronounce medical words. Participants are presented a piece of paper with a list of 66 medical words and are asked to read these words aloud. The words range in difficulty from easy ("fat") to difficult ("impetigo"). One point is given for each correctly pronounced word. One week test-retest (r = 0.99)[34] and internal consistency (Cronbach's alpha = 0.98)[35] have been found to be very high.

Shortened Test of Functional Health Literacy in Adults (S-TOFHLA):[5, 36] In the numeracy section, participants are provided with cards with medical information on them and are asked four questions about this information. The reading comprehension section comprised a 36-item task which involved participants reading two health-related passages where every fifth to seventh word was missing and participants were to select the missing

For peer review only - http://bmjopen.bmj.com/site/about/guidelines.xhtml

word from four options. Here, the British version of the S-TOFHLA[8] was used which substitutes the Medicaid passage for a passage about UK prescription fee exemptions. This measure is seen as the gold standard health literacy test[37] and successful completion of the S-TOFHLA requires the ability to read and comprehend written words and numbers in a health context. Internal consistency is high for reading comprehension (Cronbach's alpha = 0.97)[36] and adequate for numeracy (Cronbach's alpha = 0.68).[36] The S-TOFHLA has been found to correlate strongly with the REALM (r = 0.80).[36]

Newest Vital Sign (NVS):[38] Participants were presented with a nutrition label from a container of ice cream and were asked to answer six questions about the information provided on this label. The NVS assesses both reading and numeracy skills associated with health as participants need to use the written text and numbers on the label to answer the questions.[38] The NVS correlates with the S-TOFHLA at r = 0.59[38] and shows reasonable internal consistency (Cronbach's alpha = 0.76).[38]

General health literacy: Health literacy is multifaceted and the measures described above individually only measure some aspects of health literacy. A general measure of health literacy was created which is thought to more accurately represent the complex nature of this ability by entering scores on the three health literacy tests into a principal component analysis (PCA). Two of these measures had skewed distributions (see Supplementary Figures 1-8), therefore Spearman's rank correlation was used in the PCA. Only the first component had an eigenvalue greater than 1, and the scree slope indicated a single component; therefore, scores from the first unrotated principal component were used as a measure of health literacy (general health literacy). This component accounted for 59.7% of the total variance, and the REALM, S-TOFHLA and NVS loaded 0.74, 0.80, and 0.77, respectively, on this component.

For peer review only - http://bmjopen.bmj.com/site/about/guidelines.xhtml

Cognitive ability

Childhood cognitive ability (age-11 IQ): As part of the Scottish Mental Survey 1947, almost all 11 year old children in Scotland in 1947 sat the Moray House Test No. 12 (MHT);[31] a 45-minute, group-administered intelligence test that included tasks of verbal reasoning and spatial ability, and had a maximum score of 76. In LBC1936, scores on the MHT were adjusted for age in days at testing and then were converted into standard IQ-type scores with a mean of 100 and a standard deviation of 15. This score will be used as a measure of prior, or crystallised, ability.

Current fluid ability: Participants completed a lengthy cognitive assessment.[32, 33] As has been done in previous LBC1936 studies,[20, 22] six tests administered at wave 2 thought to measure fluid-type cognitive abilities that tend decline across the lifespan[11-13] were entered into a PCA. The following tests from the Wechsler Adult Intelligence Scale-III[39] that assess non-verbal reasoning, visuospatial ability, working memory, and processing speed were used: Matrix Reasoning, Block Design, Letter-Number Sequencing, Symbol Search, Digit Span Backwards, and Digit Symbol-coding. Only the first component had an eigenvalue greater than 1 and the scree slope indicated one component, and therefore scores from this first principal component were used as a measure of current fluid ability. This component accounted 50.2% of the total variance. The loadings for the six tests were: Matrix Reasoning = 0.69; Block Design = 0.71; Letter-Number Sequencing = 0.71; Symbol Search = 0.75; Digit Span Backwards = 0.64; Digit Symbol-coding = 0.75.

Covariates

Sociodemographic variables included in this analysis were education and occupational social class. Years of full-time education completed, recorded at wave 1 when participants were

For peer review only - http://bmjopen.bmj.com/site/about/guidelines.xhtml

aged 70 years, was used to measure education. At wave 1, participants were assigned to one of the following occupational social classes based on their highest occupational status prior to retirement:[40] professional, managerial and technical, skilled, partly skilled manual, unskilled manual. Female participants were assigned the occupational class of their husband if this was higher than their own. Skilled was separated into skilled non-manual and skilled manual. Only 5 participants in this sample were assigned the occupational class of unskilled, therefore partly skilled manual, and unskilled manual were combined into one class, hereafter referred to as manual (N = 31).

Three measures of health status measured at wave 2 were used. Self-reported health was measured by asking participants whether they rated their general health to be excellent, very good, good, fair or poor. Only a small number of participants who were recorded dead at the censoring date reported poor (N = 3) or excellent (N = 17) health. Therefore, poor and fair were collapsed into one category (fair/poor; N = 73), as were very good and excellent (very good/excellent; N = 487). Total score on the Hospital Anxiety and Depression Scale (HADS)[41] was used as a measure of mood state. Higher scores on the HADS represent higher levels of anxiety and depression. Activities of daily living were assessed using the Townsend Disability Scale.[42] Participants were given a score of 0 (no difficulty completing this activity) to 2 (not able complete this activity) for nine activities, and thus higher scores represent more functional disability.

Patient and public involvement

LBC1936 participants were not involved in the development of any part of this study. The results will be disseminated to participants via a quarterly newsletter sent to LBC1936 participants.

For peer review only - http://bmjopen.bmj.com/site/about/guidelines.xhtml

Statistical analysis

SPSS version 21.0 was used to carry out this analysis. To determine whether those recorded as alive or dead at censoring date differ on demographic, health literacy, cognitive function, or health status variables, chi-square tests were conducted for categorical variables, independent t-tests were used for normally-distributed continuous variables, and Mann-Whitney U tests were used for non-normal continuous variables. Spearman's rank-order correlation was used to examine the relationship between health literacy and cognitive ability scores. To investigate the association between health literacy and time to death, Cox proportional hazard regression was used. For each of the health literacy measures of interest (REALM, S-TOFHLA, NVS, and the component score of general health literacy) five models were run. In Model 1 the health literacy measure of interest and age and sex were entered. To determine whether cognitive ability in childhood attenuated the association between health literacy and mortality, age-11 IQ was added (Model 2). In Model 3, fluid-type cognitive ability in older age was additionally added to determine its role in the association between health literacy and mortality. Years of education and occupational class were additionally included in Model 4. Health status variables (self-reported health, HADS, and Townsend) were included in Model 5. Methods to control for multiple testing were not used here. We were interested in the change in the effect size of the association between health literacy and mortality following the inclusion of various cognitive, sociodemographic and health variables. In the results section of the main text here, only the hazard ratios (HRs) and 95% confidence intervals (CIs) for health literacy are reported. A more detailed description of the results for all variables in the models is given in the supplementary materials.

For peer review only - http://bmjopen.bmj.com/site/about/guidelines.xhtml

RESULTS

A total of 796 participants completed the health literacy measures at wave 2. Following removal of one participant without information on date of death, 130 participants had died, and 665 participants were alive at the censoring date. Participant characteristics are reported in Table 1 and health literacy and cognitive ability scores are shown in Table 2. Those who died were more likely to be from a lower occupational class, were more likely to report poorer health, and reported more disability than those who survived. Participants who had died had lower scores on all the health literacy measures, and had lower fluid cognitive ability scores in older age. Age-11 IQ did not differ between the two groups.

Table 3 shows the rank order correlations between health literacy and cognitive ability measures. These have been reported elsewhere.[20, 22] The three health literacy measures correlated moderately with each other (r = 0.348-0.444, $p < .001$), and higher scores on the health literacy measures were correlated with higher age-11 IQ (r = 0.438-0.513, $p < .001$), and higher fluid ability (r = 0.378-0.550, $p < .001$). The three health literacy measures tended to correlate more strongly with measures of cognitive ability than with each other. The general health literacy measure also showed a strong positive correlation with both age-11 IQ (r = 0.611, $p < .001$) and fluid ability in older age (r = 0.632, $p < .001$). The correlations between all variables examined in this analysis are reported in Supplementary Table 1.

The HRs for the association between health literacy and mortality are shown in Table 4. HRs for all variables entered into the models are reported in Supplementary Tables 2-5. In all models, the assumptions of proportional hazards were met. Given the high correlations between health literacy and cognitive ability, variance inflation factors (VIF) were calculated to check for multicollinearity. VIF values for all models were low (highest VIF = 2.149), suggesting there was no multicollinearity in these models.

For peer review only - http://bmjopen.bmj.com/site/about/guidelines.xhtml

Table 1 Participant characteristics for participants alive or dead at censoring date and *p*-values to determine whether these characteristics differed by survival status

	N	Alive	Dead	*p*-value
Survival time (years), mean (SD)	795	8.19 (0.66)	5.23 (2.14)	
Age (years) at wave 2, mean (SD)	795	72.54 (0.70)	72.41 (0.72)	.068
Sex, n (%)	795			.069
Male		336 (50.5)	77 (59.2)	
Female		329 (49.5)	53 (40.8)	
Years of education, mean (SD)	795	10.80 (1.16)	10.71 (1.10)	.417
Occupational class, n (%)	780			.001
Professional		142 (21.7)	12 (9.4)	
Managerial/technical		249 (38.1)	49 (38.6)	
Skilled: non-manual		140 (21.4)	26 (20.5)	
Skilled: manual		96 (14.7)	35 (27.6)	
Manual		26 (4.0)	5 (3.9)	
Self-reported health, n (%)	795			<.001
Poor/fair		47 (7.1)	26 (19.9)	
Good		195 (29.4)	40 (30.5)	
Very good/excellent		422 (63.5)	65 (49.6)	
HADS total, mean (SD)	794	7.02 (4.37)	7.42 (4.62)	.342
Townsend disability, mean (SD)	794	0.89 (1.82)	1.60 (2.48)	.001

HADS, Hospital Anxiety and Depression Scale.

For peer review only

For peer review only - http://bmjopen.bmj.com/site/about/guidelines.xhtml

Table 2 Mean scores (SD) on measures of health literacy and cognitive ability by survival status, and *p*-values to determine whether these scores differ by survival status

	N	Alive	Dead	*p*-value
REALM score	794	65.08 (2.39)	64.67 (3.02)	.015
TOFHLA score	744	38.00 (3.85)	36.69 (5.37)	.025
NVS score	789	2.92 (1.90)	2.46 (1.91)	.011
General health literacy	740	0.05 (0.98)	-0.24 (1.08)	.007
Age-11 IQ	752	101.08 (14.99)	98.55 (16.33)	.091
Current fluid ability	789	0.07 (0.99)	-0.34 (1.00)	<.001

REALM, Rapid Estimate of Adult Literacy in Medicine; S-TOFHLA, Shortened Test of Functional Health Literacy in Adults; NVS, Newest Vital Sign; IQ, Intelligence Quotient.

For peer review only

REALM: The HRs for the REALM represent the risk of dying for a one point increase in the REALM (max score = 66). The REALM did not significantly predict mortality in Model 1 (HR = 0.954, 95% CI 0.904 to 1.007) adjusting for age and sex, or subsequently with the addition of age-11 IQ (Model 2), fluid ability (Model 3), sociodemographic (Model 4), or health status (Model 5).

S-TOFHLA: The HRs for the S-TOFHLA represent the risk of mortality for a one point increase in S-TOFHLA score (max score = 40). With age and sex controlled for, a one-point increase in S-TOFHLA reduced the risk of dying by 5.2% (Model 1 HR = 0.948, 95% CI 0.919 to 0.978). Inclusion of age-11 IQ (Model 2) did not attenuate this association. This association was attenuated and became non-significant in Model 3 with the inclusion of fluid ability (HR = 0.967, 95% CI 0.929 to 1.007), and remained non-significant and continued to

For peer review only - http://bmjopen.bmj.com/site/about/guidelines.xhtml

reduce in size following the inclusion of sociodemographic variables (Model 4) and health status in (Model 5).

NVS: The HRs for NVS represent the risk of mortality for a one point increase in NVS score (max score = 6). In Model 1, in which age and sex were entered as covariates, NVS significantly predicted mortality. A one point increase in NVS score reduced the risk of dying by 11.8% (HR = 0.882, 95% CI 0.805 to 0.966). Age-11 IQ was added in Model 2 and this did little to change the association between NVS and mortality. The inclusion of fluid ability in Model 3 greatly attenuated the association between NVS and mortality, and this association became non-significant (HR = 0.972, 95% CI 0.869 to 1.087). This association remained non-significant following the inclusion of sociodemographic variables (Model 4) and health status variables (Model 5).

General health literacy: The HRs for general health literacy represent the risk of mortality for a one SD increase in general health literacy. General health literacy predicted mortality in Model 1, controlling for age and sex. A one SD increase in general health literacy reduced the risk of mortality by 22.6% (HR = 0.774, 95% CI 0.650 to 0.922). Including age-11 IQ in Model 2 did little to change the association between general health literacy and mortality. Current fluid ability was included in Model 3 and this attenuated the association between general health literacy and mortality and this association was no longer significant (HR = 0.901, 95% CI 0.701 to 1.158). Adding years of education and occupational social class in Model 4 did little to change the association between general health literacy and mortality. Health status variables were added in Model 5 and the association between general health literacy and mortality was further attenuated and remained non-significant.

All models were re-run using only participants who had complete data on all of the variables of interest. These models are shown in Supplementary Tables 6-9. The associations between

For peer review only - http://bmjopen.bmj.com/site/about/guidelines.xhtml

health literacy and mortality were similar to those reported here, except that, in Model 1 for the REALM (Supplementary Table 6), higher scores on the REALM significantly reduced the risk of mortality. This association was no longer significant in Model 2, following the inclusion of age-11 IQ.

Table 3 Rank order correlations between health literacy and cognitive ability measures

	1	2	3	4	5	6
1 REALM	1.000					
2 S-TOFHLA	0.398*	1.000				
3 NVS	0.348*	0.444*	1.000			
4 General health literacy	0.706*	0.803*	0.781*	1.000		
5 Age-11 IQ	0.438*	0.481*	0.513*	0.611*	1.000	
6 Current fluid ability	0.378*	0.550*	0.549*	0.632*	0.565*	1.000

*p < .001.

REALM, Rapid Estimate of Adult Literacy in Medicine; S-TOFHLA, Shortened Test of Functional Health Literacy in Adults; NVS, Newest Vital Sign; IQ, Intelligence Quotient.

For peer review only - http://bmjopen.bmj.com/site/about/guidelines.xhtml

Table 4 Hazard ratios (95% confidence intervals) for the association between four measures of health literacy and mortality, controlling for sociodemographic, cognitive, and health variables

	Model 1<br>Age and sex	Model 2<br>+ age-11 IQ	Model 3<br>+ current fluid ability<br>in older age	Model 4<br>+ sociodemographics	Model 5<br>+ health status
REALM	0.954 (0.904 to 1.007)<br>N = 794	0.962 (0.903 to 1.025)<br>N = 752	0.972 (0.909 to 1.040)<br>N = 746	0.970 (0.904 to 1.040)<br>N = 731	0.996 (0.924 to 1.074)<br>N = 728
S-TOFHLA	0.948 (0.919 to 0.978)**<br>N = 744	0.948 (0.913 to 0.983)**<br>N = 702	0.967 (0.929 to 1.007)<br>N = 697	0.976 (0.935 to 1.019)<br>N = 682	0.998 (0.953 to 1.046)<br>N = 680
NVS	0.882 (0.805 to 0.966)**<br>N = 789	0.899 (0.810 to 0.997)*<br>N = 746	0.972 (0.869 to 1.087)<br>N = 742	0.967 (0.861 to 1.086)<br>N = 727	0.961 (0.853 to 1.082)<br>N = 724
General health literacy	0.774 (0.650 to 0.922)**<br>N = 740	0.766 (0.612 to 0.959)*<br>N = 698	0.901 (0.701 to 1.158)<br>N = 694	0.911 (0.700 to 1.186)<br>N = 679	0.950 (0.725 to 1.245)<br>N = 677

*$p < .05$, **$p < .01$

IQ, intelligence quotient; REALM, Rapid Estimate of Adult Literacy in Medicine; S-TOFHLA, Shortened Test of Functional Health Literacy in Adults; NVS, Newest Vital Sign.

For peer review only - http://bmjopen.bmj.com/site/about/guidelines.xhtml

For peer review only

DISCUSSION

This study investigated whether prior cognitive ability measured in childhood and current fluid cognitive ability measured in older adulthood played different roles in the association between health literacy and mortality. When age and sex were controlled for, S-TOFHLA, NVS and general health literacy predicted mortality; the REALM did not (though it had a slightly stronger and significant association when only those with full data were included, as shown in supplementary analysis). Individuals who had higher scores on the S-TOFHLA, NVS, and general health literacy had a lower risk of mortality than those with lower scores. Accounting for prior intelligence measured in childhood did not change this association. The association between health literacy and mortality disappeared when contemporaneous fluid ability was accounted for. The attenuation was particularly large for NVS and general health literacy.

Childhood cognitive ability, which was measured decades prior to the health literacy assessment, is thought to reflect the relatively stable trait of lifelong intelligence, whereas current fluid ability, which was measured when participants were approximately 73 years old, is a measure of current cognitive competence.[20] These results suggest that, whereas childhood intelligence did not play a role in the association between health literacy and mortality, current fluid-type cognitive ability in older adulthood accounted for a large proportion of this association.

Previous studies found that, although the size of the association between health literacy and mortality was reduced, health literacy still predicted mortality when cognitive function was controlled for.[29, 30] Here, fluid ability attenuated the association between health literacy and mortality such that the association was no longer significant. A strength of this current study is that detailed measures of cognitive ability were used. Childhood intelligence was

For peer review only - http://bmjopen.bmj.com/site/about/guidelines.xhtml

measured using a standardised test of intelligence which had good concurrent validity with other intelligence tests.[32] The fluid ability measure comprised many standardised neuropsychological tests. Both Baker et al.[29] and Bostock and Steptoe[30] used brief measures of cognitive function. Baker et al.[29] used specific items from the mini-mental state exam, a measure designed to screen for cognitive impairment[43] which is insensitive to individual differences in healthy cognitive ageing. Bostock and Steptoe[30] used three brief cognitive tests administered in a non-standardised way in the participants' own home. These studies may not have used tests sensitive enough, or that covered a necessary range of cognitive functions, to fully account for the association between health literacy and mortality.

The results of this study support the proposal by Reeve and Basalik[23] that health literacy may not be a unique construct, and instead, measures of health literacy are in fact domain-specific measures of cognitive ability. Here, NVS, S-TOFHLA and general health literacy no longer predicted mortality when accounting for fluid ability; however, this attenuation was not seen for childhood cognitive ability. This suggests that these tests are likely to be substantially measuring more fluid-type cognitive abilities that decline with increasing age.[12]

We note that, because of the heterogeneity of the health literacy tests, a general health literacy measure may be a problematic method of assessing health literacy. This general measure was derived with the aim of creating a score that more accurately reflects the multifaceted nature of health literacy. However, the heterogeneity of the health literacy measures is reflected in the modest correlations between these tests (largest $r = 0.445$). The health literacy tests correlated more strongly with fluid ability than with each other which provides further support that health literacy and cognitive function overlap.

For peer review only - http://bmjopen.bmj.com/site/about/guidelines.xhtml

This analysis only examined the association between health literacy and all-cause mortality. It is possible that there are different relationships between health literacy and cause-specific mortality, for example health literacy may only predict deaths linked to unhealthy lifestyles, such as cardiovascular disease. The follow-up period in this study was relatively short, and therefore only a small percentage of participants had died. Future studies should investigate mortality over a longer follow-up period and in larger samples to examine whether there are different patterns of association between health literacy and cause-specific mortality.

We investigated the role that childhood cognitive ability and fluid ability in older age play in the association between health literacy and mortality. The results indicate that fluid-type cognitive capability may account for the association between health literacy and mortality, while childhood cognitive ability—an indicator of lifelong intelligence—does not. Researchers and clinicians should be aware that lower health literacy scores may actually reflect lower cognitive ability in older age, and that current cognitive capacity in older adulthood, but not lifelong intelligence, may be driving the association between health literacy and mortality. Future research examining the association between health literacy and mortality, and other health indicators, should also include measures of cognitive ability to be able to properly disentangle the relationship between health literacy and health.

For peer review only

Contributors CFR discussed and planned the study and analyses, analysed the data, interpreted the data and drafted the initial manuscript. JMS discussed and planned the study and analyses, interpreted the data and contributed to the manuscript. IJD discussed and planned the study and analyses, interpreted the data and contributed to the manuscript.

Funding This work was supported by the University of Edinburgh Centre for Cognitive Ageing and Cognitive Epidemiology, part of the cross council Lifelong Health and Wellbeing Initiative, funded by the Biotechnology and Biological Sciences Research Council (BBSRC), and Medical Research Council (MRC) (grant number MR/K026992/1). The Lothian Birth Cohort 1936 (LBC1936) is supported by Age UK (Disconnected Mind project).

Completing interests None declared.

Ethical approval Ethical approval was obtained from the Scotland A Research Ethics Committee (07/MRE00/58).

Data sharing statement Lothian Birth Cohort 1936 data can be requested from the Lothian Birth Cohort 1936 research team, following completion of a data request application. More information can be found at: http://www.lothianbirthcohort.ed.ac.uk/content/collaboration

Acknowledgements We thank the LBC1936 participants and LBC1936 research team members.

For peer review only - http://bmjopen.bmj.com/site/about/guidelines.xhtml

REFERENCES

1 Institute of Medicine. Health Literacy: A Prescription to End Confusion. Washinton, DC: The National Academies Press, 2004.

2 Chin J, Morrow DG, Stine-Morrow EA, et al. The process-knowledge model of health literacy: evidence from a componential analysis of two commonly used measures. *J Health Commun* 2011;16 Suppl 3:222-41. doi:10.1080/10810730.2011.604702 [published Online First: 27 September 2011].

3 Morrow D, Chin J. Decision Making and Health Literacy among Older Adults. In: Strough TMGN, Löckenhoff C, eds. Aging and Decision Making: Empirical and Applied Perspectives. London: Academic Press, 2015:261-82.

4 Baker DW. The meaning and the measure of health literacy. *J Gen Intern Med* 2006;21(8):878-83. doi:10.1111/j.1525-1497.2006.00540.x

5 Parker RM, Baker DW, Williams MV, et al. The test of functional health literacy in adults. *J Gen Intern Med* 1995;10(10):537-41.

6 Berkman ND, Sheridan SL, Donahue KE, et al. Low health literacy and health outcomes: an updated systematic review. *Ann Intern Med* 2011;155(2):97-107. doi:10.7326/0003-4819-155-2-201107190-00005 [published Online First: 19 July 2011].

7 Kobayashi LC, Wardle J, von Wagner C. Limited health literacy is a barrier to colorectal cancer screening in England: evidence from the English Longitudinal Study of Ageing. *Prev Med* 2014;61:100-105. doi:10.1016/j.ypmed.2013.11.012 [published Online First: 25 November 2013].

8 Wagner CV, Knight K, Steptoe A, et al. Functional health literacy and health-promoting behaviour in a national sample of British adults. *J Epidemiol Community Health* 2007;61(12):1086-90. doi:10.1136/jech.2006.053967.

For peer review only - http://bmjopen.bmj.com/site/about/guidelines.xhtml

9 Cattell RB. Theory of fluid and crystallized intelligence: A critical experiment. *J Educ Psychol* 1963;54(1):1-22. doi:10.1037/h0046743.

10 Horn JL. A basis for research on age differences in cognitive capabilities. In: McArdle JJ, Woodcock RW, eds. Human cognitive abilities in theory and practice. New York: Routledge, 1998:57-91.

11 Park DC, Reuter-Lorenz P. The adaptive brain: aging and neurocognitive scaffolding. *Annu Rev Psychol* 2009;60:173-96. doi:10.1146/annurev.psych.59.103006.093656.

12 Salthouse TA. Selective review of cognitive aging. *J Int Neuropsychol Soc* 2010;16(5):754-60. doi:10.1017/s1355617710000706.

13 Schaie KW. Intellectual development in adulthood: The Seattle longitudinal study. Cambridge: Cambridge University Press, 1996.

14 Bennett JS, Boyle PA, James BD, et al. Correlates of health and financial literacy in older adults without dementia. *BMC Geriatr* 2012;12:30. doi:10.1186/1471-2318-12-30 [published Online First: 12 June 2012].

15 Dahlke AR, Curtis LM, Federman AD, et al. The mini mental status exam as a surrogate measure of health literacy. *J Gen Intern Med* 2014;29(4):615-20. doi:10.1007/s11606-013-2712-x [published Online First: 11 Dec 2013].

16 Federman AD, Sano M, Wolf MS, et al. Health literacy and cognitive performance in older adults. *J Am Geritar Soc* 2009;57(8):1475-80. doi:10.1111/j.1532-5415.2009.02347.x [published Online First: 8 June 2009]

17 Kobayashi LC, Wardle J, Wolf MS, et al. Cognitive Function and Health Literacy Decline in a Cohort of Aging English Adults. *J Gen Intern Med* 2015;30(7):958-64. doi:10.1007/s11606-015-3206-9 [published Online First: 14 February 2015].

18 Kobayashi LC, Smith SG, O'Conor R, et al. The role of cognitive function in the relationship between age and health literacy: a cross-sectional analysis of older adults in

For peer review only - http://bmjopen.bmj.com/site/about/guidelines.xhtml

Chicago, USA. *BMJ Open* 2015;5(4):e007222. doi:10.1136/bmjopen-2014-007222 [published Online First: 23 April 2015].

19 Levinthal BR, Morrow DG, Tu W, et al. Cognition and health literacy in patients with hypertension. *J Gen Intern Med* 2008;23(8):1172-6. doi:10.1007/s11606-008-0612-2 [published Online First:6 May 2008].

20 Murray C, Johnson W, Wolf MS, et al. The Association between Cognitive Ability across the Lifespan and Health Literacy in Old Age: The Lothian Birth Cohort 1936. *Intelligence* 2011;39(4):178-87. doi:10.1016/j.intell.2011.04.001 [published Online First: 4 May 2011].

21 Ownby RL, Acevedo A, Waldrop-Valverde D, et al. Abilities, skills and knowledge in measures of health literacy. *Patient Educ Couns* 2014;95(2):211-7. doi:10.1016/j.pec.2014.02.002 [published Online First: 16 February 2014].

22 Mõttus R, Johnson W, Murray C, et al. Towards understanding the links between health literacy and physical health. *Health Psychol* 2014;33(2):164-73. doi:10.1037/a0031439.

23 Reeve CL, Basalik D. Is health literacy an example of construct proliferation? A conceptual and empirical evaluation of its redundancy with general cognitive ability. *Intelligence* 2014;44:93-102. doi:10.1016/j.intell.2014.03.004[published Online First: 16 April 2014].

24 O'Conor R, Wolf MS, Smith SG, et al. Health literacy, cognitive function, proper use, and adherence to inhaled asthma controller medications among older adults with asthma. *Chest* 2015;147(5):1307-15. doi:10.1378/chest.14-0914 [published Online First: 2 October 2014].

25 Serper M, Patzer RE, Curtis LM, et al. Health Literacy, Cognitive Ability, and Functional Health Status among Older Adults. *Health Serv Res* 2014;49(4):1249-67. doi:10.1111/1475-6773.12154 [published Online First: 30 Jan 2014].

For peer review only

For peer review only - http://bmjopen.bmj.com/site/about/guidelines.xhtml

26 Murray C, Pattie A, Starr JM, et al. Does Cognitive Ability Predict Mortality in the Ninth Decade? The Lothian Birth Cohort 1921. *Intelligence* 2012;40(5):490-98. doi:10.1016/j.intell.2012.05.003 [published Online First: 12 June 2012].

27 Pavlik VN, de Moraes SA, Szklo M, et al. Relation between cognitive function and mortality in middle-aged adults: the atherosclerosis risk in communities study. *Am J Epidemiol* 2003;157(4):327-34. doi:10.1093/aje/kwf209.

28 Baker DW, Wolf MS, Feinglass J, et al. Health literacy and mortality among elderly persons. *Arch Intern Med* 2007;167(14):1503-9. doi:10.1001/archinte.167.14.1503.

29 Baker DW, Wolf MS, Feinglass J, et al. Health literacy, cognitive abilities, and mortality among elderly persons. *J Gen Intern Med* 2008;23(6):723-6. doi:10.1007/s11606-008-0566-4 [published Online First: 11 March 2008].

30 Bostock S, Steptoe A. Association between low functional health literacy and mortality in older adults: longitudinal cohort study. *BMJ* 2012;344:e1602. doi:10.1136/bmj.e1602[published Online First: 15 March 2012].

31 Scottish Council for Research in Education. The trend of Scottish intelligence: A comparison of the 1947 and 1932 surveys of intelligence of eleven-year-old pupils. London, UK: University of London Press, 1949.

32 Deary IJ, Gow AJ, Taylor MD, et al. The Lothian Birth Cohort 1936: a study to examine influences on cognitive ageing from age 11 to age 70 and beyond. *BMC Geriatr* 2007;**7**:28. doi: 10.1186/1471-2318-7-28 [published Online First: 5 December 2007].

33 Deary IJ, Gow AJ, Pattie A, et al. Cohort profile: the Lothian Birth Cohorts of 1921 and 1936. *Int J Epidemiol* 2012;41(6):1576-84. doi:10.1093/ije/dyr197 [published Online First: 14 December 2011].

34 Davis TC, Long SW, Jackson RH, et al. Rapid estimate of adult literacy in medicine: a shortened screening instrument. *Fam Med* 1993;25(6):391-95.

For peer review only - http://bmjopen.bmj.com/site/about/guidelines.xhtml

35 Dumenci L, Matsuyama RK, Kuhn L, et al. On the Validity of the Rapid Estimate of Adult Literacy in Medicine (REALM) Scale as a Measure of Health Literacy. *Commun Methods Meas* 2013;7(2):134-43. doi:10.1080/19312458.2013.789839[published Online First: Epub Date]|.

36 Baker DW, Williams MV, Parker RM, et al. Development of a brief test to measure functional health literacy. *Patient Educ Couns* 1999;38(1):33-42.

37 Mancuso JM. Assessment and measurement of health literacy: an integrative review of the literature. *Nurs Health Sci* 2009;11(1):77-89. doi:10.1111/j.1442-2018.2008.00408.x [published Online First: 10 February 2009].

38 Weiss BD, Mays MZ, Martz W, et al. Quick assessment of literacy in primary care: the newest vital sign. *Ann Fam Med* 2005;3(6):514-22. doi:10.1370/afm.405.

39 Wechsler D. WAIS-III UK administration and scoring manual. London, UK: Psychological Corporation, 1998.

40 General Register Office. Census 1951: Classification of Occupatioans. London, UK, 1956.

41 Zigmond AS, Snaith RP. The Hospital Anxiety and Depression Scale. *Acta Psychiatr Scand* 1983;67(6):361-70. doi:10.1111/j.1600-0447.1983.tb09716.x.

42 Townsend P. Poverty in the United Kingdom: a survey of household resources and standards of living. Harmondsworth, UK: Penguin, 1979.

43 Folstein MF, Folstein SE, McHugh PR. “Mini-mental state”: a practical method for grading the cognitive state of patients for the clinician. *J Psychiatr Res* 1975;12(3):189-98. doi:10.1016/0022-3956(75)90026-6.

For peer review only - http://bmjopen.bmj.com/site/about/guidelines.xhtml

Supplementary material for: The role of cognitive ability in the association between health literacy and mortality in the Lothian Birth Cohort 1936: a prospective cohort study

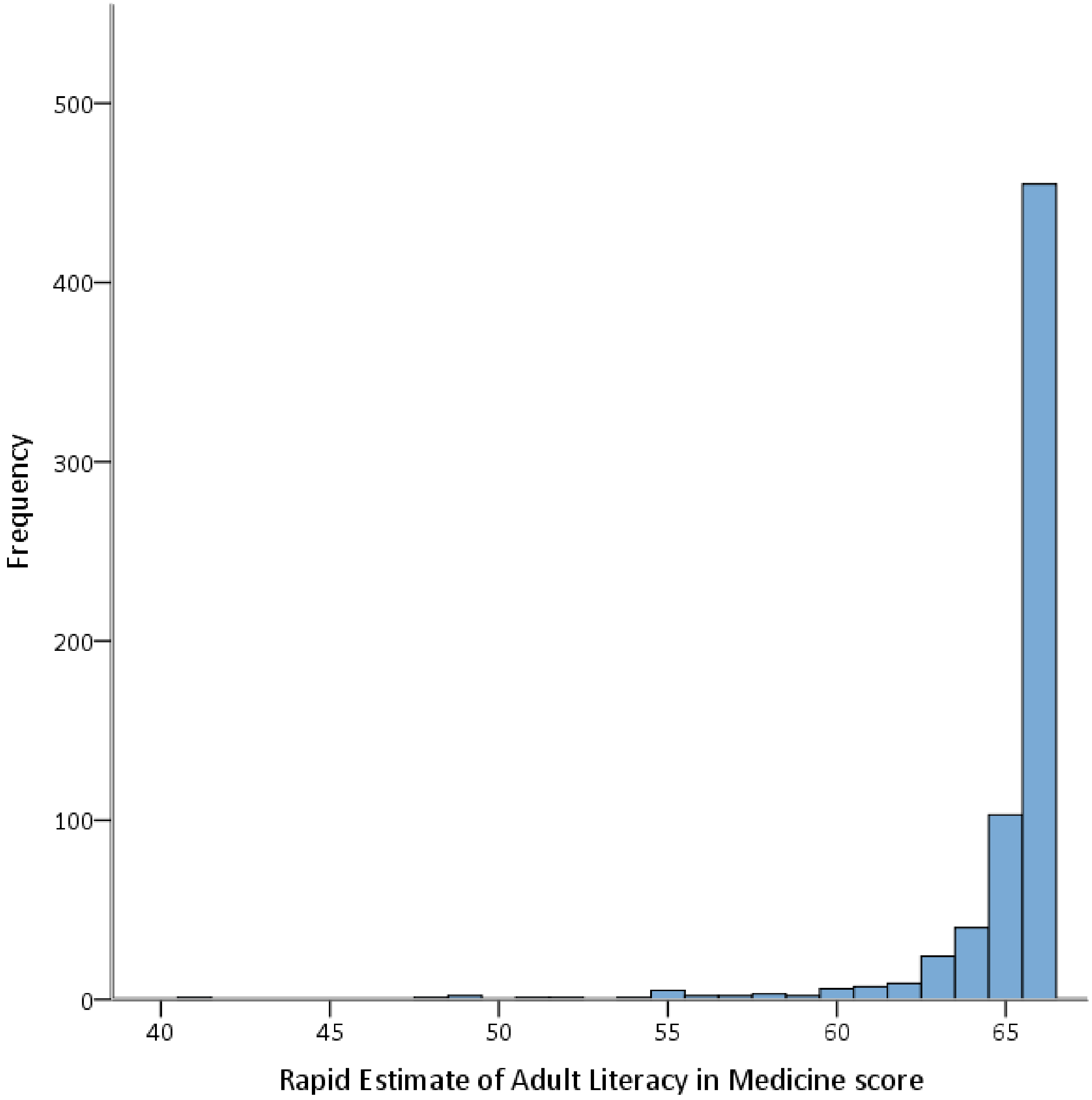

Supplementary Figure 1 Distribution of scores on the Rapid Estimate of Adult Literacy in Medicine for participants who were alive at censoring date.

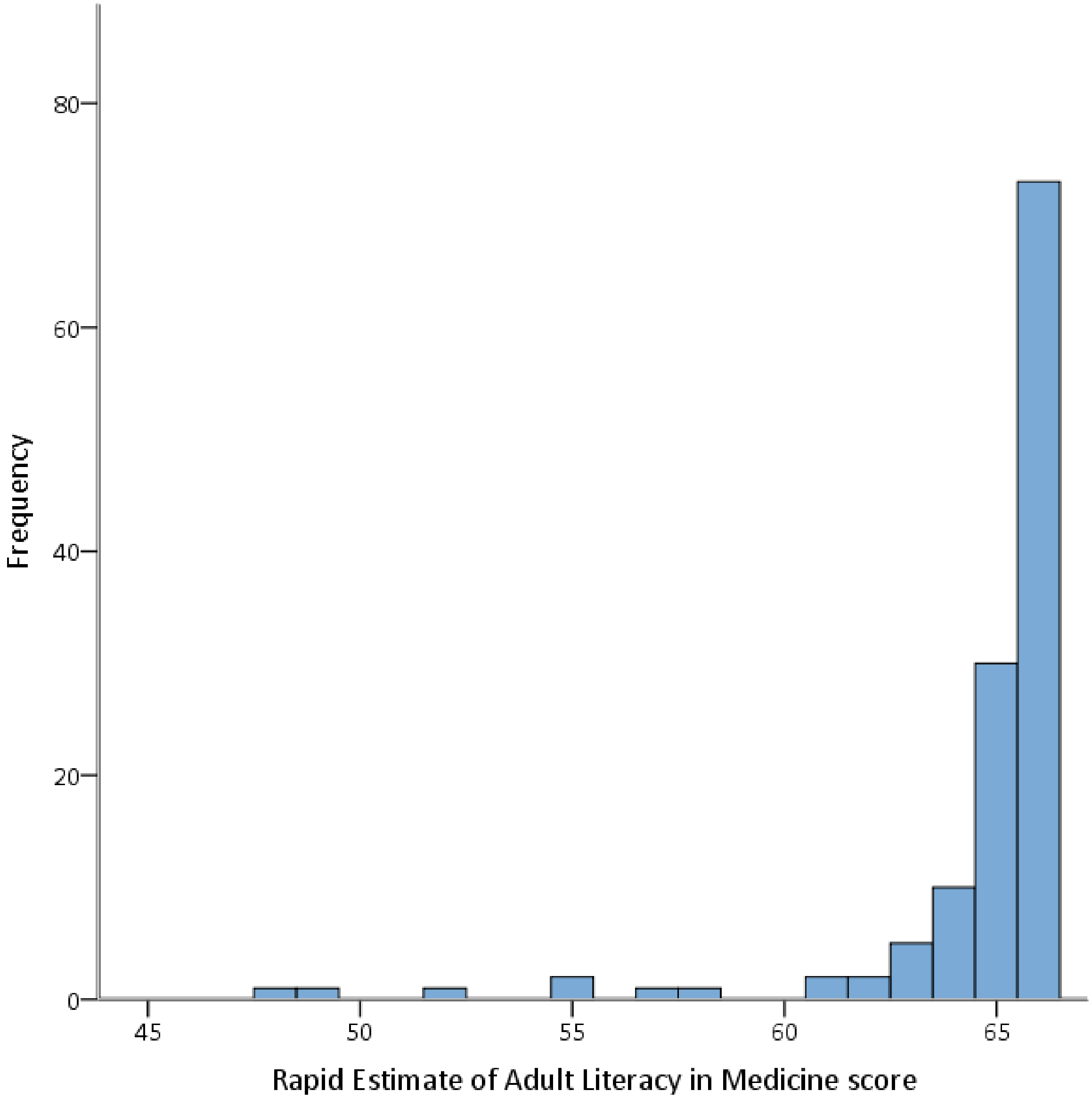

Supplementary Figure 2 Distribution of scores on the Rapid Estimate of Adult Literacy in Medicine for participants who had died by censoring date.

For peer review only - http://bmjopen.bmj.com/site/about/guidelines.xhtml

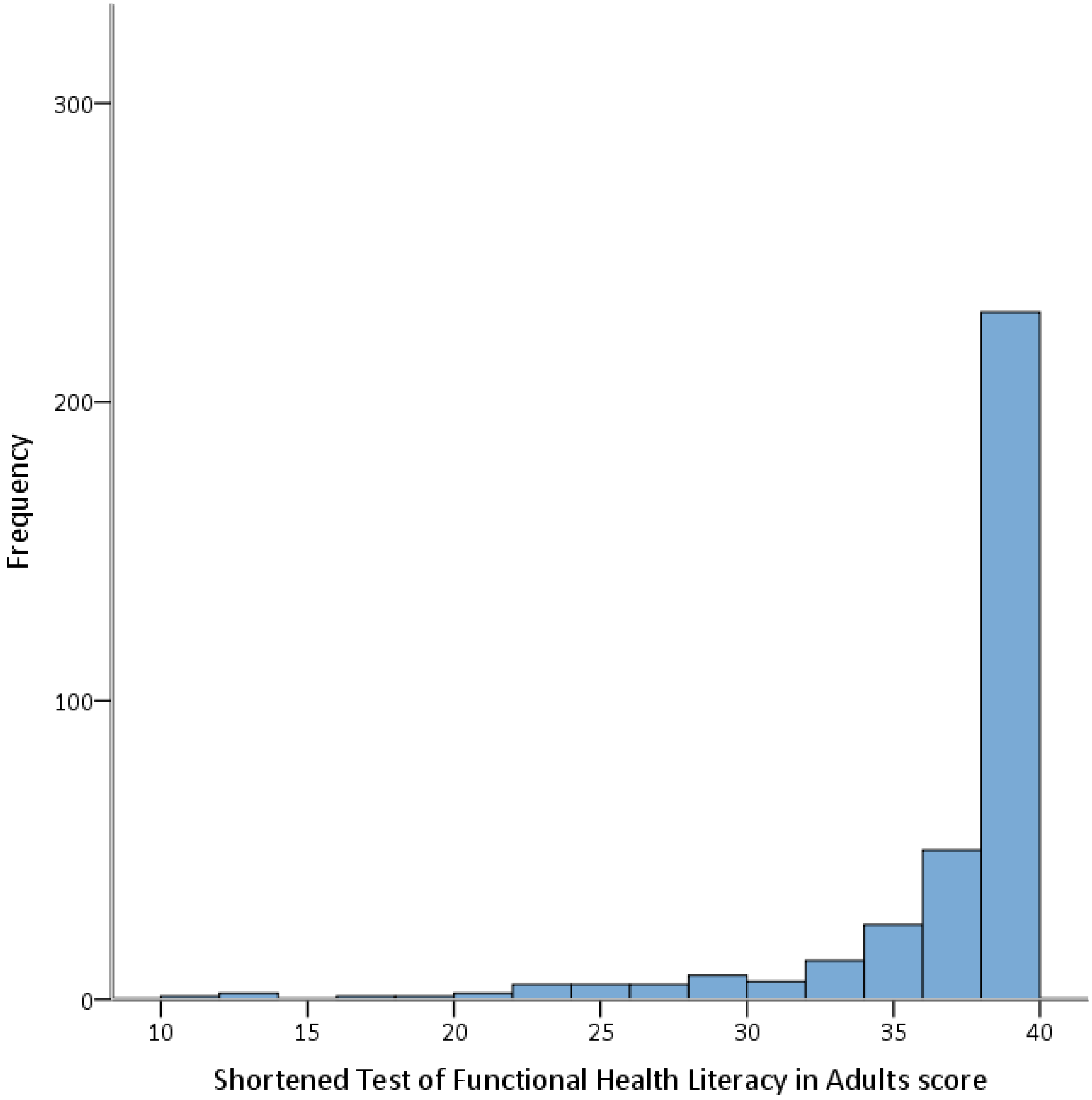

Supplementary Figure 3 Distribution of scores on the Shortened Test of Functional Health Literacy in Adults for participants who were alive at censoring date.

For peer review only - http://bmjopen.bmj.com/site/about/guidelines.xhtml

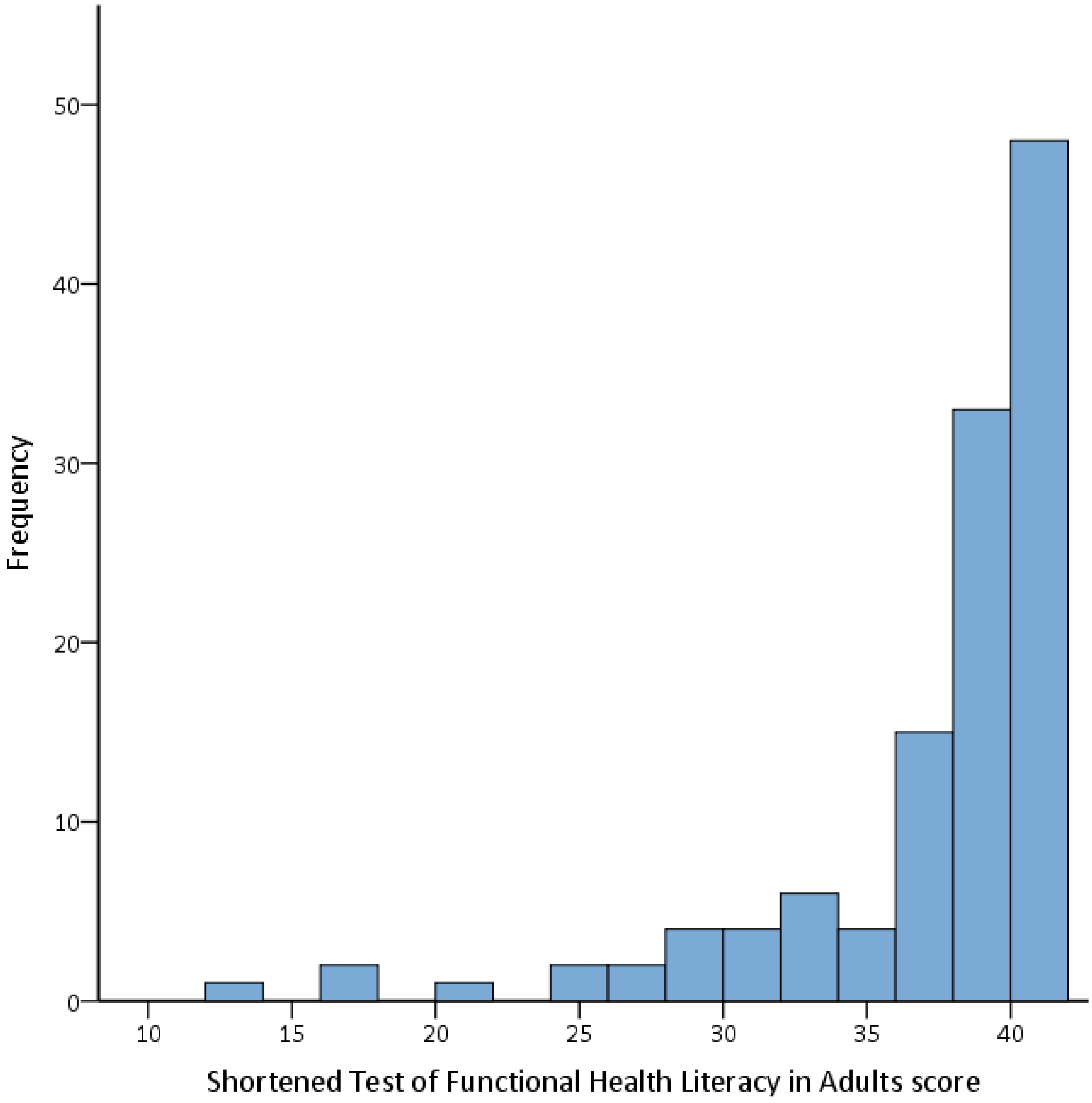

Supplementary Figure 4 Distribution of scores on the Shortened Test of Functional Health Literacy in Adults for participants who has died by censoring date.

For peer review only - http://bmjopen.bmj.com/site/about/guidelines.xhtml

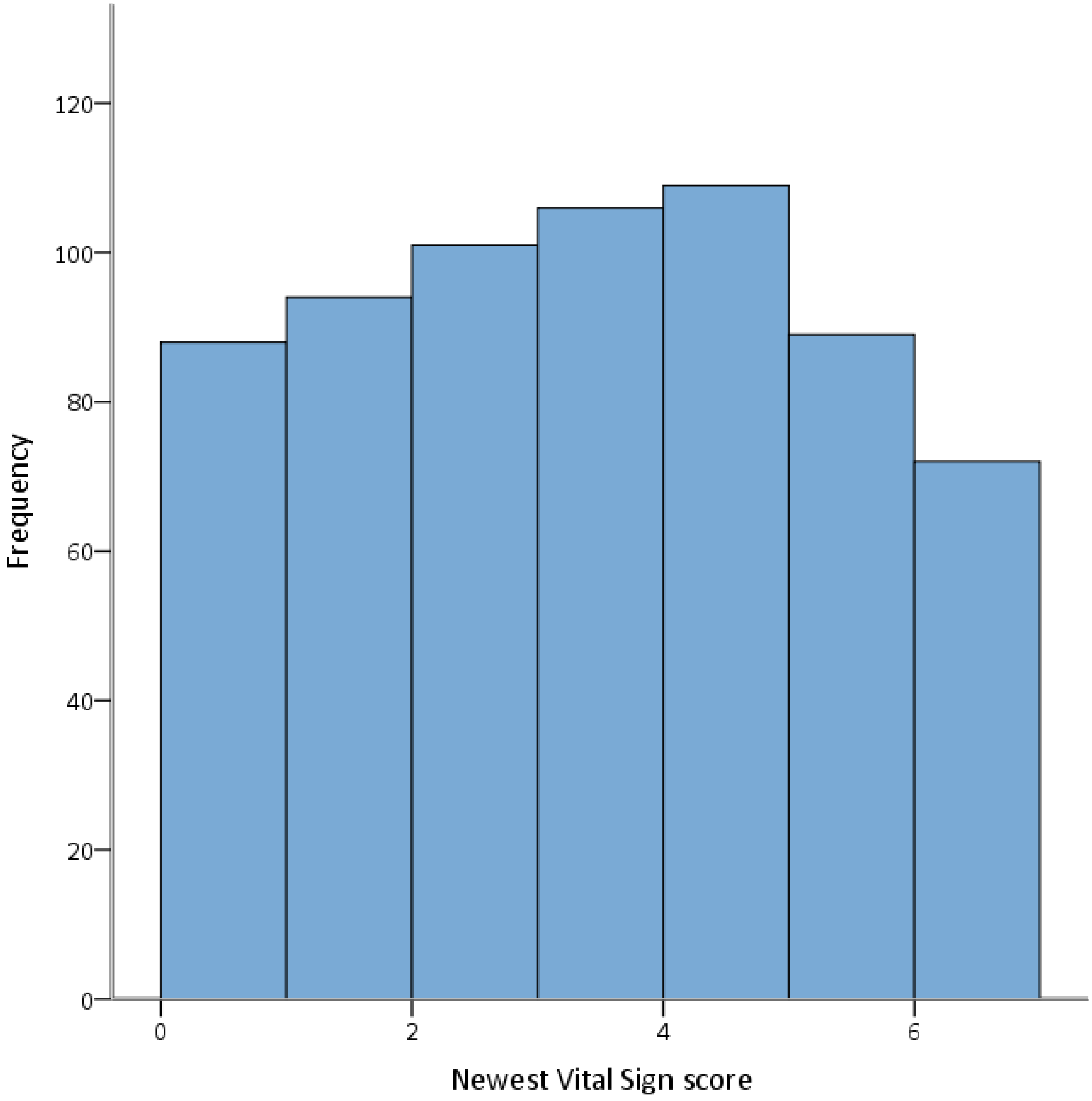

Supplementary Figure 5 Distribution of scores on the Newest Vital Sign for participants who were alive at censoring date.

For peer review only - http://bmjopen.bmj.com/site/about/guidelines.xhtml

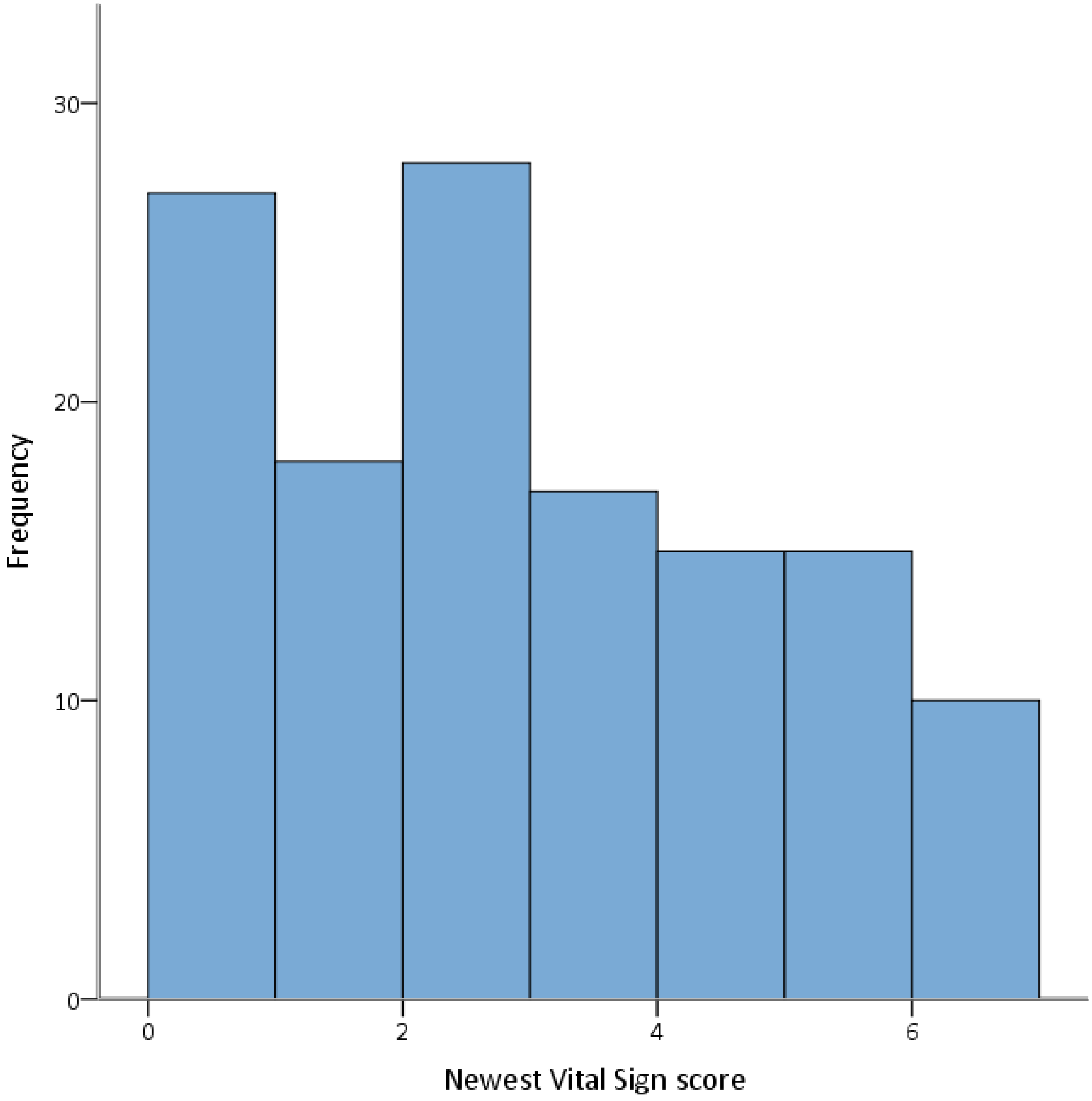

Supplementary Figure 6 Distribution of scores on the Newest Vital Sign for participants who had died by censoring date.

For peer review only - http://bmjopen.bmj.com/site/about/guidelines.xhtml

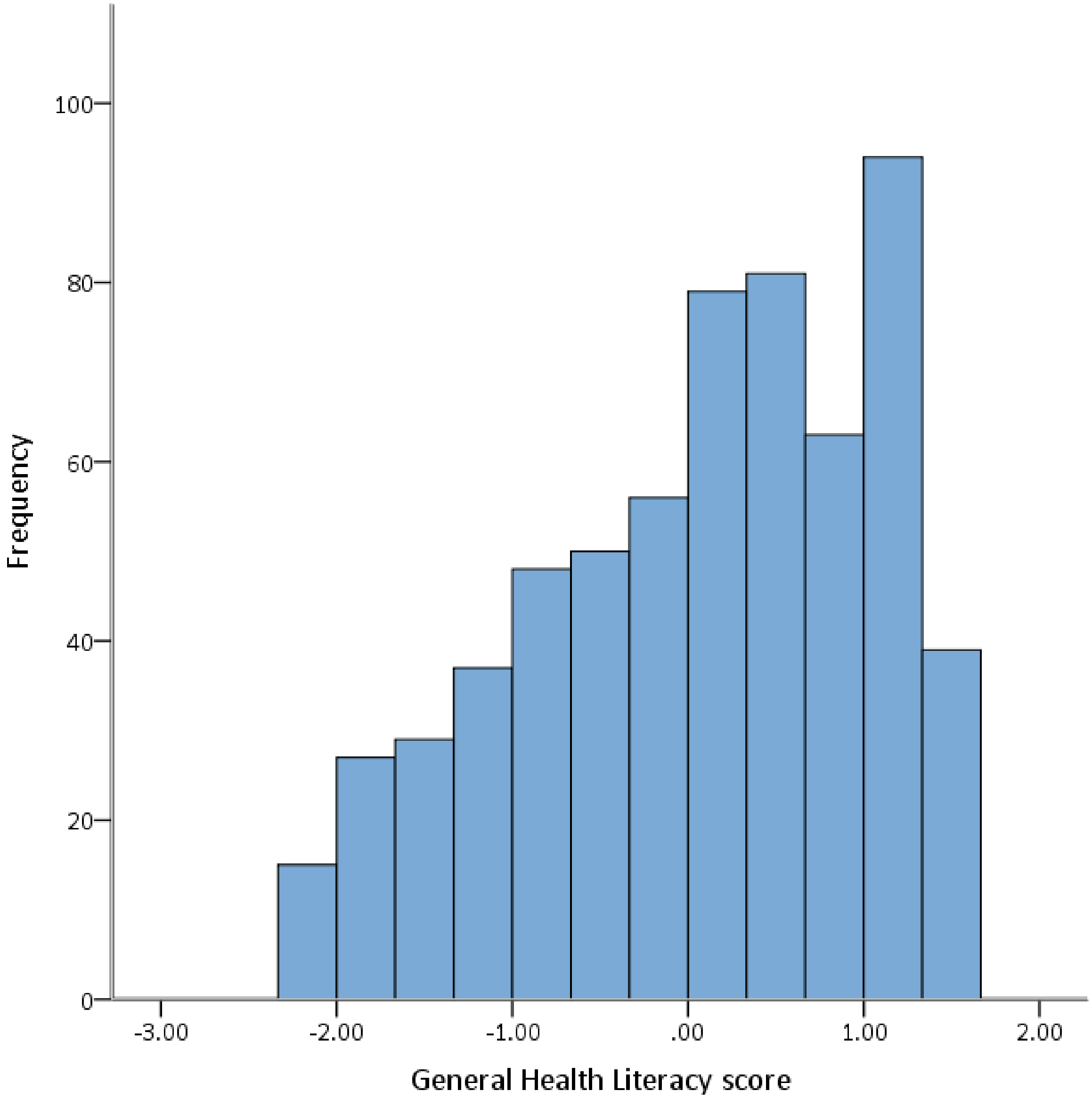

Supplementary Figure 7 Distribution of scores on General Health Literacy for participants who were alive at censoring date.

For peer review only - http://bmjopen.bmj.com/site/about/guidelines.xhtml

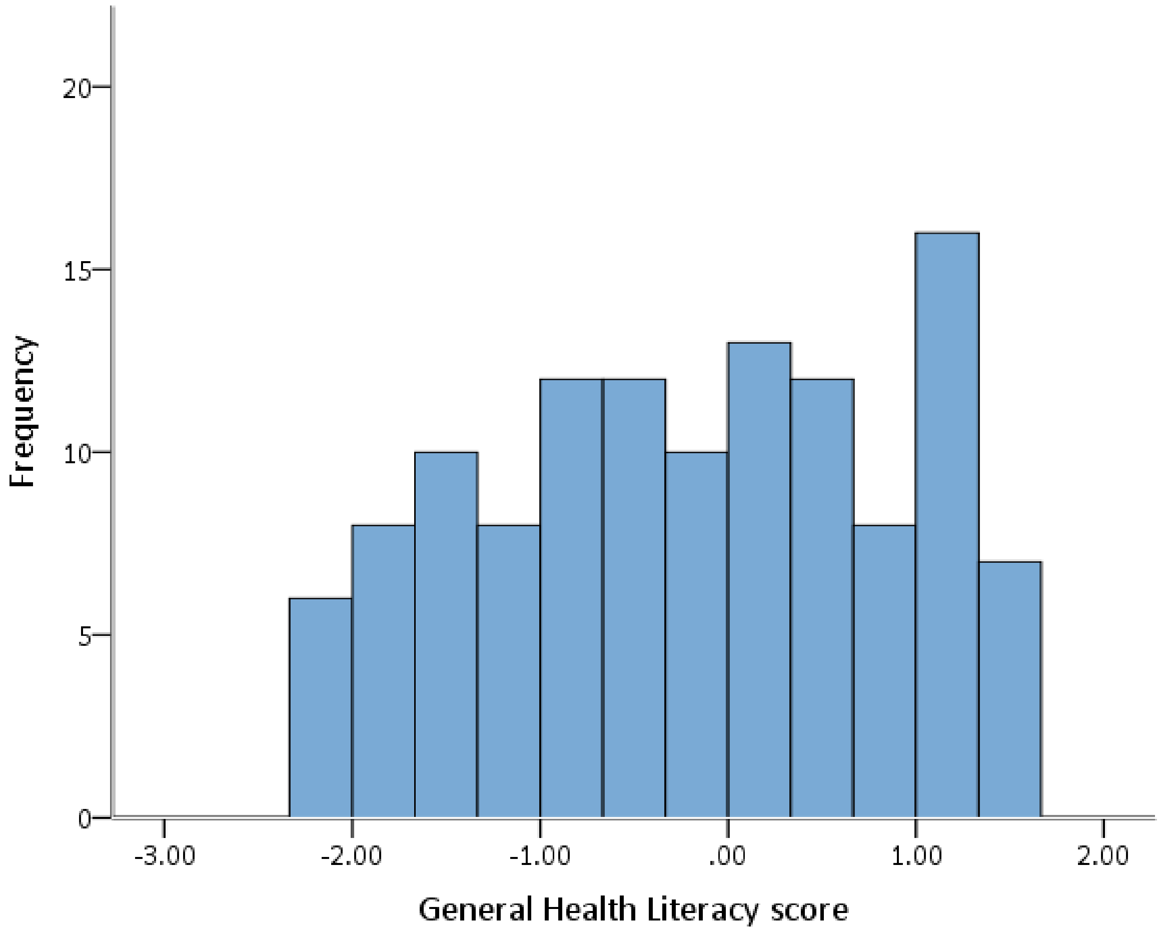

Supplementary Figure 8 Distribution of scores on General Health Literacy for participants who had died by censoring date.

For peer review only - http://bmjopen.bmj.com/site/about/guidelines.xhtml

Supplementary Table 1 Rank order correlations between sociodemographic, health literacy, cognitive and health variables

	1	2	3	4	5	6	7	8	9	10	11	12	13
1 Age	1.000												
2 Sex†	0.044	1.000											
3 REALM	-0.119**	0.167***	1.000										
4 S-TOFHLA	-0.050	0.101**	0.398***	1.000									
5 NVS	-0.115**	0.012	0.348***	0.444***	1.000								
6 General health literacy	-0.094*	0.136***	0.706***	0.803***	0.781***	1.000							
7 Age-11 IQ	-0.072*	0.105**	0.438***	0.481***	0.513***	0.611***	1.000						
8 Fluid ability	-0.133***	0.001	0.378***	0.550***	0.549***	0.632***	0.565***	1.000					
9 Education	-0.054	0.033	0.314***	0.333***	0.368***	0.451***	0.454***	0.368***	1.000				
10 Occup class	0.046	-0.150***	-0.305***	-0.310***	-0.317***	-0.388***	-0.403***	-0.352***	-0.466***	1.000			
11 Self-rated health	-0.023	0.059	0.118**	0.196***	0.110**	0.184***	0.168***	0.235***	0.110**	-0.107**	1.000		
12 HADS	0.057	0.076*	-0.065	-0.127**	-0.109**	-0.137***	-0.129***	-0.222***	-0.082*	0.077*	-0.320***	1.000	
13 Townsend	0.127***	0.159***	-0.077*	-0.119**	-0.154***	-0.140***	-0.118**	-0.174***	-0.122**	0.092*	-0.349***	0.218***	1.000

*$p < .05$, **$p < .01$, ***$p < .001$.

†Correlations are point-biseral correlations. Female is coded 1 and male is coded 2.

Occupational class (ranging from 1-professional to 4-manual) and self-rated health (ranging from 1-poor/fair to 3-very good/excellent) are entered as ordinal variables.

REALM, Rapid Estimate of Adult Literacy in Medicine; S-TOFHLA, Shortened Test of Functional Health Literacy in Adults; NVS, Newest Vital Sign; IQ, Intelligence Quotient; Occup class, occupational class; HADS, Hospital Anxiety and Depression Scale; Townsend, Townsend Disability Scale.

For peer review only

DETAILED RESULTS

REALM: Hazard ratios (HRs) and 95% confidence intervals (CIs) for the associations between the REALM and mortality are shown in Supplementary Table 2. In Model 1, in which age and sex were controlled, the REALM did not significantly predict mortality (HR = 0.954, 95% CI 0.904 to 1.007), nor did age or sex. The REALM remained a non-significant predictor of mortality in Model 2, with the addition of age-11 IQ. Age-11 IQ did not predict mortality (HR = 0.993, 95% CI 0.981 to 1.006). The REALM remained a non-significant predictor of mortality following the inclusion of current fluid ability in Model 3. A one SD increase in fluid ability score reduced the risk of death by 36.8% (HR = 0.632, 95% CI 0.506 to 0.789). In Model 4, the sociodemographic variables years of education and occupational social class were included in the model. The REALM remained non-significant. Years of education did not predict mortality. Individuals with a managerial/technical social class (HR = 2.278, 95% CI 1.161 to 4.470), a skilled non-manual social class (HR = 2.464, 95% CI 1.167 to 5.201) or a skilled manual social class (HR = 3.608, 95% CI 1.647 to 7.907) had a higher risk of death than individuals with a professional social class. Health status variables were additionally added in Model 5. The REALM remained a non-significant predictor of mortality. Risk of death for those who self-reported their health as fair or poor was over 2 times greater than those who reported their health to be very good or excellent (HR = 2.071, 95% CI 1.147 to 3.739). While HADS score did not predict mortality, Townsend disability did. A one-point increase on the Townsend disability scale increased risk of mortality by 13.3% (HR = 1.133, 95% CI 1.044 to 1.229).

S-TOFHLA: The HRs for the association between S-TOFHLA and mortality are shown in Supplementary Table 3. In Model 1, controlling for age and sex, S-TOFHLA significantly predicted mortality. A one-point increase in S-TOFHLA reduced the risk of death by 5.2% (HR = 0.948, 95% CI 0.919 to 0.978). In this model, age and sex did not predict mortality. The inclusion of age-11 IQ in Model 2 did not change the association between the S-TOFHLA and mortality. Age-11 IQ also did not predict mortality (HR = 0.999, 95% CI 0.986 to 1.012). The association between the S-TOFHLA and mortality was attenuated and became non-significant (HR = 0.967, 95% CI 0.929 to 1.007) in Model 3, additionally accounting current fluid ability. Current fluid ability significantly predicted

For peer review only - http://bmjopen.bmj.com/site/about/guidelines.xhtml

mortality in this model. A one SD increase in fluid ability reduced the risk of death by 28.9% (HR = 0.711, 95% CI 0.558 to 0.905). Sociodemographic variables were included in Model 4, and the association between S-TOFHLA and mortality remained non-significant. Individuals with more years of education, controlling for other sociodemographic variables and cognitive function, had increased risk of death (HR = 1.219, 95% CI 1.004 to 1.481). Risk of dying was three times greater for participants with a skilled manual social class, compared to individuals with a professional social class (HR = 3.096, 95% CI 1.385 to 6.922). S-TOFHLA remained a non-significant predictor or mortality in Model 5, which included health status variables. Self-reporting health as fair or poor, compared to very good or excellent, was associated with increased risk of mortality (HR = 2.209, 95% CI 1.216 to 4.014). Higher scores on the HADS were not associated with mortality, while a higher Townsend disability score increased risk of death (HR = 1.131, 95% CI 1.039 to 1.232).

NVS: HRs for the association between NVS and mortality are shown in Supplementary Table 4. In Model 1, in which age and sex were entered as covariates, NVS significantly predicted mortality. A one point increase in NVS score reduced the risk of death by 11.8% (HR = 0.882, 95% CI 0.805 to 0.966). Age and sex did not predict mortality. Age-11 IQ was additionally added to the model in Model 2 and this did little to change the association between NVS and mortality and this association remained significant. Age-11 IQ did not predict mortality (HR = 0.996, 95% CI 0.984 to 1.009). The inclusion of fluid ability in Model 3 greatly attenuated the association between NVS and mortality, and this became non-significant (HR = 0.972, 95% CI 0.869 to 1.087). Fluid ability was strongly associated with risk of death. A one SD increase in fluid ability score reduced risk of dying by 36.6% (HR = 0.637, 95% CI 0.501 to 0.809). The association between NVS and mortality remained non-significant in Model 4 following the inclusion of sociodemographic variables in the model. Years of education did not predict mortality. Compared to those with a professional social class, participants with managerial or technical (HR = 2.288, 95% CI 1.166 to 4.490), skilled non-manual (HR = 2.421, 95% CI 1.147 to 5.112), and skilled manual (HR = 3.631, 95% CI 1.658 to 7.951) social class had an increased risk of death. Finally, health status variables were included in Model 5. The inclusion of health status variables did little to change the association between NVS and mortality, which

For peer review only - http://bmjopen.bmj.com/site/about/guidelines.xhtml

remained non-significant. Those who reported their health as fair or poor had 2.10 times (HR = 2.099, 95% CI 1.167 to 3.775) increased risk of mortality, compared to those who self-reported their health as very good or excellent. Participants with higher scores on the Townsend disability scale also had an increased risk of mortality (HR = 1.132, 95% CI 1.044 to 1.228).

General health literacy: HRs for the association between general health literacy and mortality are shown in Supplementary Table 5. General health literacy predicted mortality in Model 1 (HR = 0.774, 95% CI 0.650 to 0.922), while age and sex did not. A one point increase in the general health literacy score reduced the risk of mortality by 22.6%. General health literacy remained a significant predictor of mortality when age-11 IQ was added in Model 2. Age-11 IQ did not predict morality (HR = 1.001, 95% CI 0.986 to 1.015). The inclusion of current fluid ability in Model 3 attenuated the association between general health literacy and risk of death, and this association became non-significant (HR = 0.901, 95% CI 0.701 to 1.158). Fluid ability was a significant predictor of mortality, such that a one SD increase in fluid ability reduced risk of death by 30.7% (HR = 0.693, 95% CI 0.536 to 0.895). Including years of education and occupational social class in Model 4 did little to change the association between general health literacy and mortality, and this association remained non-significant. In Model 4, individuals with more years of education had a greater risk of death (HR = 1.240, 95% CI 1.019 to 1.508), and those with an occupational social class of skilled manual (HR = 3.134, 95% CI 1.405 to 6.991), when compared to those with a professional occupational class, had an increased risk of mortality. Finally, health status variables were added in Model 5. The association between general health literacy and mortality was attenuated further and remained non-significant. Reporting fair or poor health, compared to reporting very good or excellent health increased the risk of mortality (HR = 2.229, 95% CI 1.229 to 4.042). Higher Townsend disability scores were also associated with increased risk of death (HR = 1.128, 95% CI 1.040 to 1.225). In this final model, controlling for sociodemographics and health variables, as well as age-11 IQ, the association between fluid ability and mortality was attenuated and became non-significant (HR = 0.770, 95% CI 0.589 to 1.007).

For peer review only - http://bmjopen.bmj.com/site/about/guidelines.xhtml

Supplementary Table 2 Hazard ratios (95% confidence intervals) for the association between REALM and mortality, controlling for sociodemographic, cognitive ability, and health status variables

	Model 1<br>Age and sex<br>N = 794	Model 2<br>+ age-11 IQ<br>N = 752	Model 3<br>+ current fluid ability<br>N = 746	Model 4<br>+ sociodemographics<br>N = 731	Model 5<br>+ health status<br>N = 728
REALM	0.954 (0.904 to 1.007)	0.962 (0.903 to 1.025)	0.972 (0.909 to 1.040)	0.970 (0.904 to 1.040)	0.996 (0.924 to 1.074)
Age	0.940 (0.725 to 1.219)	0.944 (0.724 to 1.230)	0.880 (0.670 to 1.156)	0.908 (0.686 to 1.203)	0.933 (0.704 to 1.235)
Sex					
Female	Reference	Reference	Reference	Reference	Reference
Male	1.297 (0.909 to 1.850)	1.254 (0.871 to 1.805)	1.330 (0.924 to 1.913)	1.176 (0.787 to 1.756)	1.364 (0.898 to 2.073)
Age-11 IQ		0.993 (0.981 to 1.006)	1.010 (0.995 to 1.025)	1.009 (0.994 to 1.024)	1.008 (0.993 to 1.024)
Fluid ability			0.632 (0.506-0.789)***	0.662 (0.526 to 0.834)***	0.727 (0.574 to 0.922)**
Years of education				1.201 (0.995 to 1.450)	1.232 (1.018 to 1.492)*
Occupational class					
Professional				Reference	Reference
Managerial/technical				2.278 (1.161 to 4.470)*	2.218 (1.127 to 4.365)*
Skilled: non-manual				2.464 (1.167 to 5.201)*	2.596 (1.232 to 5.474)*
Skilled: manual				3.608 (1.647 to 7.907)**	3.393 (1.532 to 7.516)**
Partly skilled/ unskilled manual				2.054 (0.651 to 6.473)	2.067 (0.656 to 6.510)
Self-rated health					
Very good/excellent					Reference
Good					1.153 (0.742 to 1.791)
Fair/poor					2.071 (1.147 to 3.739)*
HADS total score					0.972 (0.929 to 1.018)
Townsend disability					1.133 (1.044 to 1.229)**

* $p < .05$, ** $p < .01$, *** $p < .001$.

REALM, Rapid Estimate of Adult Literacy in Medicine; IQ, Intelligence Quotient; HADS, Hospital Anxiety and Depression Scale.

For peer review only - http://bmjopen.bmj.com/site/about/guidelines.xhtml

Supplementary Table 3 Hazard ratios (95% confidence intervals) for the association between S-TOFHLA and mortality, controlling for sociodemographic, cognitive ability, and health variables

	Model 1<br>Age and sex<br>N = 744	Model 2<br>+ age-11 IQ<br>N = 702	Model 3<br>+ current fluid ability<br>N = 697	Model 4<br>+ sociodemographics<br>N = 682	Model 5<br>+ health status<br>N = 680
S-TOFHLA	0.948 (0.919 to 0.978)**	0.948 (0.913 to 0.983)**	0.967 (0.929 to 1.007)	0.976 (0.935 to 1.019)	0.998 (0.953 to 1.046)
Age	0.882 (0.665 to 1.170)	0.888 (0.666 to 1.185)	0.871 (0.652 to 1.164)	0.919 (0.682 to 1.238)	0.936 (0.697 to 1.256)
Sex					
Female	Reference	Reference	Reference	Reference	Reference
Male	1.307 (0.909 to 1.879)	1.277 (0.881 to 1.852)	1.339 (0.924 to 1.942)	1.204 (0.797 to 1.818)	1.352 (0.881 to 2.074)
Age-11 IQ		0.999 (0.986 to 1.012)	1.009 (0.994 to 1.024)	1.007 (0.991 to 1.022)	1.006 (0.991 to 1.022)
Fluid ability			0.711 (0.558-0.905)**	0.717 (0.557 to 0.922)*	0.759 (0.587 to 0.982)*
Years of education				1.219 (1.004 to 1.481)*	1.249 (1.026 to 1.520)*
Occupational class					
Professional				Reference	Reference
Managerial/technical				1.889 (0.956 to 3.734)	1.844 (0.931 to 3.650)
Skilled: non-manual				2.108 (0.994 to 4.470)	2.207 (1.042 to 4.673)*
Skilled: manual				3.096 (1.385 to 6.922)**	2.881 (1.275 to 6.509)*
Partly skilled/ unskilled manual				1.786 (0.566 to 5.636)	1.773 (0.562 to 5.598)
Self-rated health					
Very good/excellent					Reference
Good					1.147 (0.728 to 1.807)
Fair/poor					2.209 (1.216 to 4.014)**
HADS total score					0.974 (0.930 to 1.021)
Townsend disability					1.131 (1.039 to 1.232)**

*$p < .05$, **$p < .01$, ***$p < .001$.

S-TOFHLA, Shortened Test of Functional Health Literacy in Adults; IQ, Intelligence Quotient; HADS, Hospital Anxiety and Depression Scale.

For peer review only

For peer review only - http://bmjopen.bmj.com/site/about/guidelines.xhtml

Supplementary Table 4 Hazard ratios (95% confidence intervals) for the association between NVS and mortality, controlling for sociodemographic, cognitive ability, and health variables

	Model 1<br>Age and sex<br>N = 789	Model 2<br>+ age-11 IQ<br>N = 746	Model 3<br>+ current fluid ability<br>N = 742	Model 4<br>+ sociodemographics<br>N = 727	Model 5<br>+ health status<br>N = 724
NVS	0.882 (0.805 to 0.966)**	0.899 (0.810 to 0.997)*	0.972 (0.869 to 1.087)	0.967 (0.861 to 1.086)	0.961 (0.853 to 1.082)
Age	0.942 (0.727 to 1.221)	0.942 (0.722 to 1.229)	0.892 (0.680 to 1.172)	0.919 (0.694 to 1.217)	0.937 (0.708 to 1.242)
Sex					
Female	Reference	Reference	Reference	Reference	Reference
Male	1.343 (0.946 to 1.906)	1.282 (0.895 to 1.838)	1.342 (0.937 to 1.923)	1.180 (0.791 to 1.760)	1.355 (0.893 to 2.057)
Age-11 IQ		0.996 (0.984 to 1.009)	1.009 (0.994 to 1.023)	1.008 (0.993 to 1.023)	1.009 (0.994 to 1.023)
Fluid ability			0.637 (0.501 to 0.809)***	0.670 (0.524 to 0.857)**	0.748 (0.580 to 0.966)*
Years of education				1.208 (0.998 to 1.463)	1.242 (1.023 to 1.508)*
Occupational class					
Professional				Reference	Reference
Managerial/technical				2.288 (1.166 to 4.490)*	2.243 (1.140 to 4.414)*
Skilled: non-manual				2.421 (1.147 to 5.112)*	2.593 (1.231 to 5.463)*
Skilled: manual				3.631 (1.658 to 7.951)**	3.360 (1.522 to 7.415) **
Partly skilled/ unskilled manual				2.125 (0.677 to 6.669)	2.086 (0.661 to 6.578)
Self-rated health					
Very good/excellent					Reference
Good					1.175 (0.756 to 1.826)
Fair/poor					2.099 (1.167 to 3.775)*
HADS total score					0.973 (0.930 to 1.018)
Townsend disability					1.132 (1.044 to 1.228)**

*p < .05, **p < .01.

NVS, Newest Vital Sign; IQ, Intelligence Quotient; HADS, Hospital Anxiety and Depression Scale.

For peer review only - http://bmjopen.bmj.com/site/about/guidelines.xhtml

Supplementary Table 5 Hazard ratios (95% confidence intervals) for the association between general health literacy and mortality, controlling for sociodemographic, cognitive ability, and health variables

	Model 1<br>Age and sex<br>N = 740	Model 2<br>+ age-11 IQ<br>N = 698	Model 3<br>+ current fluid ability<br>N = 694	Model 4<br>+ sociodemographics<br>N = 679	Model 5<br>+ health status<br>N = 677
General health literacy	0.774 (0.650 to 0.922)**	0.766 (0.612 to 0.959)*	0.901 (0.701 to 1.158)	0.911 (0.700 to 1.186)	0.950 (0.725 to 1.245)
Age	0.897 (0.678 to 1.187)	0.906 (0.680 to 1.206)	0.890 (0.666 to 1.188)	0.933 (0.693 to 1.257)	0.942 (0.700 to 1.266)
Sex					
Female	Reference	Reference	Reference	Reference	Reference
Male	1.276 (0.886 to 1.838)	1.248 (0.859 to 1.813)	1.327 (0.913 to 1.930)	1.178 (0.778 to 1.784)	1.337 (0.869 to 2.056)
Age-11 IQ		1.001 (0.986 to 1.015)	1.008 (0.993 to 1.024)	1.006 (0.991 to 1.022)	1.007 (0.992 to 1.023)
Fluid ability			0.693 (0.536 to 0.895)**	0.707 (0.543 to 0.921)*	0.770 (0.589 to 1.007)
Years of Education				1.240 (1.019 to 1.508)*	1.255 (1.030 to 1.528)*
Occupational class					
Professional				Reference	Reference
Managerial/technical				1.901 (0.962 to 3.756)	1.870 (0.945 to 3.700)
Skilled: non-manual				2.076 (0.979 to 4.401)	2.192 (1.035 to 4.640)*
Skilled: manual				3.134 (1.405 to 6.991)**	2.823 (1.252 to 6.365)*
Partly skilled/ unskilled manual				1.824 (0.580 to 5.741)	1.759 (0.557 to 5.561)
Self-rated health					
Very good/excellent					Reference
Good					1.152 (0.733 to 1.810)
Fair/poor					2.229 (1.229 to 4.042)**
HADS total score					0.975 (0.931 to 1.022)
Townsend disability					1.128 (1.040 to 1.225)**

* $p < .05$, ** $p < .01$.

General health literacy, general measure of health literacy created by entering the REALM, S-TOFHLA and NVS into a PCA; IQ, Intelligence Quotient; HADS, Hospital Anxiety and Depression Scale.

For peer review only - http://bmjopen.bmj.com/site/about/guidelines.xhtml

Supplementary Table 6 Hazard ratios (95% confidence intervals) for the association between REALM and mortality, controlling for sociodemographic, cognitive ability, and health status variables. Models are run on a sub-sample participants with all variables of interest (N = 728).

	Model 1<br>Age and sex	Model 2<br>+ age-11 IQ	Model 3<br>+ current fluid ability	Model 4<br>+ sociodemographics	Model 5<br>+ health status
REALM	0.944 (0.894 to 0.997)*	0.959 (0.901 to 1.022)	0.968 (0.905 to 1.035)	0.969 (0.904 to 1.039)	0.996 (0.924 to 1.074)
Age	1.002 (0.763 to 1.316)	0.999 (0.761 to 1.312)	0.931 (0.704 to 1.232)	0.930 (0.700 to 1.234)	0.933 (0.704 to 1.235)
Sex					
Female	Reference	Reference	Reference	Reference	Reference
Male	1.303 (0.897 to 1.892)	1.290 (0.888 to 1.873)	1.356 (0.934 to 1.968)	1.224 (0.815 to 1.836)	1.364 (0.898 to 2.073)
Age-11 IQ		0.993 (0.981 to 1.006)	1.009 (0.994 to 1.024)	1.009 (0.993 to 1.025)	1.008 (0.993 to 1.024)
Fluid ability			0.642 (0.512 to 0.804)***	0.666 (0.528 to 0.841)**	0.727 (0.574 to 0.922)**
Years of education				1.203 (0.994 to 1.455)	1.232 (1.018 to 1.492)*
Occupational class					
Professional				Reference	Reference
Managerial/technical				2.201 (1.118 to 4.333)*	2.218 (1.127 to 4.365)*
Skilled: non-manual				2.482 (1.175 to 5.245)*	2.596 (1.232 to 5.474)*
Skilled: manual				3.570 (1.627 to 7.837)**	3.393 (1.532 to 7.516)**
Partly skilled/ unskilled manual				2.023 (0.641 to 6.388)	2.067 (0.656 to 6.510)
Self-rated health					
Very good/excellent					Reference
Good					1.153 (0.742 to 1.791)
Fair/poor					2.071 (1.147 to 3.739)*
HADS total score					0.972 (0.929 to 1.018)
Townsend disability					1.133 (1.044 to 1.229)**

**p* < .05, ***p* < .01, ****p* < .001.

REALM, Rapid Estimate of Adult Literacy in Medicine; IQ, Intelligence Quotient; HADS, Hospital Anxiety and Depression Scale.

For peer review only - http://bmjopen.bmj.com/site/about/guidelines.xhtml

Supplementary Table 7 Hazard ratios (95% confidence intervals) for the association between S-TOFHLA and mortality, controlling for sociodemographic, cognitive ability, and health variables. Models are run on a subsample of participants with all variables of interest (N = 680).

	Model 1<br>Age and sex	Model 2<br>+ age-11 IQ	Model 3<br>+ current fluid ability	Model 4<br>+ sociodemographics	Model 5<br>+ health status
S-TOFHLA	0.947 (0.917 to 0.978)**	0.949 (0.914 to 0.986)**	0.969 (0.929 to 1.009)	0.975 (0.934 to 1.018)	0.998 (0.953 to 1.046)
Age	0.924 (0.688 to 1.242)	0.925 (0.689 to 1.243)	0.909 (0.676 to 1.223)	0.919 (0.681 to 1.240)	0.936 (0.697 to 1.256)
Sex					
Female	Reference	Reference	Reference	Reference	Reference
Male	1.304 (0.893 to 1.902)	1.299 (0.889 to 1.897)	1.350 (0.925 to 1.972)	1.233 (0.814 to 1.866)	1.352 (0.881 to 2.074)
Age-11 IQ		0.998 (0.985 to 1.012)	1.008 (0.993 to 1.024)	1.007 (0.992 to 1.023)	1.006 (0.991 to 1.022)
Fluid ability			0.712 (0.557-0.910)**	0.717 (0.556 to 0.923)*	0.759 (0.587 to 0.982)*
Years of education				1.208 (0.994 to 1.469)	1.249 (1.026 to 1.520)*
Occupational class					
Professional				Reference	Reference
Managerial/technical				1.853 (0.935 to 3.670)	1.844 (0.931 to 3.650)
Skilled: non-manual				2.105 (0.992 to 4.464)	2.207 (1.042 to 4.673)*
Skilled: manual				3.038 (1.358 to 6.796)**	2.881 (1.275 to 6.509)*
Partly skilled/ unskilled manual				1.755 (0.556 to 5.541)	1.773 (0.562 to 5.598)
Self-rated health					
Very good/excellent					Reference
Good					1.147 (0.728 to 1.807)
Fair/poor					2.209 (1.216 to 4.014)**
HADS total score					0.974 (0.930 to 1.021)
Townsend disability					1.131 (1.039 to 1.232)**

*$p < .05$, **$p < .01$.

S-TOFHLA, Shortened Test of Functional Health Literacy in Adults; IQ, Intelligence Quotient; HADS, Hospital Anxiety and Depression Scale.

For peer review only - http://bmjopen.bmj.com/site/about/guidelines.xhtml

Supplementary Table 8 Hazard ratios (95% confidence intervals) for the association between NVS and mortality, controlling for sociodemographic, cognitive ability, and health variables. Models are run on a sub-sample of participants with all variables of interest (N = 724).

	Model 1<br>Age and sex	Model 2<br>+ age-11 IQ	Model 3<br>+ current fluid ability	Model 4<br>+ sociodemographics	Model 5<br>+ health status
NVS	0.880 (0.800 to 0.968)**	0.893 (0.803 to 0.993)*	0.961 (0.858 to 1.078)	0.960 (0.854 to 1.079)	0.961 (0.853 to 1.082)
Age	0.993 (0.756 to 1.306)	0.991 (0.754 to 1.303)	0.945 (0.714 to 1.250)	0.940 (0.709 to 1.248)	0.937 (0.708 to 1.242)
Sex					
Female	Reference	Reference	Reference	Reference	Reference
Male	1.346 (0.933 to 1.943)	1.329 (0.919 to 1.923)	1.371 (0.948 to 1.982)	1.228 (0.820 to 1.840)	1.355 (0.893 to 2.057)
Age-11 IQ		0.996 (0.983 to 1.009)	1.008 (0.993 to 1.022)	1.008 (0.993 to 1.023)	1.009 (0.994 to 1.023)
Fluid ability			0.651 (0.511 to 0.830)**	0.678 (0.529 to 0.869)**	0.748 (0.580 to 0.966)*
Years of education				1.212 (0.999 to 1.470)	1.242 (1.023 to 1.508)*
Occupational class					
Professional				Reference	Reference
Managerial/technical				2.211 (1.123 to 4.354)*	2.243 (1.140 to 4.414)*
Skilled: non-manual				2.435 (1.152 to 5.146)*	2.593 (1.231 to 5.463)*
Skilled: manual				3.590 (1.637 to 7.874)**	3.360 (1.522 to 7.415) **
Partly skilled/ unskilled manual				2.101 (0.668 to 6.604)	2.086 (0.661 to 6.578)
Self-rated health					
Very good/excellent					Reference
Good					1.175 (0.756 to 1.826)
Fair/poor					2.099 (1.167 to 3.775)*
HADS total score					0.973 (0.930 to 1.018)
Townsend disability					1.132 (1.044 to 1.228)**

*p < .05, **p < .01.

NVS, Newest Vital Sign; IQ, Intelligence Quotient; HADS, Hospital Anxiety and Depression Scale.

For peer review only

Supplementary Table 9 Hazard ratios (95% confidence intervals) for the association between general health literacy and mortality, controlling for sociodemographic, cognitive ability, and health variables. Models are run on a sub-sample of participants with all variables of interest (N = 677).

	Model 1<br>Age and sex	Model 2<br>+ age-11 IQ	Model 3<br>+ current fluid ability	Model 4<br>+ sociodemographics	Model 5<br>+ health status
General health literacy	0.769 (0.640 to 0.924)**	0.765 (0.609 to 0.961)*	0.894 (0.693 to 1.153)	0.903 (0.694 to 1.176)	0.950 (0.725 to 1.245)
Age	0.940 (0.701 to 1.260)	0.940 (0.701 to 1.260)	0.928 (0.690 to 1.247)	0.934 (0.692 to 1.260)	0.942 (0.700 to 1.266)
Sex					
Female	Reference	Reference	Reference	Reference	Reference
Male	1.264 (0.863 to 1.851)	1.265 (0.864 to 1.852)	1.330 (0.908 to 1.949)	1.205 (0.794 to 1.829)	1.337 (0.869 to 2.056)
Age-11 IQ		1.001 (0.986 to 1.015)	1.008 (0.992 to 1.024)	1.007 (0.991 to 1.023)	1.007 (0.992 to 1.023)
Fluid ability			0.698 (0.538 to 0.905)**	0.708 (0.543 to 0.922)*	0.770 (0.589 to 1.007)
Years of Education				1.229 (1.010 to 1.497)*	1.255 (1.030 to 1.528)*
Occupational class					
Professional				Reference	Reference
Managerial/technical				1.863 (0.941 to 3.689)	1.870 (0.945 to 3.700)
Skilled: non-manual				2.070 (0.976 to 4.390)	2.192 (1.035 to 4.640)*
Skilled: manual				3.072 (1.377 to 6.857)**	2.823 (1.252 to 6.365)*
Partly skilled/ unskilled manual				1.794 (0.570 to 5.649)	1.759 (0.557 to 5.561)
Self-rated health					
Very good/excellent					Reference
Good					1.152 (0.733 to 1.810)
Fair/poor					2.229 (1.229 to 4.042)*
HADS total score					0.975 (0.931 to 1.022)
Townsend disability					1.128 (1.040 to 1.225)**

*$p < .05$, **$p < .01$.

General health literacy, general measure of health literacy created by entering the REALM, S-TOFHLA and NVS into a PCA; IQ, Intelligence Quotient; HADS, Hospital Anxiety and Depression Scale.

For peer review only

For peer review only - http://bmjopen.bmj.com/site/about/guidelines.xhtml

STROBE 2007 (v4) Statement—Checklist of items that should be included in reports of *cohort studies*

Section/Topic	Item #	Recommendation	Reported on page #
**Title and abstract**	1	(*a*) Indicate the study's design with a commonly used term in the title or the abstract	1
		(*b*) Provide in the abstract an informative and balanced summary of what was done and what was found	2
**Introduction**			
Background/rationale	2	Explain the scientific background and rationale for the investigation being reported	4-5
Objectives	3	State specific objectives, including any prespecified hypotheses	6
**Methods**			
Study design	4	Present key elements of study design early in the paper	2, 6-11
Setting	5	Describe the setting, locations, and relevant dates, including periods of recruitment, exposure, follow-up, and data collection	6-10
Participants	6	(*a*) Give the eligibility criteria, and the sources and methods of selection of participants. Describe methods of follow-up	6-10
		(*b*) For matched studies, give matching criteria and number of exposed and unexposed	NA
Variables	7	Clearly define all outcomes, exposures, predictors, potential confounders, and effect modifiers. Give diagnostic criteria, if applicable	7-10
Data sources/ measurement	8*	For each variable of interest, give sources of data and details of methods of assessment (measurement). Describe comparability of assessment methods if there is more than one group	7-10
Bias	9	Describe any efforts to address potential sources of bias	NA
Study size	10	Explain how the study size was arrived at	6-7, 11
Quantitative variables	11	Explain how quantitative variables were handled in the analyses. If applicable, describe which groupings were chosen and why	7-10
Statistical methods	12	(*a*) Describe all statistical methods, including those used to control for confounding	10-11
		(*b*) Describe any methods used to examine subgroups and interactions	NA
		(*c*) Explain how missing data were addressed	6-7, 11
		(*d*) If applicable, explain how loss to follow-up was addressed	NA
		(*e*) Describe any sensitivity analyses	NA
**Results**			

For peer review only

Participants	13*	(a) Report numbers of individuals at each stage of study—eg numbers potentially eligible, examined for eligibility, confirmed eligible, included in the study, completing follow-up, and analysed	6-7, 11, 13-14, 17
		(b) Give reasons for non-participation at each stage	6-7, 11
		(c) Consider use of a flow diagram	NA
Descriptive data	14*	(a) Give characteristics of study participants (eg demographic, clinical, social) and information on exposures and potential confounders	11-14
		(b) Indicate number of participants with missing data for each variable of interest	13-14
		(c) Summarise follow-up time (eg, average and total amount)	13
Outcome data	15*	Report numbers of outcome events or summary measures over time	13
Main results	16	(*a*) Give unadjusted estimates and, if applicable, confounder-adjusted estimates and their precision (eg, 95% confidence interval). Make clear which confounders were adjusted for and why they were included	17
		(*b*) Report category boundaries when continuous variables were categorized	NA
		(*c*) If relevant, consider translating estimates of relative risk into absolute risk for a meaningful time period	NA
Other analyses	17	Report other analyses done—eg analyses of subgroups and interactions, and sensitivity analyses	15, supplementary materials
**Discussion**			
Key results	18	Summarise key results with reference to study objectives	18
**Limitations**			
Interpretation	20	Give a cautious overall interpretation of results considering objectives, limitations, multiplicity of analyses, results from similar studies, and other relevant evidence	18-20
Generalisability	21	Discuss the generalisability (external validity) of the study results	20
**Other information**			
Funding	22	Give the source of funding and the role of the funders for the present study and, if applicable, for the original study on which the present article is based	21

*Give information separately for cases and controls in case-control studies and, if applicable, for exposed and unexposed groups in cohort and cross-sectional studies.

Note: An Explanation and Elaboration article discusses each checklist item and gives methodological background and published examples of transparent reporting. The STROBE checklist is best used in conjunction with this article (freely available on the Web sites of PLoS Medicine at http://www.plosmedicine.org/, Annals of Internal Medicine at http://www.annals.org/, and Epidemiology at http://www.epidem.com/). Information on the STROBE Initiative is available at www.strobe-statement.org.

For peer review only

BMJ Open

The role of cognitive ability in the association between functional health literacy and mortality in the Lothian Birth Cohort 1936: a prospective cohort study

Journal:	*BMJ Open*
Manuscript ID	bmjopen-2018-022502.R1
Article Type:	Research
Date Submitted by the Author:	01-Jun-2018
Complete List of Authors:	Fawns-Ritchie, Chloe; University of Edinburgh, Centre for Cognitive Ageing and Cognitive Epidemiology<br>Starr, John; University of Edinburgh<br>Deary, Ian; Dept of Psychology
**Primary Subject Heading**:	Epidemiology
Secondary Subject Heading:	Public health
Keywords:	EPIDEMIOLOGY, PUBLIC HEALTH, STATISTICS & RESEARCH METHODS

SCHOLARONE™ Manuscripts

For peer review only

The role of cognitive ability in the association between functional health literacy and mortality in the Lothian Birth Cohort 1936: a prospective cohort study

Chloe Fawns-Ritchie[1,2], John M Starr[1,3], and Ian J Deary[1,2*]

[1]Centre for Cognitive Ageing and Cognitive Epidemiology, University of Edinburgh, Edinburgh, UK

[2]Department of Psychology, University of Edinburgh, Edinburgh, UK

[3]Alzheimer Scotland Dementia Research Centre, University of Edinburgh, Edinburgh, UK

*Corresponding author: Ian J Deary, Centre for Cognitive Ageing and Cognitive Epidemiology, Department of Psychology, University of Edinburgh

7 George Square

Edinburgh, EH8 9JZ

Scotland, UK

Telephone: +44 131 650 3452

i.deary@ed.ac.uk

Word count: 5200

For peer review only

ABSTRACT

Objectives We investigated the role that childhood and old age cognitive ability play in the association between functional health literacy and mortality.

Design Prospective cohort study.

Setting This study used data from the Lothian Birth Cohort 1936 study, which recruited participants living in the Lothian region of Scotland when aged 70 years, most of whom had completed an intelligence test at age 11.

Participants 795 members of the Lothian Birth Cohort 1936 with scores on tests of functional health literacy and cognitive ability in childhood and older adulthood.

Primary and secondary outcome measures Participants were followed up for 8 years to determine mortality. Time to death in days was used as the primary outcome measure.

Results Using Cox regression, higher functional health literacy was associated with lower risk of mortality adjusting for age and sex, using the Shortened Test of Functional Health Literacy in Adults (HR = 0.95, 95% CI 0.92 to 0.98), the Newest Vital Sign (HR = 0.88, 95% CI 0.80 to 0.97), and a functional health literacy composite measure (HR = 0.77, 95% CI 0.65 to 0.92), but not the Rapid Estimate of Adult Literacy in Medicine (HR = 0.95, 95% CI 0.90 to 1.01). Adjusting for childhood intelligence did not change these associations. When additionally adjusting for fluid-type cognitive ability in older age associations between functional health literacy and mortality were attenuated and non-significant.

Conclusions Current fluid ability but not childhood intelligence attenuated the association between functional health literacy and mortality. Functional health literacy measures may, in part, assess fluid-type cognitive abilities and this may account for the association between functional health literacy and mortality.

For peer review only - http://bmjopen.bmj.com/site/about/guidelines.xhtml

Strengths and limitations of this study

- This study had multiple tests of functional health literacy that assess different components of functional health literacy, which enabled us to create a composite functional health literacy measure.
- This study had comprehensive tests of cognitive ability measured in both childhood and old age which allowed us to investigate whether childhood and old age cognitive ability independently played a role in the relationship between health literacy and mortality.
- The health literacy measures used here only assessed functional health literacy and therefore we cannot determine whether cognitive ability would attenuate the association between health literacy and mortality if we used multi-dimensional health literacy measures.
- Larger samples and a longer follow-up time are needed to determine the role of cognitive ability in the association between functional health literacy and cause-specific mortality.

For peer review only

For peer review only - http://bmjopen.bmj.com/site/about/guidelines.xhtml

INTRODUCTION

Health literacy is "the degree to which individuals have the capacity to obtain, process and understand basic health information and services needed to make basic health decisions".[1] This ability is thought to be multifaceted and encompass the set of skills required to navigate the health-care environment.[2-4] One component of health literacy is functional health literacy—the reading, writing, and numeracy skills required to understand health information.[3, 5, 6] Tests designed to assess functional health literacy have been developed to measure health-related reading and numeracy skills, such as the commonly used Test of Functional Health Literacy in Adults.[5, 6] This test requires participants to read materials often used in the health-care setting, such as a medicine bottle, and answer questions about these materials.

Performance on functional health literacy tests have been associated with a range of health outcomes. Individuals with lower functional health literacy are more likely to require emergency care and have poorer skills in relation to correctly taking medication and interpreting written health materials.[7] Individuals with higher functional health literacy are more likely to take part in health-promoting behaviours such as eating a healthy diet, and are more likely to take part in routine cancer screening.[8, 9]

Successful completion of functional health literacy measures rely on cognitive functions, such as processing capacity and reasoning.[10, 11] One dominant theory in intelligence research is that there is a distinction between fluid ability, the ability to problem solve using novel material, which tends to decline with increasing age, and crystallised ability, which is the knowledge acquired throughout life which remains relatively stable across the lifespan.[12-16] Successful completion of tests of functional health literacy likely requires both crystallised abilities such as specific knowledge relating to health, and fluid abilities such as reasoning.[10, 11] It is therefore unsurpring that performance on tests of functional

For peer review only - http://bmjopen.bmj.com/site/about/guidelines.xhtml

health literacy and cognitive function are strongly related.[17-24] Some tests of functional health literacy have been found to correlate more strongly with measures of cognitive ability than with each other.[23, 25, 26] This overlap is so strong that some have proposed that functional health literacy should not be considered a unique construct but, instead, should be thought of as a specific component of cognitive function.[26]

Given the association between performance on tests of functional health literacy and cognitive ability tests, researchers have investigated whether the relationship between functional health literacy and health remain when also measuring cognitive ability. Whereas most evidence suggests that cognitive function explains a large proportion of the association between functional health literacy and health, the degree of attenuation varies.[25, 27, 28] A study using participants from the Lothian Birth Cohort 1936[25]—the same sample used in the current study—investigated whether cognitive ability in childhood and late adulthood attenuated the association between functional health literacy and physical health. In models without cognitive function, functional health literacy was associated with all three of the measures of physical health assessed. Addition of cognitive ability in older age significantly attenuated the association between functional health literacy with physical fitness by 43%, and number of natural teeth by 39%; however, it did not attenuate the association between functional health literacy and body mass index (BMI). Conversely, whereas childhood cognitive ability did not attenuate the association between functional health literacy and physical fitness, it attenuated the association between functional health literacy and number of teeth by 30%, and BMI by 88%. In the fully adjusted model which included childhood and late adulthood cognitive ability, as well as other early-life factors, the association between functional health literacy and physical fitness, though attenuated by 43%, remained significant,[25] suggesting that functional health literacy may play a small but unique role in physical fitness.

For peer review only

For peer review only - http://bmjopen.bmj.com/site/about/guidelines.xhtml

Mortality is arguably one of the most important health outcomes to examine. Both cognitive ability[29, 30] and functional health literacy[31] have been found to predict mortality. Researchers have therefore investigated the degree to which cognitive function explains the association between functional health literacy and mortality. When not controlling for cognitive function, Baker et al.[32] found that individuals with inadequate compared to adequate health literacy had a 50% higher risk of dying. When additionally adjusting for cognitive function, the risk reduced to 27%, but remained significant. A similar pattern of attenuation was found in another study.[33] Thus, cognitive function did not fully explain this relationship. These two studies, however, used brief measures of functional health literacy and cognitive function.

The present study sought to better understand the relationship between functional health literacy, cognitive ability and mortality using data from the LBC1936. We note that this is the same sample as used in Mõttus et al.[25] to investigate the association between functional health literacy, cognitive ability and physical health. In this previous study,[25] physical health was measured concurrently with fluid ability and functional health literacy. The current analysis is different from and complementary to this previous study in that we followed up the participants for 8 years to determine mortality status—obviously a most important health outcome. Studies that have examined the role that cognitive function plays in the association between functional health literacy and mortality used brief cognitive measures collected at the same time as the functional health literacy tests.[32, 33] It is not known whether early life cognitive ability and cognitive ability in older age play different roles in the association between health literacy and mortality. The current analysis utilises cognitive test scores collected in childhood, which are thought to measure the trait of lifelong intelligence, and current cognitive ability in older age, measured at approximately 73 years and contemporaneously with functional health literacy. The aim of this study was to

For peer review only - http://bmjopen.bmj.com/site/about/guidelines.xhtml

determine whether childhood cognitive ability and current cognitive ability in older adulthood play unique roles in the association between functional health literacy and mortality.

METHODS

Participants

LBC1936 is a cohort study of 1091 older adults born in 1936, most of whom reside in the Lothian area in Scotland. Most had taken part in the Scottish Mental Survey 1947, which tested the intelligence of almost all children born in 1936 and attending Scottish schools on 4th June 1947.[34] LBC1936 consists of a sample of these individuals who were subsequently followed-up, for the first time, at age 70 years (wave 1). To date, these participants have been followed-up a further three times at approximately 3 year intervals (waves 2-4). LBC1936 was designed principally to investigate healthy, non-pathological, cognitive ageing. Detailed information on this cohort is provided elsewhere.[35, 36] The present study used a sub-sample of 795 (413 male, 382 female) LBC1936 participants who completed tests of health literacy at wave 2 when participants were approximately aged 73 years. Figure 1 shows a flow chart of how the analytic sample for this current study was derived.

Ethical approval was obtained from the Scotland A Research Ethics Committee (07/MRE00/58). Written informed consent was obtained from participants. This study conformed to the principles embodied in the Declaration of Helsinki.

Measures

Mortality and survival time

For peer review only - http://bmjopen.bmj.com/site/about/guidelines.xhtml

The General Register Office for Scotland was used to identify deaths. Deaths through to end of March 2017 were recorded and this date is used as the censoring date for participants who survived. Survival time was measured in days from date of attending study visit at wave 2 to date of death or censoring date.

Functional health literacy

Three functional health literacy tests were administered at wave 2.

Rapid Estimate of Adult Literacy in Medicine (REALM):[37] This test measures participants' ability to read and correctly pronounce medical words. Participants are presented a piece of paper with a list of 66 medical words and are asked to read these words aloud. The words range in difficulty from easy ("fat") to difficult ("impetigo"). One point is given for each correctly pronounced word. One week test-retest (r = 0.99)[37] and internal consistency (Cronbach's alpha = 0.98)[38] have been found to be very high.

Shortened Test of Functional Health Literacy in Adults (S-TOFHLA):[5, 6]. In the numeracy section, participants are provided with cards with medical information on them and are asked four questions about this information. The reading comprehension section comprised a 36-item task which involved participants reading two health-related passages where every fifth to seventh word was missing and participants were to select the missing word from four options. Participants had 12 minutes to complete both sections. Here, the British version of the S-TOFHLA[9] was used which substitutes the Medicaid passage for a passage about UK prescription fee exemptions. This measure is a shortened version of the Test of Functional Health Literacy in Adults, which is seen as the gold standard functional health literacy test[39]. Successful completion of the S-TOFHLA requires the ability to read and comprehend written words and numbers in a health context. Internal consistency is high for

For peer review only - http://bmjopen.bmj.com/site/about/guidelines.xhtml

reading comprehension (Cronbach's alpha = 0.97)[6] and adequate for numeracy (Cronbach's alpha = 0.68).[6] The S-TOFHLA has been found to correlate strongly with the REALM (r = 0.80).[6]

Newest Vital Sign (NVS):[40] Participants were presented with a nutrition label from a container of ice cream and were asked to answer six questions about the information provided on this label. The NVS assesses both reading comprehension and numeracy skills associated with health as participants need to use the written text and numbers on the label to answer the questions.[40] The NVS correlates with the S-TOFHLA at r = 0.59[40] and shows reasonable internal consistency (Cronbach's alpha = 0.76).[40]

General health literacy: The three functional health literacy measures used here have been found to correlate moderately with each other.[25] To capture the shared variance between these tests, a general measure of functional health literacy was created by entering scores on the three tests into a principal component analysis (PCA). Two of these measures had skewed distributions (see Supplementary Figures 1-8), therefore Spearman's rank correlation was used in the PCA. Only the first component had an eigenvalue greater than 1, and the scree slope indicated a single component; therefore scores from the first unrotated principal component were used as a composite of functional health literacy (general functional health literacy). This component accounted for 59.7% of the total variance, confirming there was substantial shared variance between the three functional health literacy tests. The REALM, S-TOFHLA and NVS loaded 0.74, 0.80, and 0.77, respectively, on this component.

Cognitive ability

Childhood cognitive ability (age-11 IQ): As part of the Scottish Mental Survey 1947, almost all 11 year old children in Scotland in 1947 sat the Moray House Test No. 12 (MHT);[34] a

For peer review only - http://bmjopen.bmj.com/site/about/guidelines.xhtml

45-minute, group-administered intelligence test that included tasks of verbal reasoning and spatial ability, and had a maximum score of 76. In LBC1936, scores on the MHT were adjusted for age in days at testing and then were converted into standard IQ-type scores with a mean of 100 and a standard deviation of 15. This score will be used as a measure of prior, or crystallised, ability.

Current fluid ability: Participants completed a lengthy cognitive assessment.[35, 36] As has been done in previous LBC1936 studies,[23, 25] six tests administered at wave 2 thought to measure fluid-type cognitive abilities that tend decline across the lifespan[14-16] were entered into a PCA. The following tests from the Wechsler Adult Intelligence Scale-III[41] that assess non-verbal reasoning, visuospatial ability, working memory, and processing speed were used: Matrix Reasoning, Block Design, Letter-Number Sequencing, Symbol Search, Digit Span Backwards, and Digit Symbol-coding. Only the first component had an eigenvalue greater than 1 and the scree slope indicated one component, and therefore scores from this first principal component were used as a measure of current fluid ability. This component accounted 50.2% of the total variance. The loadings for the six tests were: Matrix Reasoning = 0.69; Block Design = 0.71; Letter-Number Sequencing = 0.71; Symbol Search = 0.75; Digit Span Backwards = 0.64; Digit Symbol-coding = 0.75.

Covariates

Sociodemographic variables included in this analysis were education and occupational social class. Years of full-time education completed, recorded at wave 1 when participants were aged 70 years, was used to measure education. At wave 1, participants were assigned to one of the following occupational social classes based on their highest occupational status prior to retirement:[42] professional, managerial and technical, skilled, partly skilled manual,

For peer review only - http://bmjopen.bmj.com/site/about/guidelines.xhtml

unskilled manual. Female participants were assigned the occupational class of their husband if this was higher than their own. Skilled was separated into skilled non-manual and skilled manual. Only 5 participants in this sample were assigned the occupational class of unskilled, therefore partly skilled manual, and unskilled manual were combined into one class, hereafter referred to as manual (N = 31).

Three measures of health status measured at wave 2 were used. Self-reported health was measured by asking participants whether they rated their general health to be excellent, very good, good, fair or poor. Only a small number of participants who were recorded dead at the censoring date reported poor (N = 3) or excellent (N = 17) health. Therefore, poor and fair were collapsed into one category (fair/poor; N = 73), as were very good and excellent (very good/excellent; N = 487). Total score on the Hospital Anxiety and Depression Scale (HADS)[43] was used as a measure of mood state. Higher scores on the HADS represent higher levels of anxiety and depression. Activities of daily living were assessed using the Townsend Disability Scale.[44] Participants were given a score of 0 (no difficulty completing this activity) to 2 (not able complete this activity) for nine activities, and thus higher scores represent more functional disability.

Patient and public involvement

LBC1936 participants were not involved in the development of any part of this study. The results will be disseminated to participants via a quarterly newsletter sent to LBC1936 participants.

For peer review only - http://bmjopen.bmj.com/site/about/guidelines.xhtml

Statistical analysis

SPSS version 21.0 was used to carry out this analysis. To determine whether those recorded as alive or dead at censoring date differ on demographic, functional health literacy, cognitive function, or health status variables, chi-square tests were conducted for categorical variables, independent t-tests were used for normally-distributed continuous variables, and Mann-Whitney U tests were used for non-normal continuous variables. Spearman's rank-order correlation was used to examine the relationship between functional health literacy and cognitive ability scores. To investigate the association between functional health literacy and time to death, Cox proportional hazard regression was used. For each of the functional health literacy measures of interest (REALM, S-TOFHLA, NVS, and the component score of general functional health literacy) six models were run. In Model 1 the functional health literacy measure of interest and age and sex were entered. Years of education was added in Model 2 as this has been found to be associated with functional health literacy. To determine whether cognitive ability in childhood attenuated the association between functional health literacy and mortality, age-11 IQ was added (Model 3). In Model 4, fluid-type cognitive ability in older age was additionally added to determine its role in the association between functional health literacy and mortality. Occupational class was additionally included in Model 5. Finally, health status variables (self-reported health, HADS, and Townsend) were included in Model 6. Methods to control for multiple testing were not used here. We were interested in the change in the effect size of the association between functional health literacy and mortality following the inclusion of various cognitive, sociodemographic and health variables. In the results section of the main text here, only the hazard ratios (HRs) and 95% confidence intervals (CIs) for the functional health literacy measures are reported. A more detailed description of the results for all variables in the models is given in the supplementary materials.

For peer review only

For peer review only - http://bmjopen.bmj.com/site/about/guidelines.xhtml

RESULTS

A total of 796 participants completed the functional health literacy measures at wave 2 (Figure 1). Following removal of one participant without information on date of death, 130 participants had died, and 665 participants were alive at the censoring date. Participant characteristics are reported in Table 1 and functional health literacy and cognitive ability scores are shown in Table 2. Those who died were more likely to be from a lower occupational class, were more likely to report poorer health, and reported more disability than those who survived. Participants who had died had lower scores on all the functional health literacy measures, and had lower fluid cognitive ability scores in older age. Age-11 IQ did not differ between the two groups.

Table 3 shows the rank order correlations between functional health literacy and cognitive ability measures. These have been reported elsewhere.[23, 25] The three functional health literacy measures correlated moderately with each other ($r = 0.35\text{-}0.44$, $p < .001$), and higher scores on the functional health literacy measures were correlated with higher age-11 IQ ($r = 0.44\text{-}0.51$, $p < .001$), and higher fluid ability ($r = 0.38\text{-}0.55$, $p < .001$). The three functional health literacy measures tended to correlate more strongly with measures of cognitive ability than with each other. The general functional health literacy measure also showed a strong positive correlation with both age-11 IQ ($r = 0.61$, $p < .001$) and fluid ability in older age ($r = 0.63$, $p < .001$). The correlations between all variables examined in this analysis are reported in Supplementary Table 1.

For peer review only - http://bmjopen.bmj.com/site/about/guidelines.xhtml

Table 1 Participant characteristics for participants alive or dead at censoring date and *p*-values to determine whether these characteristics differed by survival status

	N	Alive	Dead	*p*-value
Survival time (years), mean (SD)	795	8.19 (0.66)	5.23 (2.14)	
Age (years) at wave 2, mean (SD)	795	72.54 (0.70)	72.41 (0.72)	.068
Sex, n (%)	795			.069
Male		336 (50.5)	77 (59.2)	
Female		329 (49.5)	53 (40.8)	
Years of education, mean (SD)	795	10.80 (1.16)	10.71 (1.10)	.417
Occupational class, n (%)	780			.001
Professional		142 (21.7)	12 (9.4)	
Managerial/technical		249 (38.1)	49 (38.6)	
Skilled: non-manual		140 (21.4)	26 (20.5)	
Skilled: manual		96 (14.7)	35 (27.6)	
Manual		26 (4.0)	5 (3.9)	
Self-reported health, n (%)	795			<.001
Poor/fair		47 (7.1)	26 (19.9)	
Good		195 (29.4)	40 (30.5)	
Very good/excellent		422 (63.5)	65 (49.6)	
HADS total, mean (SD)	794	7.02 (4.37)	7.42 (4.62)	.342
Townsend disability, mean (SD)	794	0.89 (1.82)	1.60 (2.48)	.001

HADS, Hospital Anxiety and Depression Scale.

For peer review only

For peer review only - http://bmjopen.bmj.com/site/about/guidelines.xhtml

Table 2 Mean scores (SD) on measures of functional health literacy and cognitive ability by survival status, and *p*-values to determine whether these scores differ by survival status

	N	Alive	Dead	*p*-value
REALM score	794	65.08 (2.39)	64.67 (3.02)	.015
S-TOFHLA score	744	38.00 (3.85)	36.69 (5.37)	.025
NVS score	789	2.92 (1.90)	2.48 (1.92)	.011
General functional health literacy	740	0.05 (0.98)	-0.24 (1.08)	.007
Age-11 IQ	752	101.08 (14.99)	98.55 (16.33)	.091
Current fluid ability	789	0.07 (0.99)	-0.34 (1.00)	<.001

REALM, Rapid Estimate of Adult Literacy in Medicine; S-TOFHLA, Shortened Test of Functional Health Literacy in Adults; NVS, Newest Vital Sign; IQ, Intelligence Quotient.

The HRs for the association between functional health literacy and mortality are shown in Table 4. HRs for all variables entered into the models are reported in Supplementary Tables 2-5. In all models, the assumptions of proportional hazards were met. Given the high correlations between functional health literacy and cognitive ability, variance inflation factors (VIF) were calculated to check for multicollinearity. VIF values for all models were low (highest VIF = 2.15), suggesting there was no multicollinearity in these models.

REALM: The HRs for the REALM represent the risk of dying for a one point increase in the REALM (max score = 66). The REALM did not significantly predict mortality in Model 1 (HR = 0.95, 95% CI 0.90 to 1.01) adjusting for age and sex, or subsequently with the addition of education (Model 2), age-11 IQ (Model 3), fluid ability (Model 4), occupational class (Model 5), or health status (Model 6).

For peer review only - http://bmjopen.bmj.com/site/about/guidelines.xhtml

Table 3 Rank order correlations between functional health literacy and cognitive ability measures

	1	2	3	4	5	6
1 REALM	-					
2 S-TOFHLA	0.40*	-				
3 NVS	0.35*	0.44*	-			
4 General functional health literacy	0.71*	0.80*	0.78*	-		
5 Age-11 IQ	0.44*	0.48*	0.51*	0.61*	-	
6 Current fluid ability	0.38*	0.55*	0.55*	0.63*	0.57*	-

*$p < .001$.

REALM, Rapid Estimate of Adult Literacy in Medicine; S-TOFHLA, Shortened Test of Functional Health Literacy in Adults; NVS, Newest Vital Sign; IQ, Intelligence Quotient.

S-TOFHLA: The HRs for the S-TOFHLA represent the risk of mortality for a one point increase in S-TOFHLA score (max score = 40). With age and sex controlled for, a one-point increase in S-TOFHLA reduced the risk of dying by 5% (Model 1 HR = 0.95, 95% CI 0.92 to 0.98). Inclusion of education (Model 2) and age-11 IQ (Model 3) did not attenuate this association. This association was attenuated and became non-significant in Model 4 with the inclusion of fluid ability (HR = 0.97, 95% CI 0.93 to 1.01), and remained non-significant and continued to reduce in size following the addition of occupational class (Model 5) and health status (Model 6).

NVS: The HRs for NVS represent the risk of mortality for a one point increase in NVS score (max score = 6). In Model 1, in which age and sex were entered as covariates, NVS significantly predicted mortality. A one point increase in NVS score reduced the risk of dying by 12% (HR = 0.88, 95% CI 0.80 to 0.97). The addition of years of education (Model 2) did

For peer review only - http://bmjopen.bmj.com/site/about/guidelines.xhtml

not attenuate this association. Age-11 IQ was added in Model 3 and this did little to change the association between NVS and mortality. The inclusion of fluid ability in Model 4 greatly attenuated the association between NVS and mortality, and this association became non-significant (HR = 0.96, 95% CI 0.86 to 1.08). This association remained non-significant following the inclusion of occupational class (Model 5) and health status variables (Model 6).

General functional health literacy: The HRs for general functional health literacy represent the risk of mortality for a one SD increase in general functional health literacy. General functional health literacy predicted mortality in Model 1, controlling for age and sex. A one SD increase in general functional health literacy reduced the risk of mortality by 23% (HR = 0.77, 95% CI 0.65 to 0.92). Including years of education in Model 2 and age-11 IQ in Model 3 did little to change the association between general functional health literacy and mortality. Current fluid ability was included in Model 4 and this attenuated the association between general functional health literacy and mortality and this association was no longer significant (HR = 0.87, 95% CI 0.67 to 1.13). Adding occupational social class in Model 5 did little to change the association between general functional health literacy and mortality. Health status variables were added in Model 6 and the association between general functional health literacy and mortality was further attenuated and remained non-significant.

All models were re-run using only participants who had complete data on all of the variables of interest. These models are shown in Supplementary Tables 6-9. The associations between functional health literacy and mortality were similar to those reported here, except that, in Model 1 for the REALM (Supplementary Table 6), higher scores on the REALM significantly reduced the risk of mortality. This association was no longer significant in Model 2, following the inclusion of age-11 IQ.

For peer review only - http://bmjopen.bmj.com/site/about/guidelines.xhtml

Table 4 Hazard ratios (95% confidence intervals) for the association between four measures of functional health literacy and mortality, controlling for sociodemographic, cognitive, and health variables

	Model 1 Age and sex	Model 2 + education	Model 3 + age-11 IQ	Model 4 + current fluid ability in older age	Model 5 + occup class	Model 6 + health status
REALM	0.95 (0.90 to 1.01)	0.96 (0.90 to 1.01)	0.96 (0.90 to 1.02)	0.97 (0.91 to 1.04)	0.97 (0.90 to 1.04)	1.00 (0.92 to 1.07)
	N = 794	N = 794	N = 752	N = 746	N = 731	N = 728
S-TOFHLA	0.95 (0.92 to 0.98)**	0.95 (0.92 to 0.98)**	0.95 (0.91 to 0.98)**	0.97 (0.93 to 1.01)	0.98 (0.94 to 1.02)	1.00 (0.95 to 1.05)
	N = 744	N = 744	N = 702	N = 697	N = 682	N = 680
NVS	0.88 (0.80 to 0.97)**	0.88 (0.80 to 0.97)*	0.89 (0.80 to 0.99)*	0.96 (0.86 to 1.08)	0.97 (0.86 to 1.09)	0.96 (0.85 to 1.08)
	N = 789	N = 789	N = 746	N = 742	N = 727	N = 724
General functional	0.77 (0.65 to 0.92)**	0.75 (0.61 to 0.90)**	0.74 (0.59 to 0.93)*	0.87 (0.67 to 1.13)	0.911 (0.70 to 1.19)	0.95 (0.72 to 1.25)
health literacy	N = 740	N = 740	N = 698	N = 694	N = 679	N = 677

*$p < .05$, **$p < .01$

IQ, intelligence quotient; occup class, occupational class; REALM, Rapid Estimate of Adult Literacy in Medicine; S-TOFHLA, Shortened Test of Functional Health Literacy in Adults; NVS, Newest Vital Sign.

For peer review only - http://bmjopen.bmj.com/site/about/guidelines.xhtml

Sensitivity analyses: Participants who may have a dementia or possible pathological cognitive impairment were not removed prior to running these analyses. One participant self-reported a diagnosis of dementia at the wave 2 assessment. Five participants in this sample have mini-mental state exam scores below the often-used cutoff of 24[45] (one participant scored 20/30, one scored 22/30 and three scored 23/30), which suggests a possible cognitive impairment. To determine whether the presence of dementia or possible cognitive impairment affects the results, these analyses were re-run excluding these 6 individuals. All associations were very similar to those reported above (results not shown; available from the authors) and therefore the presence of dementia or possible cognitive impairment did not affect the main results.

DISCUSSION

This study investigated whether prior cognitive ability measured in childhood and current fluid cognitive ability measured in older adulthood played different roles in the association between functional health literacy and mortality. Four measures of functional health literacy were used; the REALM, S-TOFHLA, NVS, and a composite measure of functional health literacy. The REALM, a test that requires only the ability to read and correctly pronounce medical words, did not predict mortality, even in minimally adjusted models (though it had a slightly stronger and significant association when only those with full data were included, as shown in supplementary analysis). When using functional health literacy tests that assessed reading comprehension and numeracy (S-TOFHLA, NVS, and general functional health literacy), functional health literacy predicted mortality in models adjusting for age, sex and education only. Individuals who had higher scores on the S-TOFHLA, NVS, and general functional health literacy had a lower risk of mortality than those with lower scores. Accounting for prior intelligence measured in childhood did not change this association. The

For peer review only - http://bmjopen.bmj.com/site/about/guidelines.xhtml

association between functional health literacy and mortality disappeared when contemporaneous fluid ability was accounted for. The attenuation was particularly large for the NVS and general functional health literacy.

Two previous studies used functional health literacy tests that measure reading comprehension and numeracy to investigate the role that cognitive function plays in the association between functional health literacy and mortality.[32, 33] These studies measured cognitive function concurrently with health literacy in middle-age or older adulthood and found that, although the size of the association between functional health literacy and mortality was reduced, functional health literacy still predicted mortality when cognitive function was controlled for.[32, 33] We investigated the role that both childhood cognitive ability, and cognitive ability in older age have on the association between functional health literacy and mortality. Here, fluid ability, but not childhood intelligence, attenuated the association between functional health literacy and mortality such that the association was no longer significant. Childhood cognitive ability, which was measured decades prior to the functional health literacy assessment, is thought to reflect the relatively stable trait of lifelong intelligence, whereas current fluid ability, which was measured when participants were approximately 73 years old, is a measure of current cognitive competence.[23] These results suggest that, whereas childhood intelligence did not play a role in the association between functional health literacy and mortality, current fluid-type cognitive ability in older adulthood accounted for a large proportion of this association.

A strength of this current study is that detailed measures of cognitive ability were used. Childhood intelligence was measured using a standardised test of intelligence which had good concurrent validity with other intelligence tests.[35] The fluid ability measure comprised many standardised neuropsychological tests. Both Baker et al.[32] and Bostock and Steptoe[33] used brief measures of cognitive function. Baker et al.[32] used specific

For peer review only - http://bmjopen.bmj.com/site/about/guidelines.xhtml

items from the mini-mental state exam, a measure designed to screen for cognitive impairment[45] which is insensitive to individual differences in healthy cognitive ageing. Bostock and Steptoe[33] used three brief cognitive tests administered in a non-standardised way in the participants' own home. These studies may not have used tests sensitive enough, or that covered a necessary range of cognitive functions, to fully account for the association between health literacy and mortality.

Another advantage of the current study is the use of three different tests of functional health literacy. All tests were used to measure functional health literacy; however, each test required the participant to carry out different health-related tasks. Whereas the REALM required the participant only to read and correctly pronounce words, the S-TOFHLA and NVS are more cognitively demanding tasks that assessed both reading comprehension and numeracy skills. Using these three measures enabled us to investigate whether different patterns of association between functional health literacy and mortality were found when using the different tests. By using three measures of functional health literacy, we were also able to create a composite measure of functional health literacy. This general measure was derived with the aim of creating a score that captures the shared variance between the three functional health literacy tests, providing a more comprehensive measure of functional health literacy.

The results of this study support the proposal by Reeve and Basalik[26] that functional health literacy may not be a unique construct; instead, it is tenable that tests of functional health literacy may in fact be measuring cognitive ability. Here, NVS, S-TOFHLA and general functional health literacy no longer predicted mortality when accounting for fluid ability. This attenuation is likely to be because there is an overlap in the content of tests of fluid ability and the NVS and S-TOFHLA. The NVS and S-TOFHLA are cognitively demanding tasks that are likely to be substantially measuring fluid-type cognitive abilities, such as working memory and reasoning, that decline with increasing age.[15] Childhood cognitive

For peer review only - http://bmjopen.bmj.com/site/about/guidelines.xhtml

ability did not attenuate the association between functional health literacy, suggesting that the NVS and S-TOFHLA are measuring current fluid-type cognitive capability in old age, and not lifelong intelligence. Current fluid ability in old age may be driving the association between functional health literacy and mortality simply because tests of functional health literacy are assessing the same underlying abilities as measures of fluid ability.

Some researchers have questioned the validity of some of the functional health literacy tests used here. The Test of Functional Health Literacy in Adults is often reported as the gold standard functional health literacy test.[39] However, the NVS has been found to have poor concurrent validity with the Test of Functional Health Literacy in Adults.[39] In support of this, we found that the rank-order correlation between the NVS and S-TOFHLA was modest (r = 0.44). Concerns have been raised about the fact that the REALM assesses only the ability to read and pronounce words.[38] Knowing how to pronounce medical words may not be directly related to the ability to understand medical information, and therefore this may not adequately cover all the domains of functional health literacy.[38] Indeed, all the tests used here were designed to largely measure the component of health literacy known as functional health literacy. None of these measures assess other components of health literacy such as the skills required to critically analyse health information or the communicative skills needed to participate and navigate in the health-care environment.[3] Assessments of health literacy that are designed to measure a much broader range of health literacy skills are available. The European Health Literacy Survey Questionnaire measures self-reported skills in being able to access, understand, appraise, and apply health-related information in the health-care setting, as well as in disease prevention and health promotion.[46] Fluid cognitive ability may not play a role in the association between health literacy and mortality if we used these self-reported, broad, measures of health literacy, rather than the objective, but narrow, functional health literacy tests used here.

For peer review only - http://bmjopen.bmj.com/site/about/guidelines.xhtml

There are some limitations to this study. The LBC1936 participants were followed-up for the first time at age 70 years and therefore the sample used in this analysis will likely suffer from a survival bias as this sample is made up of individuals who have survived to the age of 70 years. LBC1936 participants also tended to have higher scores on the Moray House Test (age-11 IQ test) than Scottish-wide and Edinburgh-wide participants who also sat this test in 1947 as part of the Scottish Mental Survey.[36] Thus, individuals in this sample tended to be brighter than the original Scottish Mental Survey 1947 participants. This analysis only examined the association between functional health literacy and all-cause mortality. It is possible that there are different relationships between functional health literacy and cause-specific mortality, for example functional health literacy may only predict deaths linked to unhealthy lifestyles, such as cardiovascular disease. The follow-up period in this study was relatively short, and therefore only a small percentage of participants had died. Future studies should investigate mortality over a longer follow-up period and in larger samples to examine whether there are different patterns of association between functional health literacy and cause-specific mortality.

We investigated whether childhood cognitive ability and fluid ability in older age play independent roles in the association between functional health literacy and mortality. The results indicate that fluid-type cognitive capability may account for the association between functional health literacy and mortality, while childhood cognitive ability—an indicator of lifelong intelligence—does not. Researchers and clinicians should be aware that lower functional health literacy scores may actually reflect lower cognitive ability in older age, and that current cognitive capacity in older adulthood, but not lifelong intelligence, may be driving the association between functional health literacy and mortality. Future research examining the association between functional health literacy and mortality, and other health

For peer review only - http://bmjopen.bmj.com/site/about/guidelines.xhtml

indicators, should also include measures of cognitive ability to be able to properly disentangle the relationship between functional health literacy and health.

For peer review only

For peer review only - http://bmjopen.bmj.com/site/about/guidelines.xhtml

Contributors CFR discussed and planned the study and analyses, analysed the data, interpreted the data and drafted the initial manuscript. JMS discussed and planned the study and analyses, interpreted the data and contributed to the manuscript. IJD discussed and planned the study and analyses, interpreted the data and contributed to the manuscript.

Funding This work was supported by the University of Edinburgh Centre for Cognitive Ageing and Cognitive Epidemiology, part of the cross council Lifelong Health and Wellbeing Initiative, funded by the Biotechnology and Biological Sciences Research Council (BBSRC), and Medical Research Council (MRC) (grant number MR/K026992/1). The Lothian Birth Cohort 1936 (LBC1936) is supported by Age UK (Disconnected Mind project).

Completing interests None declared.

Ethical approval Ethical approval was obtained from the Scotland A Research Ethics Committee (07/MRE00/58).

Data sharing statement Lothian Birth Cohort 1936 data can be requested from the Lothian Birth Cohort 1936 research team, following completion of a data request application. More information can be found at: http://www.lothianbirthcohort.ed.ac.uk/content/collaboration

Acknowledgements We thank the LBC1936 participants and LBC1936 research team members.

For peer review only

REFERENCES

1 Institute of Medicine. Health Literacy: A Prescription to End Confusion. Washinton, DC: The National Academies Press, 2004.

2 Baker DW. The meaning and the measure of health literacy. *J Gen Intern Med* 2006;21(8):878-83. doi:10.1111/j.1525-1497.2006.00540.x

3 Nutbeam D. Health literacy as a public health goal: a challenge for contemporary health education and communication strategies into the 21st century. *Health Promotion International 2000*;15(3):259-67.

4 Nutbeam D. The evolving concept of health literacy. *Soc Sci Med* 2008;67(12):2072-78. doi:10.1016/j.socscimed.2008.09.050 [published Online First: 25 October 2008].

5 Parker RM, Baker DW, Williams MV, et al. The test of functional health literacy in adults. *J Gen Intern Med* 1995;10(10):537-41.

6 Baker DW, Williams MV, Parker RM, et al. Development of a brief test to measure functional health literacy. *Patient Educ Couns* 1999;38(1):33-42.

7 Berkman ND, Sheridan SL, Donahue KE, et al. Low health literacy and health outcomes: an updated systematic review. *Ann Intern Med* 2011;155(2):97-107. doi:10.7326/0003-4819-155-2-201107190-00005 [published Online First: 19 July 2011].

8 Kobayashi LC, Wardle J, von Wagner C. Limited health literacy is a barrier to colorectal cancer screening in England: evidence from the English Longitudinal Study of Ageing. *Prev Med* 2014;61:100-105. doi:10.1016/j.ypmed.2013.11.012 [published Online First: 25 November 2013].

9 Wagner CV, Knight K, Steptoe A, et al. Functional health literacy and health-promoting behaviour in a national sample of British adults. *J Epidemiol Community Health* 2007;61(12):1086-90. doi:10.1136/jech.2006.053967.

For peer review only - http://bmjopen.bmj.com/site/about/guidelines.xhtml

10 Chin J, Morrow DG, Stine-Morrow EA, et al. The process-knowledge model of health literacy: evidence from a componential analysis of two commonly used measures. *J Health Commun* 2011;16 Suppl 3:222-41. doi:10.1080/10810730.2011.604702 [published Online First: 27 September 2011].

11 Morrow D, Chin J. Decision Making and Health Literacy among Older Adults. In: Strough TMGN, Löckenhoff C, eds. Aging and Decision Making: Empirical and Applied Perspectives. London: Academic Press, 2015:261-82.

12 Cattell RB. Theory of fluid and crystallized intelligence: A critical experiment. *J Educ Psychol* 1963;54(1):1-22. doi:10.1037/h0046743.

13 Horn JL. A basis for research on age differences in cognitive capabilities. In: McArdle JJ, Woodcock RW, eds. Human cognitive abilities in theory and practice. New York: Routledge, 1998:57-91.

14 Park DC, Reuter-Lorenz P. The adaptive brain: aging and neurocognitive scaffolding. *Annu Rev Psychol* 2009;60:173-96. doi:10.1146/annurev.psych.59.103006.093656.

15 Salthouse TA. Selective review of cognitive aging. *J Int Neuropsychol Soc* 2010;16(5):754-60. doi:10.1017/s1355617710000706.

16 Schaie KW. Intellectual development in adulthood: The Seattle longitudinal study. Cambridge: Cambridge University Press, 1996.

17 Bennett JS, Boyle PA, James BD, et al. Correlates of health and financial literacy in older adults without dementia. *BMC Geriatr* 2012;12:30. doi:10.1186/1471-2318-12-30 [published Online First: 12 June 2012].

18 Dahlke AR, Curtis LM, Federman AD, et al. The mini mental status exam as a surrogate measure of health literacy. *J Gen Intern Med* 2014;29(4):615-20. doi:10.1007/s11606-013-2712-x [published Online First: 11 Dec 2013].

For peer review only

For peer review only - http://bmjopen.bmj.com/site/about/guidelines.xhtml

19 Federman AD, Sano M, Wolf MS, et al. Health literacy and cognitive performance in older adults. *J Am Geritar Soc* 2009;57(8):1475-80. doi:10.1111/j.1532-5415.2009.02347.x [published Online First: 8 June 2009].

20 Kobayashi LC, Wardle J, Wolf MS, et al. Cognitive Function and Health Literacy Decline in a Cohort of Aging English Adults. *J Gen Intern Med* 2015;30(7):958-64. doi:10.1007/s11606-015-3206-9 [published Online First: 14 February 2015].

21 Kobayashi LC, Smith SG, O'Conor R, et al. The role of cognitive function in the relationship between age and health literacy: a cross-sectional analysis of older adults in Chicago, USA. *BMJ Open* 2015;5(4):e007222. doi:10.1136/bmjopen-2014-007222 [published Online First: 23 April 2015].

22 Levinthal BR, Morrow DG, Tu W, et al. Cognition and health literacy in patients with hypertension. *J Gen Intern Med* 2008;23(8):1172-6. doi:10.1007/s11606-008-0612-2 [published Online First:6 May 2008].

23 Murray C, Johnson W, Wolf MS, et al. The Association between Cognitive Ability across the Lifespan and Health Literacy in Old Age: The Lothian Birth Cohort 1936. *Intelligence* 2011;39(4):178-87. doi:10.1016/j.intell.2011.04.001 [published Online First: 4 May 2011].

24 Ownby RL, Acevedo A, Waldrop-Valverde D, et al. Abilities, skills and knowledge in measures of health literacy. *Patient Educ Couns* 2014;95(2):211-7. doi:10.1016/j.pec.2014.02.002 [published Online First: 16 February 2014].

25 Mõttus R, Johnson W, Murray C, et al. Towards understanding the links between health literacy and physical health. *Health Psychol* 2014;33(2):164-73. doi:10.1037/a0031439.

26 Reeve CL, Basalik D. Is health literacy an example of construct proliferation? A conceptual and empirical evaluation of its redundancy with general cognitive ability.

For peer review only - http://bmjopen.bmj.com/site/about/guidelines.xhtml

Intelligence 2014;44:93-102. doi:10.1016/j.intell.2014.03.004[published Online First: 16 April 2014].

27 O'Conor R, Wolf MS, Smith SG, et al. Health literacy, cognitive function, proper use, and adherence to inhaled asthma controller medications among older adults with asthma. *Chest* 2015;147(5):1307-15. doi:10.1378/chest.14-0914 [published Online First: 2 October 2014].

28 Serper M, Patzer RE, Curtis LM, et al. Health Literacy, Cognitive Ability, and Functional Health Status among Older Adults. *Health Serv Res* 2014;49(4):1249-67. doi:10.1111/1475-6773.12154 [published Online First: 30 Jan 2014].

29 Murray C, Pattie A, Starr JM, et al. Does Cognitive Ability Predict Mortality in the Ninth Decade? The Lothian Birth Cohort 1921. *Intelligence* 2012;40(5):490-98. doi:10.1016/j.intell.2012.05.003 [published Online First: 12 June 2012].

30 Pavlik VN, de Moraes SA, Szklo M, et al. Relation between cognitive function and mortality in middle-aged adults: the atherosclerosis risk in communities study. *Am J Epidemiol* 2003;157(4):327-34. doi:10.1093/aje/kwf209.

31 Baker DW, Wolf MS, Feinglass J, et al. Health literacy and mortality among elderly persons. *Arch Intern Med* 2007;167(14):1503-9. doi:10.1001/archinte.167.14.1503.

32 Baker DW, Wolf MS, Feinglass J, et al. Health literacy, cognitive abilities, and mortality among elderly persons. *J Gen Intern Med* 2008;23(6):723-6. doi:10.1007/s11606-008-0566-4 [published Online First: 11 March 2008].

33 Bostock S, Steptoe A. Association between low functional health literacy and mortality in older adults: longitudinal cohort study. *BMJ* 2012;344:e1602. doi:10.1136/bmj.e1602[published Online First: 15 March 2012].

For peer review only

For peer review only - http://bmjopen.bmj.com/site/about/guidelines.xhtml

34 Scottish Council for Research in Education. The trend of Scottish intelligence: A comparison of the 1947 and 1932 surveys of intelligence of eleven-year-old pupils. London, UK: University of London Press, 1949.

35 Deary IJ, Gow AJ, Taylor MD, et al. The Lothian Birth Cohort 1936: a study to examine influences on cognitive ageing from age 11 to age 70 and beyond. *BMC Geriatr* 2007;7:28. doi: 10.1186/1471-2318-7-28 [published Online First: 5 December 2007].

36 Deary IJ, Gow AJ, Pattie A, et al. Cohort profile: the Lothian Birth Cohorts of 1921 and 1936. *Int J Epidemiol* 2012;41(6):1576-84. doi:10.1093/ije/dyr197 [published Online First: 14 December 2011].

37 Davis TC, Long SW, Jackson RH, et al. Rapid estimate of adult literacy in medicine: a shortened screening instrument. *Fam Med* 1993;25(6):391-95.

38 Dumenci L, Matsuyama RK, Kuhn L, et al. On the Validity of the Rapid Estimate of Adult Literacy in Medicine (REALM) Scale as a Measure of Health Literacy. *Commun Methods Meas* 2013;7(2):134-43. doi:10.1080/19312458.2013.789839 [published Online First: 6 June 2013].

39 Mancuso JM. Assessment and measurement of health literacy: an integrative review of the literature. *Nurs Health Sci* 2009;11(1):77-89. doi:10.1111/j.1442-2018.2008.00408.x [published Online First: 10 February 2009].

40 Weiss BD, Mays MZ, Martz W, et al. Quick assessment of literacy in primary care: the newest vital sign. *Ann Fam Med* 2005;3(6):514-22. doi:10.1370/afm.405.

41 Wechsler D. WAIS-III UK administration and scoring manual. London, UK: Psychological Corporation, 1998.

42 General Register Office. Census 1951: Classification of Occupatioans. London, UK, 1956.

43 Zigmond AS, Snaith RP. The Hospital Anxiety and Depression Scale. *Acta Psychiatr Scand* 1983;67(6):361-70. doi:10.1111/j.1600-0447.1983.tb09716.x.

For peer review only

44 Townsend P. Poverty in the United Kingdom: a survey of household resources and standards of living. Harmondsworth, UK: Penguin, 1979.

45 Folstein MF, Folstein SE, McHugh PR. "Mini-mental state": a practical method for grading the cognitive state of patients for the clinician. *J Psychiatr Res* 1975;12(3):189-98. doi:10.1016/0022-3956(75)90026-6.

46 Sørensen K, Van den Broucke S, Pelikan JM, et al. Measuring health literacy in populations: illuminating the design and development process of the European Health Literacy Survey Questionnaire (HLS-EU-Q). *BMC Public Health* 2013;13(1):948. doi:10.1186/1471-2458-13-948. [published Online First: 10 October 2013].

For peer review only

For peer review only - http://bmjopen.bmj.com/site/about/guidelines.xhtml

Figure 1 Flow diagram of the sample used to investigate the role of cognitive ability in the association between health literacy and mortality (n = 795)

For peer review only

For peer review only - http://bmjopen.bmj.com/site/about/guidelines.xhtml

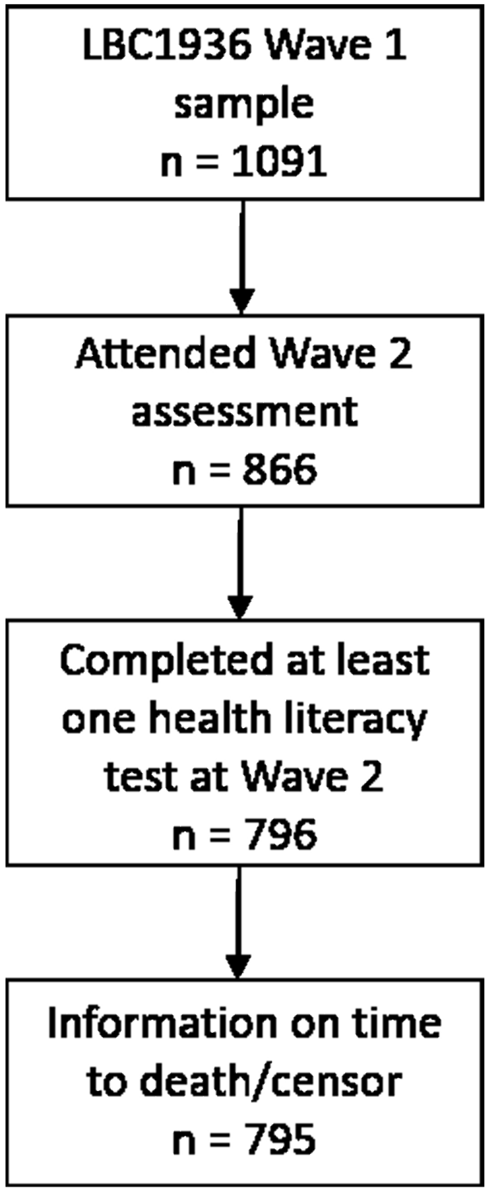

Figure 1 Flow diagram of the sample used to investigate the role of cognitive ability in the association between health literacy and mortality (n = 795)

90x225mm (300 x 300 DPI)

Supplementary material for: The role of cognitive ability in the association between functional health literacy and mortality in the Lothian Birth Cohort 1936: a prospective cohort study

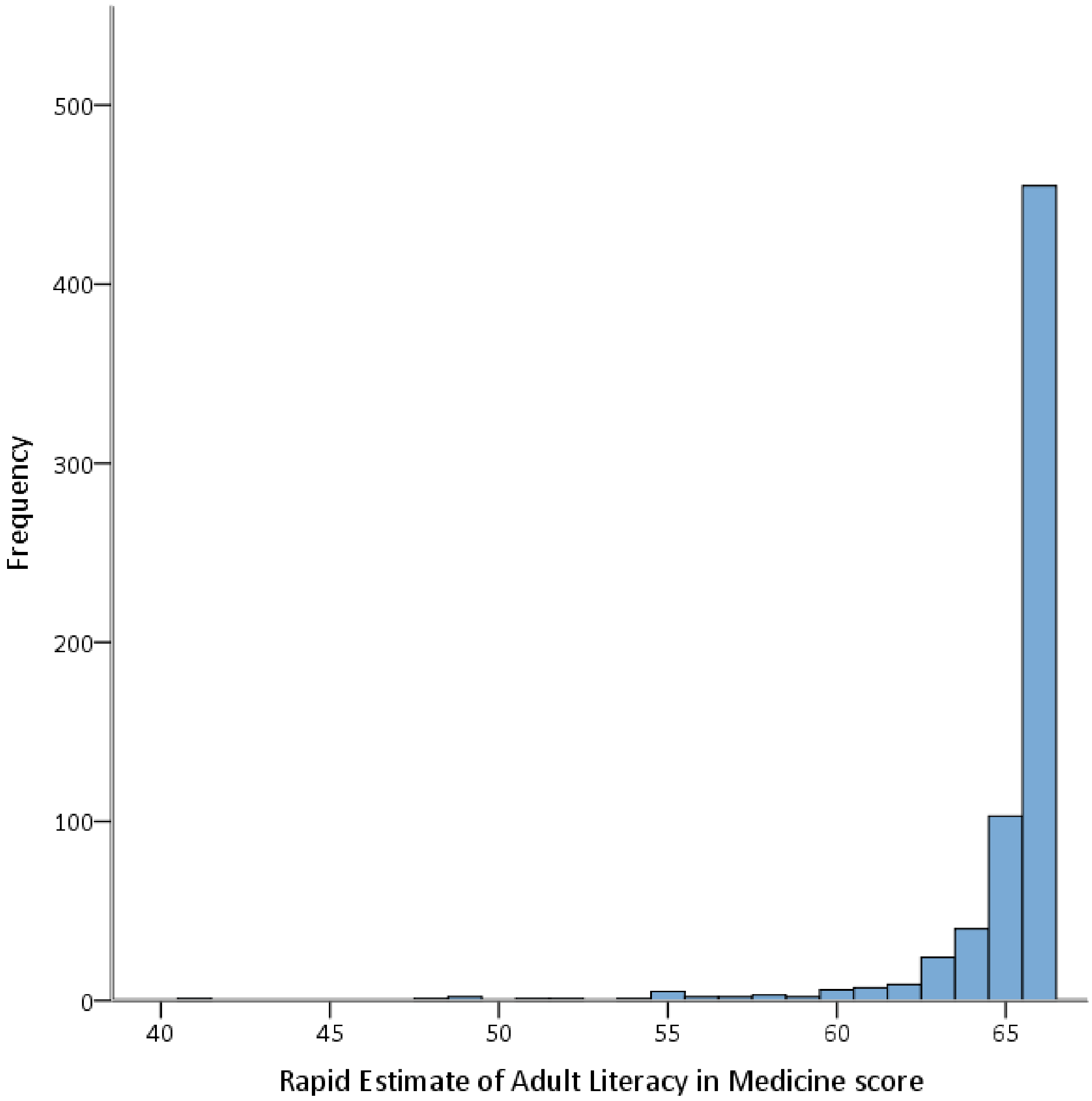

Supplementary Figure 1 Distribution of scores on the Rapid Estimate of Adult Literacy in Medicine for participants who were alive at censoring date

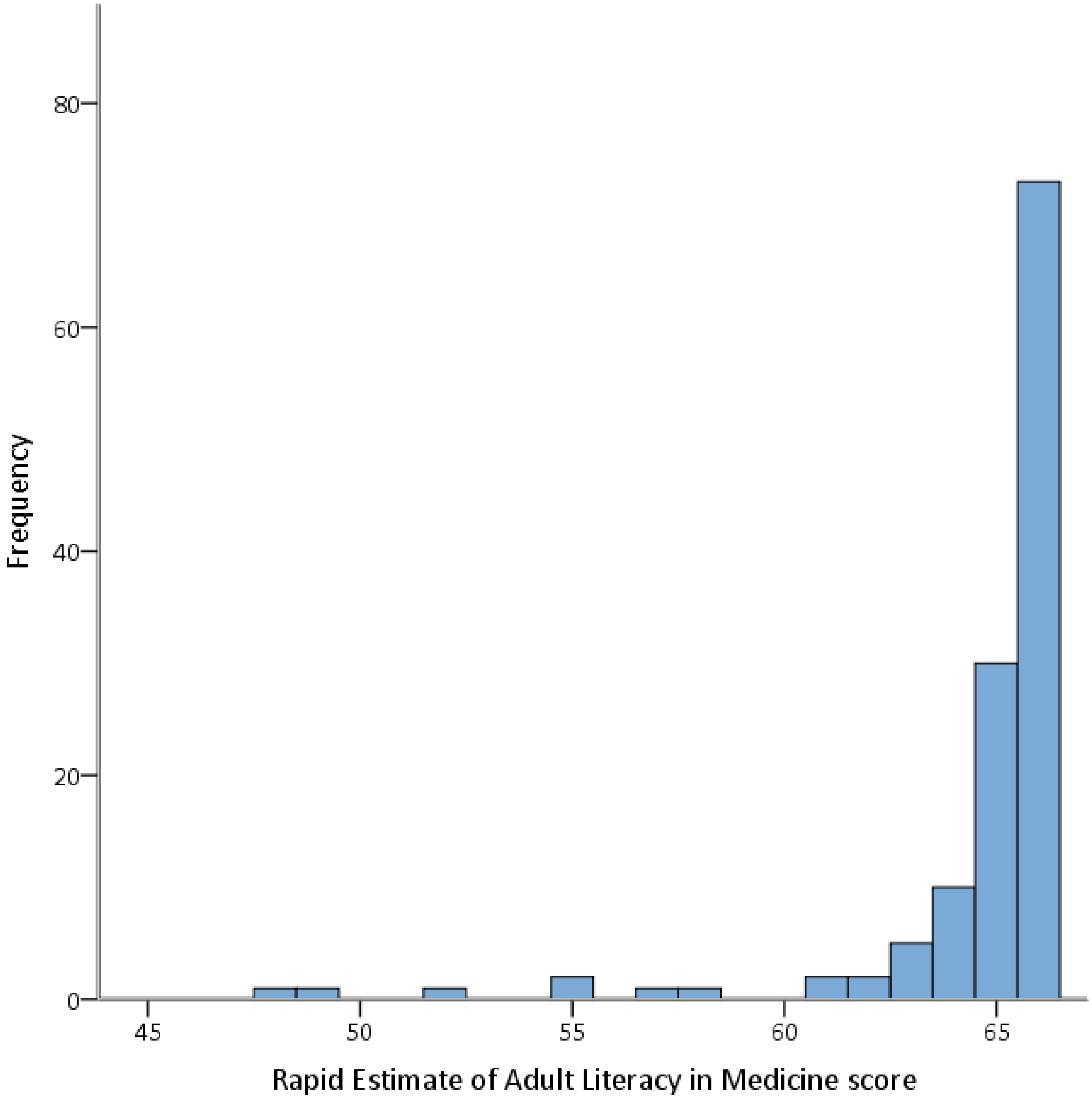

Supplementary Figure 2 Distribution of scores on the Rapid Estimate of Adult Literacy in Medicine for participants who had died by censoring date

For peer review only - http://bmjopen.bmj.com/site/about/guidelines.xhtml

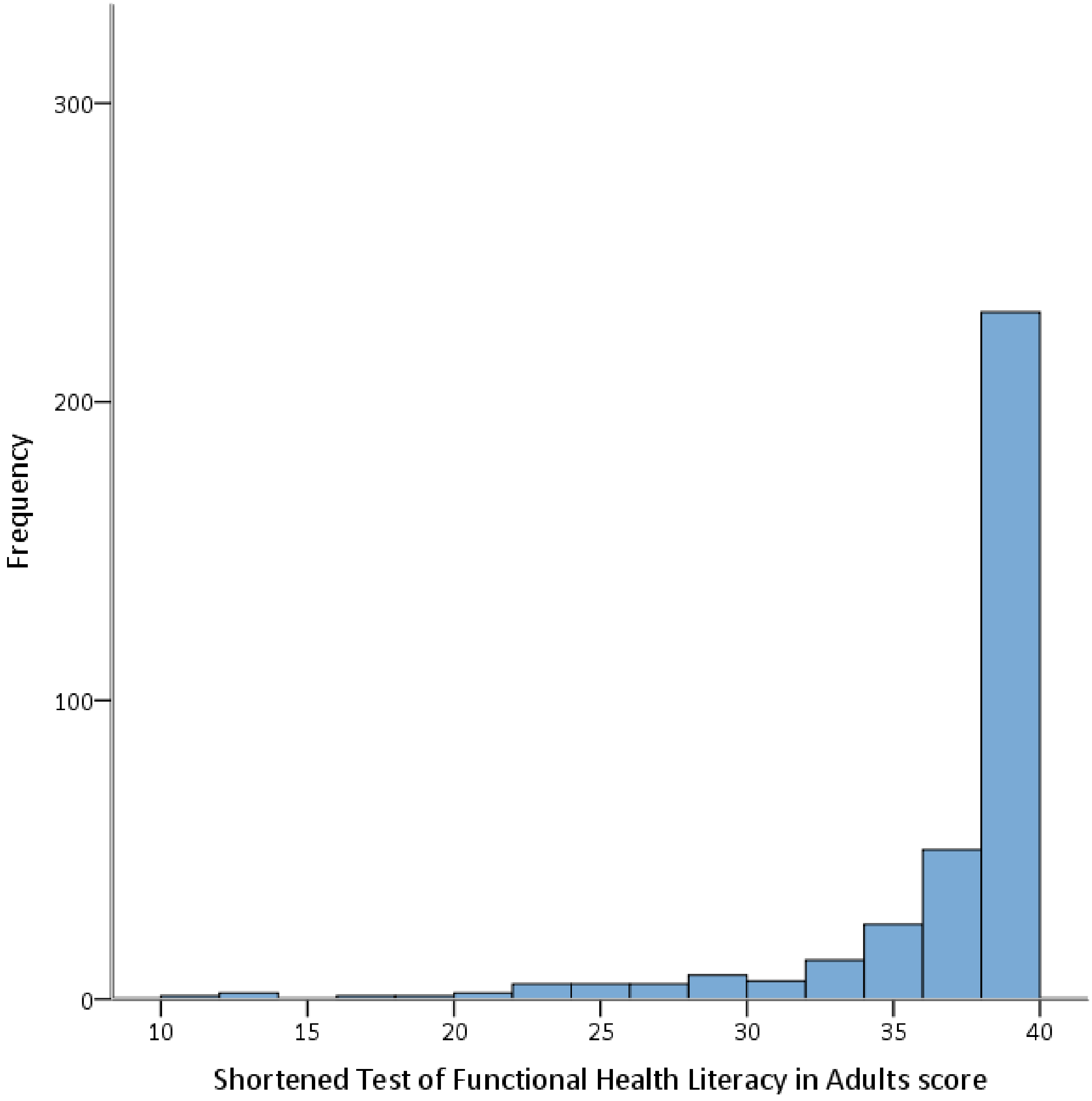

Supplementary Figure 3 Distribution of scores on the Shortened Test of Functional Health Literacy in Adults for participants who were alive at censoring date

For peer review only - http://bmjopen.bmj.com/site/about/guidelines.xhtml

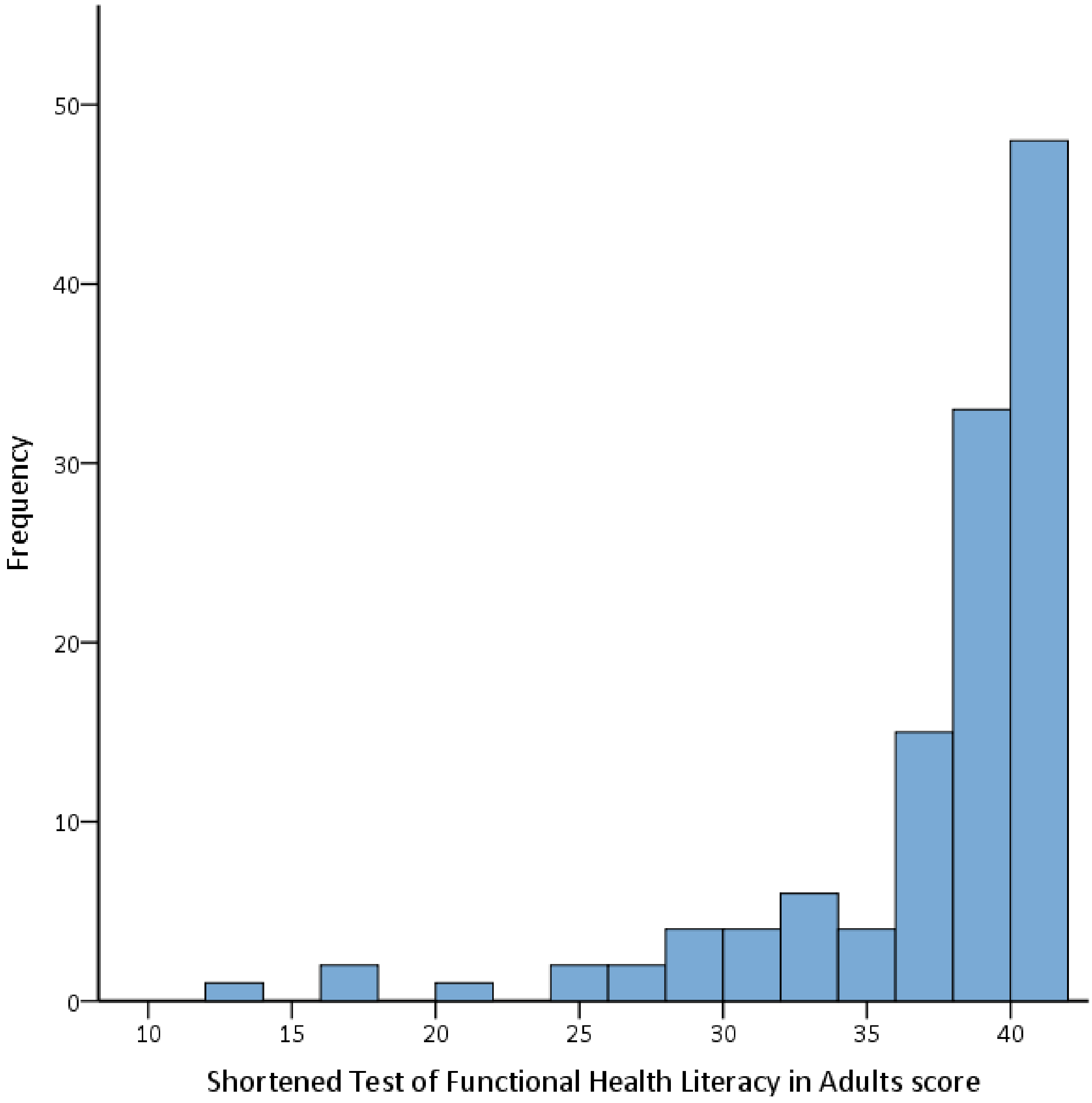

Supplementary Figure 4 Distribution of scores on the Shortened Test of Functional Health Literacy in Adults for participants who has died by censoring date

For peer review only - http://bmjopen.bmj.com/site/about/guidelines.xhtml

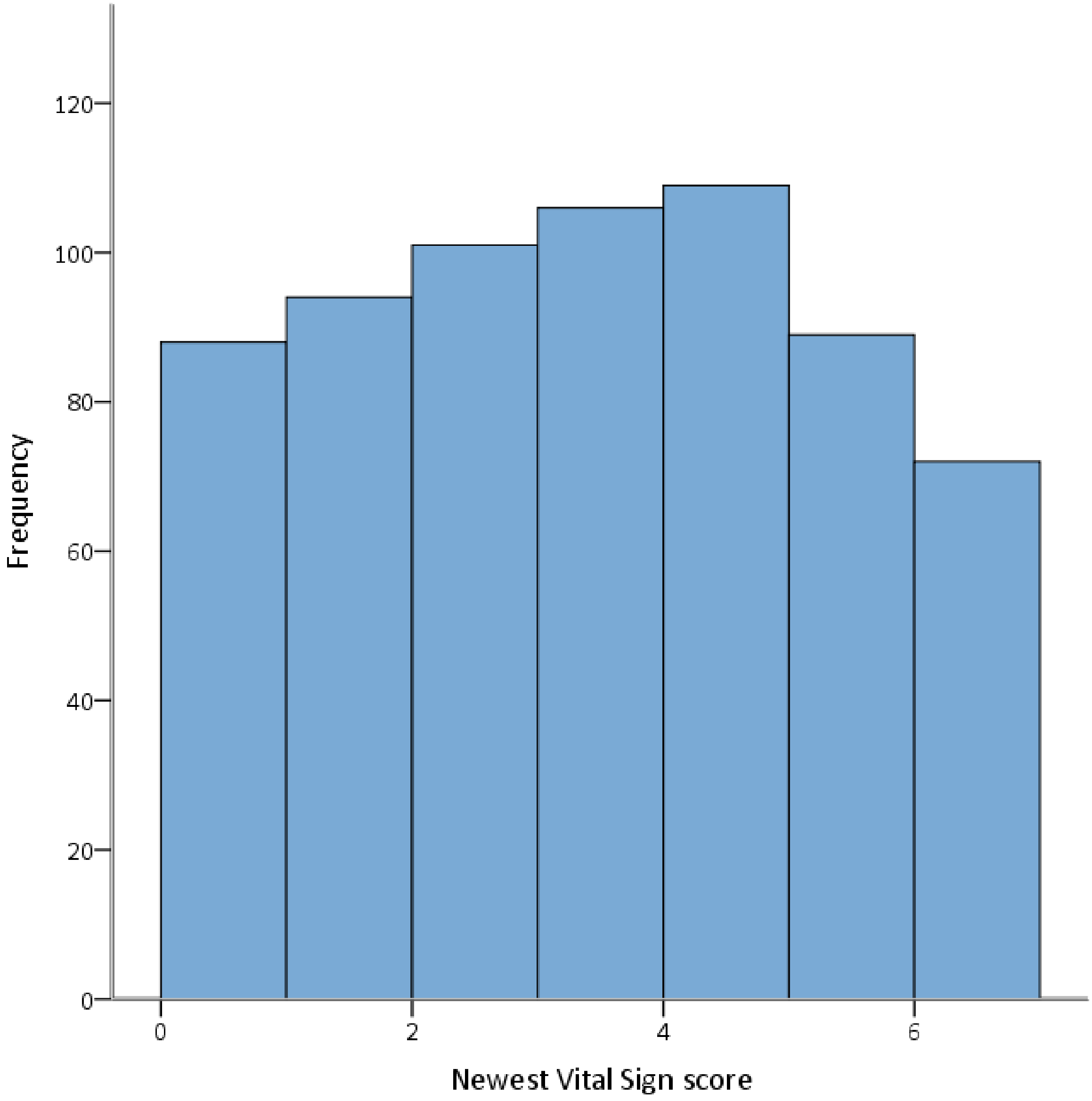

Supplementary Figure 5 Distribution of scores on the Newest Vital Sign for participants who were alive at censoring date

For peer review only - http://bmjopen.bmj.com/site/about/guidelines.xhtml

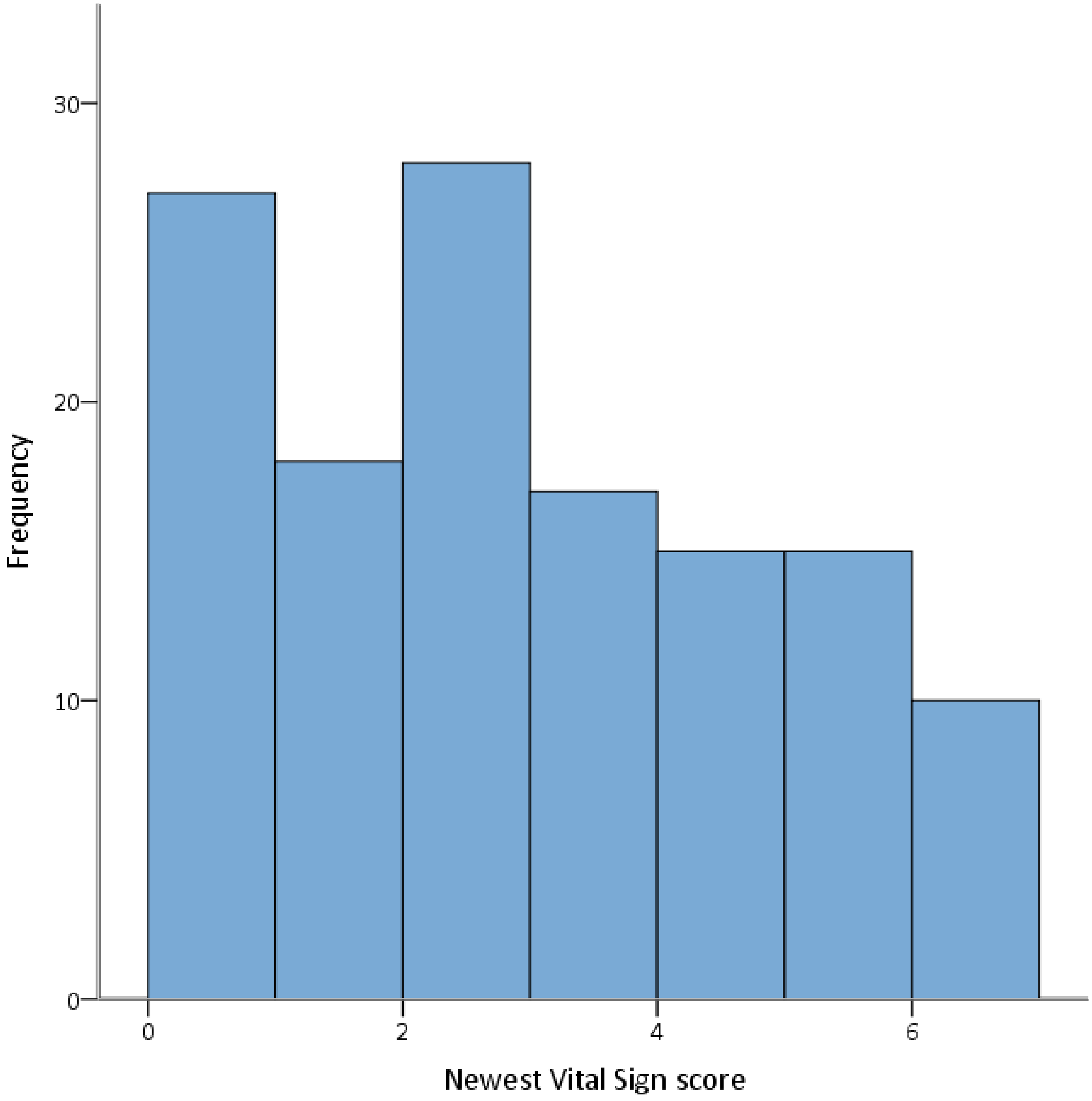

Supplementary Figure 6 Distribution of scores on the Newest Vital Sign for participants who had died by censoring date

For peer review only - http://bmjopen.bmj.com/site/about/guidelines.xhtml

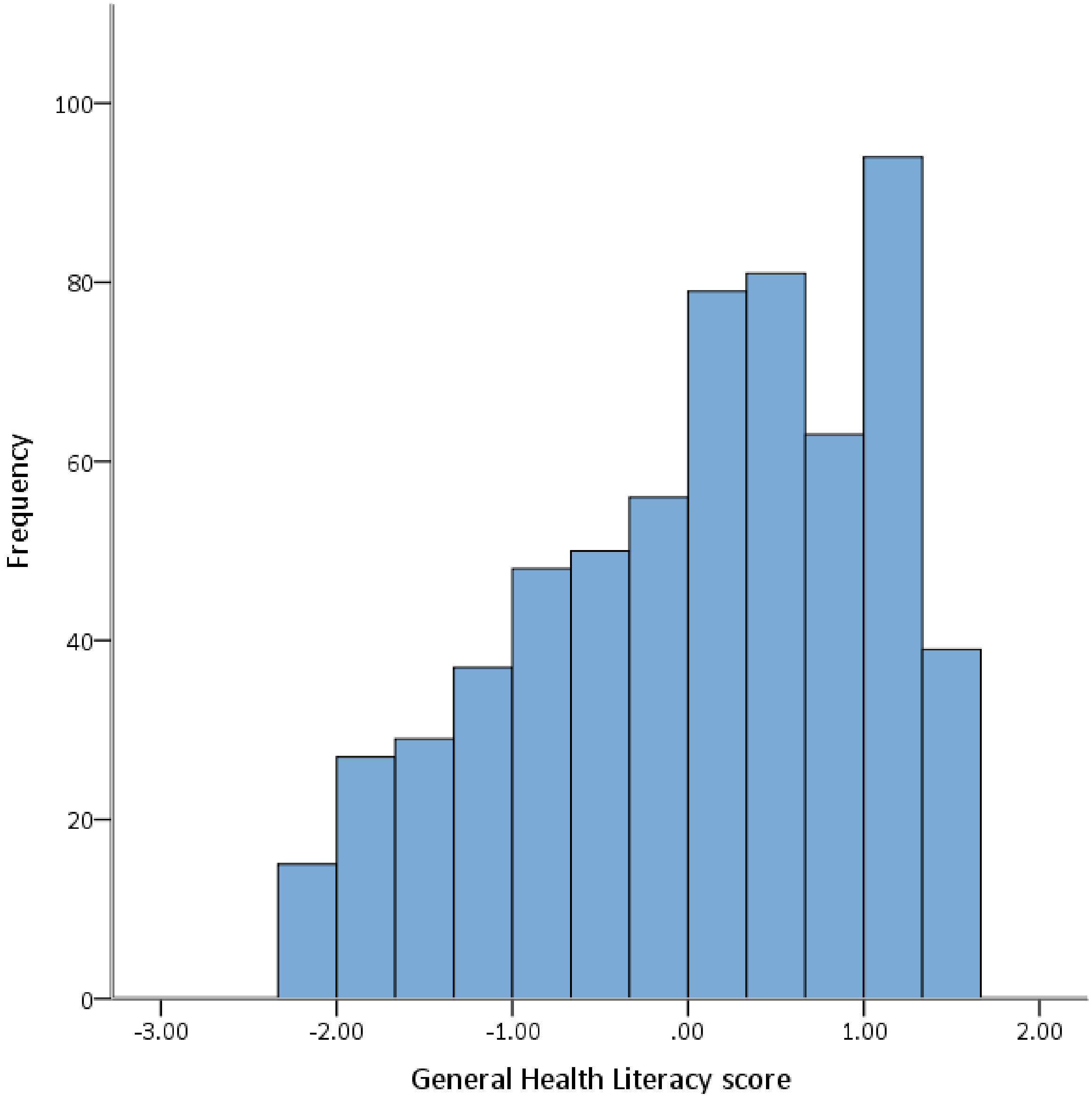

Supplementary Figure 7 Distribution of scores on General Health Literacy for participants who were alive at censoring date

For peer review only - http://bmjopen.bmj.com/site/about/guidelines.xhtml

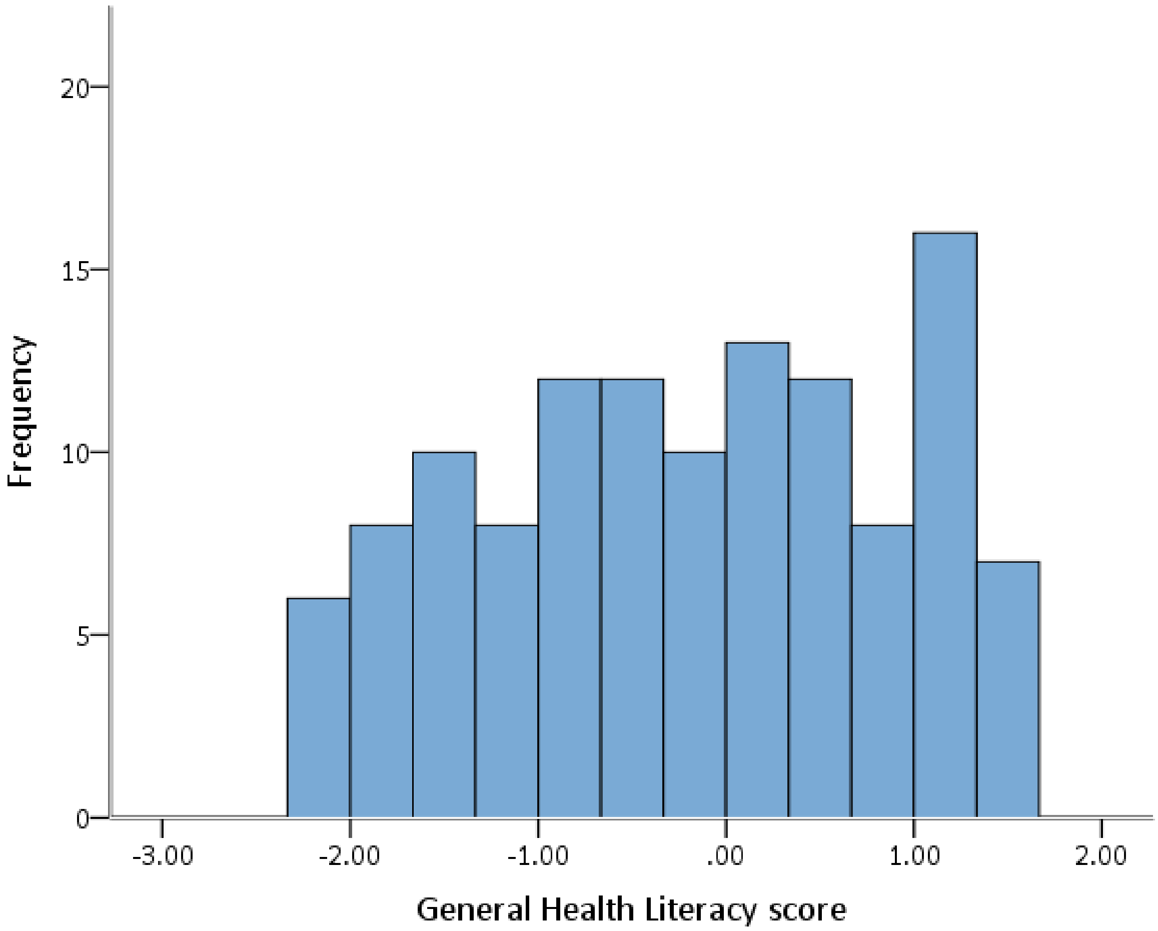

Supplementary Figure 8 Distribution of scores on General Health Literacy for participants who had died by censoring date

For peer review only - http://bmjopen.bmj.com/site/about/guidelines.xhtml

Supplementary Table 1 Rank order correlations between sociodemographic, functional health literacy, cognitive and health variables

	1	2	3	4	5	6	7	8	9	10	11	12	13
1 Age	-												
2 Sex†	0.04	-											
3 REALM	-0.12**	0.17***	-										
4 S-TOFHLA	-0.05	0.10**	0.40***	-									
5 NVS	-0.12**	0.01	0.35***	0.44***	-								
6 General functional health literacy	-0.09*	0.14***	0.71***	0.80***	0.78***	-							
7 Age-11 IQ	-0.07*	0.11**	0.44***	0.48***	0.51***	0.61***	-						
8 Fluid ability	-0.13***	0.00	0.38***	0.55***	0.55***	0.63***	0.57***	-					
9 Education	-0.05	0.03	0.31***	0.33***	0.37***	0.45***	0.45***	0.37***	-				
10 Occup class	0.05	-0.15***	-0.31***	-0.31***	-0.32***	-0.39***	-0.40***	-0.35***	-0.47***	-			
11 Self-rated health	-0.02	0.06	0.12**	0.20***	0.11**	0.18***	0.17***	0.24***	0.11**	-0.11**	-		
12 HADS	0.06	0.08*	-0.07	-0.13**	-0.11**	-0.14***	-0.13***	-0.22***	-0.08*	0.08*	-0.32***	-	
13 Townsend	0.13***	0.16***	-0.08*	-0.12**	-0.15***	-0.14***	-0.12**	-0.17***	-0.12**	0.09*	-0.35***	0.22***	-

**p* < .05, ***p* < .01, ****p* < .001.

†Correlations are point-biseral correlations. Female is coded 1 and male is coded 2.

Occupational class (ranging from 1-professional to 4-manual) and self-rated health (ranging from 1-poor/fair to 3-very good/excellent) are entered as ordinal variables.

REALM, Rapid Estimate of Adult Literacy in Medicine; S-TOFHLA, Shortened Test of Functional Health Literacy in Adults; NVS, Newest Vital Sign; IQ, Intelligence Quotient; Occup class, occupational class; HADS, Hospital Anxiety and Depression Scale; Townsend, Townsend Disability Scale.

For peer review only - http://bmjopen.bmj.com/site/about/guidelines.xhtml

For peer review only

DETAILED RESULTS

REALM: Hazard ratios (HRs) and 95% confidence intervals (CIs) for the associations between the REALM and mortality are shown in Supplementary Table 2. In Model 1, in which age and sex were controlled, the REALM did not significantly predict mortality (HR = 0.954, 95% CI 0.904 to 1.007), nor did age or sex. The REALM remained a non-significant predictor of mortality in Model 2, with the addition of years of education. Years of education did not predict mortality (HR = 0.963, 95% CI 0.822 to 1.128). Age-11 IQ was added in Model 3, and this did little to change the association between the REALM and mortality. Age-11 IQ did not predict mortality (HR = 0.993, 95% CI 0.980 to 1.006). The REALM remained a non-significant predictor of mortality following the inclusion of current fluid ability in Model 4. A one SD increase in fluid ability score reduced the risk of death by 37.9% (HR = 0.621, 95% CI 0.496 to 0.777). In Model 5, occupational social class was included in the model. The REALM remained non-significant. Individuals with a managerial/technical social class (HR = 2.278, 95% CI 1.161 to 4.470), a skilled non-manual social class (HR = 2.464, 95% CI 1.167 to 5.201) or a skilled manual social class (HR = 3.608, 95% CI 1.647 to 7.907) had a higher risk of death than individuals with a professional social class. Health status variables were additionally added in Model 6. The REALM remained a non-significant predictor of mortality. In this model, individuals with more years of education had a higher risk of dying (HR = 1.232, 95% CI 1.018 to 1.492). Risk of death for those who self-reported their health as fair or poor was over 2 times greater than those who reported their health to be very good or excellent (HR = 2.071, 95% CI 1.147 to 3.739). Whereas HADS score did not predict mortality, Townsend disability did. A one-point increase on the Townsend disability scale increased risk of mortality by 13.3% (HR = 1.133, 95% CI 1.044 to 1.229).

S-TOFHLA: The HRs for the association between S-TOFHLA and mortality are shown in Supplementary Table 3. In Model 1, controlling for age and sex, S-TOFHLA significantly predicted mortality. A one-point increase in S-TOFHLA reduced the risk of death by 5.2% (HR = 0.948, 95% CI 0.919 to 0.978). In this model, age and sex did not predict mortality. Adding years of education in Model 2 did not change the association between the S-TOFHLA and mortality. Years of education did

For peer review only - http://bmjopen.bmj.com/site/about/guidelines.xhtml

not predict mortality (HR = 1.020, 95% CI 0.870 to 1.197). The inclusion of age-11 IQ in Model 3 did not change the association between the S-TOFHLA and mortality. Age-11 IQ also did not predict mortality (HR = 0.997, 95% CI 0.983 to 1.011). The association between the S-TOFHLA and mortality was attenuated and became non-significant (HR = 0.967, 95% CI 0.929 to 1.007) in Model 4, additionally accounting current fluid ability. Current fluid ability significantly predicted mortality in this model. A one SD increase in fluid ability reduced the risk of death by 30.5% (HR = 0.695, 95% CI 0.545 to 0.887). Occupational class was included in Model 5, and the association between S-TOFHLA and mortality remained non-significant. Individuals with more years of education, controlling for other sociodemographic variables and cognitive function, had increased risk of death (HR = 1.219, 95% CI 1.004 to 1.481). Risk of dying was three times greater for participants with a skilled manual social class, compared to individuals with a professional social class (HR = 3.096, 95% CI 1.385 to 6.922). S-TOFHLA remained a non-significant predictor or mortality in Model 6, which included health status variables. Self-reporting health as fair or poor, compared to very good or excellent, was associated with increased risk of mortality (HR = 2.209, 95% CI 1.216 to 4.014). Higher scores on the HADS were not associated with mortality, while a higher Townsend disability score increased risk of death (HR = 1.131, 95% CI 1.039 to 1.232).

NVS: HRs for the association between NVS and mortality are shown in Supplementary Table 4. In Model 1, in which age and sex were entered as covariates, NVS significantly predicted mortality. A one point increase in NVS score reduced the risk of death by 11.8% (HR = 0.882, 95% CI 0.805 to 0.966). Age and sex did not predict mortality. Years of education was included in Model 2 and this did not change the association between the NVS and mortality. Years of education did not predict mortality (HR = 1.007, 95% CI 0.855 to 1.186). Age-11 IQ was additionally added to the model in Model 3 and this did little to change the association between NVS and mortality and this association remained significant. Age-11 IQ did not predict mortality (HR = 0.995, 95% CI 0.982 to 1.008). The inclusion of fluid ability in Model 4 greatly attenuated the association between NVS and mortality, and this became non-significant (HR = 0.963, 95% CI 0.860 to 1.078). Fluid ability was strongly associated with risk of death. A one SD increase in fluid ability score reduced risk of dying by 37.0%

For peer review only

For peer review only - http://bmjopen.bmj.com/site/about/guidelines.xhtml

(HR = 0.630, 95% CI 0.496 to 0.800). The association between NVS and mortality remained non-significant in Model 5 following inclusion of occupational class in the model. Compared to those with a professional social class, participants with managerial or technical (HR = 2.288, 95% CI 1.166 to 4.490), skilled non-manual (HR = 2.421, 95% CI 1.147 to 5.112), and skilled manual (HR = 3.631, 95% CI 1.658 to 7.951) social class had an increased risk of death. Finally, health status variables were included in Model 6. The inclusion of health status variables did little to change the association between NVS and mortality, which remained non-significant. In this model, having more years of education was associated with increased risk of mortality (HR = 1.242, 95% CI 1.023 to 1.508). Those who reported their health as fair or poor had 2.10 times (HR = 2.099, 95% CI 1.167 to 3.775) increased risk of mortality, compared to those who self-reported their health as very good or excellent. Participants with higher scores on the Townsend disability scale also had an increased risk of mortality (HR = 1.132, 95% CI 1.044 to 1.228).

General functional health literacy: HRs for the association between general functional health literacy and mortality are shown in Supplementary Table 5. General functional health literacy predicted mortality in Model 1 (HR = 0.774, 95% CI 0.650 to 0.922), while age and sex did not. A one point increase in the general functional health literacy score reduced the risk of mortality by 22.6%. Adding years of education (Model 2) did little to change the association between general functional health literacy and mortality and this association remained significant. Years of education was not associated with mortality (HR = 1.080, 95% CI 0.909 to 1.284). General functional health literacy remained a significant predictor of mortality when age-11 IQ was added in Model 3. Age-11 IQ did not predict morality (HR = 0.999, 95% CI 0.984 to 1.014). The inclusion of current fluid ability in Model 4 attenuated the association between general functional health literacy and risk of death, and this association became non-significant (HR = 0.871, 95% CI 0.674 to 1.125). Fluid ability was a significant predictor of mortality, such that a one SD increase in fluid ability reduced risk of death by 31.3% (HR = 0.687, 95% CI 0.531 to 0.887). Including occupational social class in Model 5 did little to change the association between general functional health literacy and mortality, and this association remained non-significant. In Model 5, individuals with more years of education had a greater risk of

For peer review only - http://bmjopen.bmj.com/site/about/guidelines.xhtml

death (HR = 1.240, 95% CI 1.019 to 1.508), and those with an occupational social class of skilled manual (HR = 3.134, 95% CI 1.405 to 6.991), when compared to those with a professional occupational class, had an increased risk of mortality. Finally, health status variables were added in Model 6. The association between general functional health literacy and mortality was attenuated further and remained non-significant. Reporting fair or poor health, compared to reporting very good or excellent health increased the risk of mortality (HR = 2.229, 95% CI 1.229 to 4.042). Higher Townsend disability scores were also associated with increased risk of death (HR = 1.128, 95% CI 1.040 to 1.225). In this final model, controlling for sociodemographics and health variables, as well as age-11 IQ, the association between fluid ability and mortality was attenuated and became non-significant (HR = 0.770, 95% CI 0.589 to 1.007).

For peer review only

Supplementary Table 2 Hazard ratios (95% confidence intervals) for the association between REALM and mortality, controlling for sociodemographic, cognitive ability, and health status variables

	Model 1<br>Age and sex<br>N = 794	Model 2<br>+ education<br>N = 794	Model 3<br>+ age-11 IQ<br>N = 752	Model 4<br>+ current fluid ability<br>N = 746	Model 5<br>+ occup class<br>N = 731	Model 6<br>+ health status<br>N = 728
REALM	0.954 (0.904 to 1.007)	0.957 (0.905 to 1.013)	0.962 (0.903 to 1.025)	0.971 (0.907 to 1.039)	0.970 (0.904 to 1.040)	0.996 (0.924 to 1.074)
Age	0.940 (0.725 to 1.219)	0.939 (0.724 to 1.218)	0.944 (0.725 to 1.231)	0.879 (0.669 to 1.154)	0.908 (0.686 to 1.203)	0.933 (0.704 to 1.235)
Sex						
Female	Reference	Reference	Reference	Reference	Reference	Reference
Male	1.297 (0.909 to 1.850)	1.298 (0.910 to 1.852)	1.252 (0.869 to 1.802)	1.333 (0.927 to 1.918)	1.176 (0.787 to 1.756)	1.364 (0.898 to 2.073)
Years of education		0.963 (0.822 to 1.128)	1.022 (0.862 to 1.211)	1.089 (0.916 to 1.295)	1.201 (0.995 to 1.450)	1.232 (1.018 to 1.492)*
Age-11 IQ			0.993 (0.980 to 1.006)	1.008 (0.993 to 1.023)	1.009 (0.994 to 1.024)	1.008 (0.993 to 1.024)
Fluid ability				0.621 (0.496 to 0.777)***	0.662 (0.526 to 0.834)***	0.727 (0.574 to 0.922)**
Occupational class						
Professional					Reference	Reference
Managerial/technical					2.278 (1.161 to 4.470)*	2.218 (1.127 to 4.365)*
Skilled: non-manual					2.464 (1.167 to 5.201)*	2.596 (1.232 to 5.474)*
Skilled: manual					3.608 (1.647 to 7.907)**	3.393 (1.532 to 7.516)**
Partly skilled/ unskilled manual					2.054 (0.651 to 6.473)	2.067 (0.656 to 6.510)
Self-rated health						
Very good/excellent						Reference
Good						1.153 (0.742 to 1.791)
Fair/poor						2.071 (1.147 to 3.739)*
HADS total score						0.972 (0.929 to 1.018)
Townsend disability						1.133 (1.044 to 1.229)**

* $p < .05$, ** $p < .01$, *** $p < .001$.

REALM, Rapid Estimate of Adult Literacy in Medicine; IQ, Intelligence Quotient; occup class, Occupational class; HADS, Hospital Anxiety and Depression Scale.

For peer review only - http://bmjopen.bmj.com/site/about/guidelines.xhtml

Supplementary Table 3 Hazard ratios (95% confidence intervals) for the association between S-TOFHLA and mortality, controlling for sociodemographic, cognitive ability, and health variables

	Model 1<br>Age and sex<br>N = 744	Model 2<br>+ education<br>N = 744	Model 3<br>+ age-11 IQ<br>N = 702	Model 4<br>+ current fluid ability<br>N = 697	Model 5<br>+ occup class<br>N = 682	Model 6<br>+ health status<br>N = 680
S-TOFHLA	0.948 (0.919 to 0.978)**	0.947 (0.917 to 0.978)**	0.947 (0.913 to 0.982)**	0.967 (0.929 to 1.007)	0.976 (0.935 to 1.019)	0.998 (0.953 to 1.046)
Age	0.882 (0.665 to 1.170)	0.882 (0.665 to 1.170)	0.889 (0.666 to 1.186)	0.871 (0.652 to 1.164)	0.919 (0.682 to 1.238)	0.936 (0.697 to 1.256)
Sex						
Female	Reference	Reference	Reference	Reference	Reference	Reference
Male	1.307 (0.909 to 1.879)	1.309 (0.910 to 1.881)	1.277 (0.881 to 1.851)	1.349 (0.930 to 1.956)	1.204 (0.797 to 1.818)	1.352 (0.881 to 2.074)
Years of education		1.020 (0.870 to 1.197)	1.066 (0.896 to 1.268)	1.111 (0.932 to 1.326)	1.219 (1.004 to 1.481)*	1.249 (1.026 to 1.520)*
Age-11 IQ			0.997 (0.983 to 1.011)	1.006 (0.991 to 1.022)	1.007 (0.991 to 1.022)	1.006 (0.991 to 1.022)
Fluid ability				0.695 (0.545 to 0.887)**	0.717 (0.557 to 0.922)*	0.759 (0.587 to 0.982)*
Occupational class						
Professional					Reference	Reference
Managerial/technical					1.889 (0.956 to 3.734)	1.844 (0.931 to 3.650)
Skilled: non-manual					2.108 (0.994 to 4.470)	2.207 (1.042 to 4.673)*
Skilled: manual					3.096 (1.385 to 6.922)**	2.881 (1.275 to 6.509)*
Partly skilled/ unskilled manual					1.786 (0.566 to 5.636)	1.773 (0.562 to 5.598)
Self-rated health						
Very good/excellent						Reference
Good						1.147 (0.728 to 1.807)
Fair/poor						2.209 (1.216 to 4.014)**
HADS total score						0.974 (0.930 to 1.021)
Townsend disability						1.131 (1.039 to 1.232)**

*$p < .05$, **$p < .01$, ***$p < .001$.

S-TOFHLA, Shortened Test of Functional Health Literacy in Adults; IQ, Intelligence Quotient; occup class, Occupational class; HADS, Hospital Anxiety and Depression Scale.

For peer review only - http://bmjopen.bmj.com/site/about/guidelines.xhtml

Supplementary Table 4 Hazard ratios (95% confidence intervals) for the association between NVS and mortality, controlling for sociodemographic, cognitive ability, and health variables

	Model 1<br>Age and sex<br>N = 789	Model 2<br>+ education<br>N = 789	Model 3<br>+ age-11 IQ<br>N = 746	Model 4<br>+ current fluid ability<br>N = 742	Model 5<br>+ occup class<br>N = 727	Model 6<br>+ health status<br>N = 724
NVS	0.882 (0.805 to 0.966)**	0.880 (0.799 to 0.970)*	0.892 (0.802 to 0.992)*	0.963 (0.860 to 1.078)	0.967 (0.861 to 1.086)	0.961 (0.853 to 1.082)
Age	0.942 (0.727 to 1.221)	0.942 (0.726 to 1.221)	0.942 (0.722 to 1.228)	0.890 (0.678 to 1.168)	0.919 (0.694 to 1.217)	0.937 (0.708 to 1.242)
Sex						
Female	Reference	Reference	Reference	Reference	Reference	Reference
Male	1.343 (0.946 to 1.906)	1.343 (0.947 to 1.907)	1.279 (0.892 to 1.834)	1.346 (0.939 to 1.928)	1.180 (0.791 to 1.760)	1.355 (0.893 to 2.057)
Years of education		1.007 (0.855 to 1.186)	1.056 (0.888 to 1.257)	1.093 (0.917 to 1.302)	1.208 (0.998 to 1.463)	1.242 (1.023 to 1.508)*
Age-11 IQ			0.995 (0.982 to 1.008)	1.007 (0.993 to 1.021)	1.008 (0.993 to 1.023)	1.009 (0.994 to 1.023)
Fluid ability				0.630 (0.496 to 0.800)***	0.670 (0.524 to 0.857)**	0.748 (0.580 to 0.966)*
Occupational class						
Professional					Reference	Reference
Managerial/technical					2.288 (1.166 to 4.490)*	2.243 (1.140 to 4.414)*
Skilled: non-manual					2.421 (1.147 to 5.112)*	2.593 (1.231 to 5.463)*
Skilled: manual					3.631 (1.658 to 7.951)**	3.360 (1.522 to 7.415) **
Partly skilled/ unskilled manual					2.125 (0.677 to 6.669)	2.086 (0.661 to 6.578)
Self-rated health						
Very good/excellent						Reference
Good						1.175 (0.756 to 1.826)
Fair/poor						2.099 (1.167 to 3.775)*
HADS total score						0.973 (0.930 to 1.018)
Townsend disability						1.132 (1.044 to 1.228)**

*p < .05, **p < .01.

NVS, Newest Vital Sign; IQ, Intelligence Quotient; occup class, Occupational class; HADS, Hospital Anxiety and Depression Scale.

For peer review only - http://bmjopen.bmj.com/site/about/guidelines.xhtml

Supplementary Table 5 Hazard ratios (95% confidence intervals) for the association between general functional health literacy and mortality, controlling for sociodemographic, cognitive ability, and health variables

	Model 1<br>Age and sex<br>N = 740	Model 2<br>+ education<br>N = 740	Model 3<br>+ age-11 IQ<br>N = 698	Model 4<br>+ current fluid ability<br>N = 694	Model 5<br>+ occup class<br>N = 679	Model 6<br>+ health status<br>N = 677
General functional health literacy	0.774 (0.650 to 0.922)**	0.746 (0.615 to 0.905)**	0.738 (0.585 to 0.931)*	0.871 (0.674 to 1.125)	0.911 (0.700 to 1.186)	0.950 (0.725 to 1.245)
Age	0.897 (0.678 to 1.187)	0.893 (0.675 to 1.182)	0.902 (0.677 to 1.200)	0.885 (0.663 to 1.182)	0.933 (0.693 to 1.257)	0.942 (0.700 to 1.266)
Sex						
Female	Reference	Reference	Reference	Reference	Reference	Reference
Male	1.276 (0.886 to 1.838)	1.272 (0.883 to 1.833)	1.238 (0.852 to 1.799)	1.327 (0.912 to 1.930)	1.178 (0.778 to 1.784)	1.337 (0.869 to 2.056)
Years of education		1.080 (0.909 to 1.284)	1.119 (0.936 to 1.339)	1.134 (0.948 to 1.357)	1.240 (1.019 to 1.508)*	1.255 (1.030 to 1.528)*
Age-11 IQ			0.999 (0.984 to 1.014)	1.006 (0.991 to 1.022)	1.006 (0.991 to 1.022)	1.007 (0.992 to 1.023)
Fluid ability				0.687 (0.531 to 0.887)**	0.707 (0.543 to 0.921)*	0.770 (0.589 to 1.007)
Occupational class						
Professional					Reference	Reference
Managerial/technical					1.901 (0.962 to 3.756)	1.870 (0.945 to 3.700)
Skilled: non-manual					2.076 (0.979 to 4.401)	2.192 (1.035 to 4.640)*
Skilled: manual					3.134 (1.405 to 6.991)**	2.823 (1.252 to 6.365)*
Partly skilled/ unskilled manual					1.824 (0.580 to 5.741)	1.759 (0.557 to 5.561)
Self-rated health						
Very good/excellent						Reference
Good						1.152 (0.733 to 1.810)
Fair/poor						2.229 (1.229 to 4.042)**
HADS total score						0.975 (0.931 to 1.022)
Townsend disability						1.128 (1.040 to 1.225)**

* $p < .05$, ** $p < .01$.

General functional health literacy, general measure of functional health literacy created by entering the REALM, S-TOFHLA and NVS into a PCA; IQ, Intelligence Quotient; occup class, Occupational class; HADS, Hospital Anxiety and Depression Scale.

For peer review only - http://bmjopen.bmj.com/site/about/guidelines.xhtml

For peer review only

Supplementary Table 6 Hazard ratios (95% confidence intervals) for the association between REALM and mortality, controlling for sociodemographic, cognitive ability, and health status variables. Models are run on a sub-sample participants with all variables of interest (N = 728).

	Model 1<br>Age and sex	Model 2<br>+ education	Model 3<br>+ age-11 IQ	Model 4<br>+ current fluid ability	Model 5<br>+ occup class	Model 6<br>+ health status
REALM	0.944 (0.894 to 0.997)*	0.946 (0.894 to 1.001)	0.959 (0.900 to 1.021)	0.966 (0.904 to 1.033)	0.969 (0.904 to 1.039)	0.996 (0.924 to 1.074)
Age	1.002 (0.763 to 1.316)	1.001 (0.762 to 1.315)	0.999 (0.761 to 1.312)	0.931 (0.704 to 1.231)	0.930 (0.700 to 1.234)	0.933 (0.704 to 1.235)
Sex						
Female	Reference	Reference	Reference	Reference	Reference	Reference
Male	1.303 (0.897 to 1.892)	1.304 (0.898 to 1.893)	1.289 (0.887 to 1.872)	1.358 (0.935 to 1.971)	1.224 (0.815 to 1.836)	1.364 (0.898 to 2.073)
Years of education		0.981 (0.831 to 1.158)	1.010 (0.848 to 1.204)	1.077 (0.902 to 1.287)	1.203 (0.994 to 1.455)	1.232 (1.018 to 1.492)*
Age-11 IQ			0.993 (0.980 to 1.006)	1.007 (0.992 to 1.023)	1.009 (0.993 to 1.025)	1.008 (0.993 to 1.024)
Fluid ability				0.632 (0.503 to 0.794)***	0.666 (0.528 to 0.841)**	0.727 (0.574 to 0.922)**
Occupational class						
Professional					Reference	Reference
Managerial/technical					2.201 (1.118 to 4.333)*	2.218 (1.127 to 4.365)*
Skilled: non-manual					2.482 (1.175 to 5.245)*	2.596 (1.232 to 5.474)*
Skilled: manual					3.570 (1.627 to 7.837)**	3.393 (1.532 to 7.516)**
Partly skilled/ unskilled manual					2.023 (0.641 to 6.388)	2.067 (0.656 to 6.510)
Self-rated health						
Very good/excellent						Reference
Good						1.153 (0.742 to 1.791)
Fair/poor						2.071 (1.147 to 3.739)*
HADS total score						0.972 (0.929 to 1.018)
Townsend disability						1.133 (1.044 to 1.229)**

*p < .05, **p < .01, ***p < .001.

REALM, Rapid Estimate of Adult Literacy in Medicine; IQ, Intelligence Quotient; occup class, Occupational class; HADS, Hospital Anxiety and Depression Scale.

For peer review only - http://bmjopen.bmj.com/site/about/guidelines.xhtml

For peer review only

Supplementary Table 7 Hazard ratios (95% confidence intervals) for the association between S-TOFHLA and mortality, controlling for sociodemographic, cognitive ability, and health variables. Models are run on a subsample of participants with all variables of interest (N = 680).

	Model 1<br>Age and sex	Model 2<br>+ education	Model 3<br>+ age-11 IQ	Model 4<br>+ current fluid ability	Model 5<br>+ occup class	Model 6<br>+ health status
S-TOFHLA	0.947 (0.917 to 0.978)**	0.945 (0.914 to 0.977)**	0.949 (0.913 to 0.985)**	0.969 (0.930 to 1.010)	0.975 (0.934 to 1.018)	0.998 (0.953 to 1.046)
Age	0.924 (0.688 to 1.242)	0.925 (0.688 to 1.242)	0.927 (0.690 to 1.245)	0.911 (0.677 to 1.224)	0.919 (0.681 to 1.240)	0.936 (0.697 to 1.256)
Sex						
Female	Reference	Reference	Reference	Reference	Reference	Reference
Male	1.304 (0.893 to 1.902)	1.306 (0.895 to 1.905)	1.298 (0.889 to 1.896)	1.356 (0.928 to 1.981)	1.233 (0.814 to 1.866)	1.352 (0.881 to 2.074)
Years of education		1.033 (0.874 to 1.222)	1.046 (0.875 to 1.250)	1.092 (0.911 to 1.309)	1.208 (0.994 to 1.469)	1.249 (1.026 to 1.520)*
Age-11 IQ			0.997 (0.983 to 1.011)	1.006 (0.991 to 1.022)	1.007 (0.992 to 1.023)	1.006 (0.991 to 1.022)
Fluid ability				0.699 (0.545 to 0.895)**	0.717 (0.556 to 0.923)*	0.759 (0.587 to 0.982)*
Occupational class						
Professional					Reference	Reference
Managerial/technical					1.853 (0.935 to 3.670)	1.844 (0.931 to 3.650)
Skilled: non-manual					2.105 (0.992 to 4.464)	2.207 (1.042 to 4.673)*
Skilled: manual					3.038 (1.358 to 6.796)**	2.881 (1.275 to 6.509)*
Partly skilled/ unskilled manual					1.755 (0.556 to 5.541)	1.773 (0.562 to 5.598)
Self-rated health						
Very good/excellent						Reference
Good						1.147 (0.728 to 1.807)
Fair/poor						2.209 (1.216 to 4.014)**
HADS total score						0.974 (0.930 to 1.021)
Townsend disability						1.131 (1.039 to 1.232)**

*$p < .05$, **$p < .01$.

S-TOFHLA, Shortened Test of Functional Health Literacy in Adults; IQ, Intelligence Quotient; occup class, Occupational class; HADS, Hospital Anxiety and Depression Scale.

For peer review only

Supplementary Table 8 Hazard ratios (95% confidence intervals) for the association between NVS and mortality, controlling for sociodemographic, cognitive ability, and health variables. Models are run on a sub-sample of participants with all variables of interest (N = 724).

	Model 1<br>Age and sex	Model 2<br>+ education	Model 3<br>+ age-11 IQ	Model 4<br>+ current fluid ability	Model 5<br>+ occup class	Model 6<br>+ health status
NVS	0.880 (0.800 to 0.968)**	0.875 (0.790 to 0.968)*	0.887 (0.796 to 0.989)*	0.953 (0.850 to 1.070)	0.960 (0.854 to 1.079)	0.961 (0.853 to 1.082)
Age	0.993 (0.756 to 1.306)	0.993 (0.756 to 1.306)	0.992 (0.754 to 1.304)	0.944 (0.714 to 1.248)	0.940 (0.709 to 1.248)	0.937 (0.708 to 1.242)
Sex						
Female	Reference	Reference	Reference	Reference	Reference	Reference
Male	1.346 (0.933 to 1.943)	1.346 (0.933 to 1.943)	1.326 (0.916 to 1.919)	1.373 (0.950 to 1.986)	1.228 (0.820 to 1.840)	1.355 (0.893 to 2.057)
Years of education		1.029 (0.866 to 1.222)	1.048 (0.876 to 1.253)	1.084 (0.905 to 1.298)	1.212 (0.999 to 1.470)	1.242 (1.023 to 1.508)*
Age-11 IQ			0.995 (0.982 to 1.008)	1.006 (0.992 to 1.021)	1.008 (0.993 to 1.023)	1.009 (0.994 to 1.023)
Fluid ability				0.645 (0.506 to 0.822)***	0.678 (0.529 to 0.869)**	0.748 (0.580 to 0.966)*
Occupational class						
Professional					Reference	Reference
Managerial/technical					2.211 (1.123 to 4.354)*	2.243 (1.140 to 4.414)*
Skilled: non-manual					2.435 (1.152 to 5.146)*	2.593 (1.231 to 5.463)*
Skilled: manual					3.590 (1.637 to 7.874)**	3.360 (1.522 to 7.415) **
Partly skilled/ unskilled manual					2.101 (0.668 to 6.604)	2.086 (0.661 to 6.578)
Self-rated health						
Very good/excellent						Reference
Good						1.175 (0.756 to 1.826)
Fair/poor						2.099 (1.167 to 3.775)*
HADS total score						0.973 (0.930 to 1.018)
Townsend disability						1.132 (1.044 to 1.228)**

*$p < .05$, **$p < .01$.

NVS, Newest Vital Sign; IQ, Intelligence Quotient; occup class, Occupational class; HADS, Hospital Anxiety and Depression Scale.

For peer review only

For peer review only - http://bmjopen.bmj.com/site/about/guidelines.xhtml

Supplementary Table 9 Hazard ratios (95% confidence intervals) for the association between general functional health literacy and mortality, controlling for sociodemographic, cognitive ability, and health variables. Models are run on a sub-sample of participants with all variables of interest (N = 677).

	Model 1<br>Age and sex	Model 2<br>+ education	Model 3<br>+ age-11 IQ	Model 4<br>+ current fluid ability	Model 5<br>+ occup class	Model 6<br>+ health status
General health literacy	0.769 (0.640 to 0.924)**	0.736 (0.602 to 0.901)**	0.742 (0.586 to 0.939)*	0.868 (0.669 to 1.126)	0.903 (0.694 to 1.176)	0.950 (0.725 to 1.245)
Age	0.940 (0.701 to 1.260)	0.937 (0.699 to 1.256)	0.937 (0.699 to 1.257)	0.925 (0.688 to 1.243)	0.934 (0.692 to 1.260)	0.942 (0.700 to 1.266)
Sex						
Female	Reference	Reference	Reference	Reference	Reference	Reference
Male	1.264 (0.863 to 1.851)	1.256 (0.858 to 1.840)	1.255 (0.857 to 1.839)	1.328 (0.906 to 1.947)	1.205 (0.794 to 1.829)	1.337 (0.869 to 2.056)
Years of Education		1.096 (0.915 to 1.312)	1.098 (0.914 to 1.320)	1.114 (0.927 to 1.340)	1.229 (1.010 to 1.497)*	1.255 (1.030 to 1.528)*
Age-11 IQ			0.999 (0.984 to 1.014)	1.006 (0.991 to 1.022)	1.007 (0.991 to 1.023)	1.007 (0.992 to 1.023)
Fluid ability				0.692 (0.534 to 0.898)**	0.708 (0.543 to 0.922)*	0.770 (0.589 to 1.007)
Occupational class						
Professional					Reference	Reference
Managerial/technical					1.863 (0.941 to 3.689)	1.870 (0.945 to 3.700)
Skilled: non-manual					2.070 (0.976 to 4.390)	2.192 (1.035 to 4.640)*
Skilled: manual					3.072 (1.377 to 6.857)**	2.823 (1.252 to 6.365)*
Partly skilled/ unskilled manual					1.794 (0.570 to 5.649)	1.759 (0.557 to 5.561)
Self-rated health						
Very good/excellent						Reference
Good						1.152 (0.733 to 1.810)
Fair/poor						2.229 (1.229 to 4.042)**
HADS total score						0.975 (0.931 to 1.022)
Townsend disability						1.128 (1.040 to 1.225)**

*p < .05, **p < .01.

General health literacy, general measure of health literacy created by entering the REALM, S-TOFHLA and NVS into a PCA; IQ, Intelligence Quotient; occup class, Occupational class; HADS, Hospital Anxiety and Depression Scale.

For peer review only

For peer review only - http://bmjopen.bmj.com/site/about/guidelines.xhtml

STROBE 2007 (v4) Statement—Checklist of items that should be included in reports of *cohort studies*

Section/Topic	Item #	Recommendation	Reported on page #
**Title and abstract**	1	(*a*) Indicate the study's design with a commonly used term in the title or the abstract	1, 2
		(*b*) Provide in the abstract an informative and balanced summary of what was done and what was found	2
**Introduction**			
Background/rationale	2	Explain the scientific background and rationale for the investigation being reported	4-6
Objectives	3	State specific objectives, including any prespecified hypotheses	6-7
**Methods**			
Study design	4	Present key elements of study design early in the paper	6-7, 7-12
Setting	5	Describe the setting, locations, and relevant dates, including periods of recruitment, exposure, follow-up, and data collection	7-12
Participants	6	(*a*) Give the eligibility criteria, and the sources and methods of selection of participants. Describe methods of follow-up	7
		(*b*) For matched studies, give matching criteria and number of exposed and unexposed	NA
Variables	7	Clearly define all outcomes, exposures, predictors, potential confounders, and effect modifiers. Give diagnostic criteria, if applicable	7-11
Data sources/ measurement	8*	For each variable of interest, give sources of data and details of methods of assessment (measurement). Describe comparability of assessment methods if there is more than one group	7-11
Bias	9	Describe any efforts to address potential sources of bias	NA
Study size	10	Explain how the study size was arrived at	7, Figure 1, 13
Quantitative variables	11	Explain how quantitative variables were handled in the analyses. If applicable, describe which groupings were chosen and why	12
Statistical methods	12	(*a*) Describe all statistical methods, including those used to control for confounding	12
		(*b*) Describe any methods used to examine subgroups and interactions	NA
		(*c*) Explain how missing data were addressed	7, figure 1, 13
		(*d*) If applicable, explain how loss to follow-up was addressed	NA
		(*e*) Describe any sensitivity analyses	19
**Results**			

For peer review only

Participants	13*	(a) Report numbers of individuals at each stage of study—eg numbers potentially eligible, examined for eligibility, confirmed eligible, included in the study, completing follow-up, and analysed	7, Figure 1, 13
		(b) Give reasons for non-participation at each stage	7
		(c) Consider use of a flow diagram	Figure 1
Descriptive data	14*	(a) Give characteristics of study participants (eg demographic, clinical, social) and information on exposures and potential confounders	14, 15
		(b) Indicate number of participants with missing data for each variable of interest	14, 15
		(c) Summarise follow-up time (eg, average and total amount)	14
Outcome data	15*	Report numbers of outcome events or summary measures over time	14, 15
Main results	16	(*a*) Give unadjusted estimates and, if applicable, confounder-adjusted estimates and their precision (eg, 95% confidence interval). Make clear which confounders were adjusted for and why they were included	18
		(*b*) Report category boundaries when continuous variables were categorized	14
		(*c*) If relevant, consider translating estimates of relative risk into absolute risk for a meaningful time period	NA
Other analyses	17	Report other analyses done—eg analyses of subgroups and interactions, and sensitivity analyses	17, 19
**Discussion**			
Key results	18	Summarise key results with reference to study objectives	19-20
**Limitations**			
Interpretation	20	Give a cautious overall interpretation of results considering objectives, limitations, multiplicity of analyses, results from similar studies, and other relevant evidence	20-24
Generalisability	21	Discuss the generalisability (external validity) of the study results	22-23
**Other information**			
Funding	22	Give the source of funding and the role of the funders for the present study and, if applicable, for the original study on which the present article is based	25

*Give information separately for cases and controls in case-control studies and, if applicable, for exposed and unexposed groups in cohort and cross-sectional studies.

Note: An Explanation and Elaboration article discusses each checklist item and gives methodological background and published examples of transparent reporting. The STROBE checklist is best used in conjunction with this article (freely available on the Web sites of PLoS Medicine at http://www.plosmedicine.org/, Annals of Internal Medicine at http://www.annals.org/, and Epidemiology at http://www.epidem.com/). Information on the STROBE Initiative is available at www.strobe-statement.org.

For peer review only

BMJ Open

The role of cognitive ability in the association between functional health literacy and mortality in the Lothian Birth Cohort 1936: a prospective cohort study

Journal:	*BMJ Open*
Manuscript ID	bmjopen-2018-022502.R2
Article Type:	Research
Date Submitted by the Author:	17-Jul-2018
Complete List of Authors:	Fawns-Ritchie, Chloe; University of Edinburgh, Centre for Cognitive Ageing and Cognitive Epidemiology<br>Starr, John; University of Edinburgh<br>Deary, Ian; Dept of Psychology
**Primary Subject Heading**:	Epidemiology
Secondary Subject Heading:	Public health
Keywords:	EPIDEMIOLOGY, PUBLIC HEALTH, STATISTICS & RESEARCH METHODS

SCHOLARONE™
Manuscripts

For peer review only

The role of cognitive ability in the association between functional health literacy and mortality in the Lothian Birth Cohort 1936: a prospective cohort study

Chloe Fawns-Ritchie[1,2], John M Starr[1,3], and Ian J Deary[1,2*]

[1]Centre for Cognitive Ageing and Cognitive Epidemiology, University of Edinburgh, Edinburgh, UK

[2]Department of Psychology, University of Edinburgh, Edinburgh, UK

[3]Alzheimer Scotland Dementia Research Centre, University of Edinburgh, Edinburgh, UK

*Corresponding author: Ian J Deary, Centre for Cognitive Ageing and Cognitive Epidemiology, Department of Psychology, University of Edinburgh

7 George Square

Edinburgh, EH8 9JZ

Scotland, UK

Telephone: +44 131 650 3452

i.deary@ed.ac.uk

Word count: 5332

For peer review only

ABSTRACT

Objectives We investigated the role that childhood and old age cognitive ability play in the association between functional health literacy and mortality.

Design Prospective cohort study.

Setting This study used data from the Lothian Birth Cohort 1936 study, which recruited participants living in the Lothian region of Scotland when aged 70 years, most of whom had completed an intelligence test at age 11.

Participants 795 members of the Lothian Birth Cohort 1936 with scores on tests of functional health literacy and cognitive ability in childhood and older adulthood.

Primary and secondary outcome measures Participants were followed up for 8 years to determine mortality. Time to death in days was used as the primary outcome measure.

Results Using Cox regression, higher functional health literacy was associated with lower risk of mortality adjusting for age and sex, using the Shortened Test of Functional Health Literacy in Adults (HR = 0.95, 95% CI 0.92 to 0.98), the Newest Vital Sign (HR = 0.88, 95% CI 0.80 to 0.97), and a functional health literacy composite measure (HR = 0.77, 95% CI 0.65 to 0.92), but not the Rapid Estimate of Adult Literacy in Medicine (HR = 0.95, 95% CI 0.90 to 1.01). Adjusting for childhood intelligence did not change these associations. When additionally adjusting for fluid-type cognitive ability in older age associations between functional health literacy and mortality were attenuated and non-significant.

Conclusions Current fluid ability but not childhood intelligence attenuated the association between functional health literacy and mortality. Functional health literacy measures may, in part, assess fluid-type cognitive abilities and this may account for the association between functional health literacy and mortality.

For peer review only - http://bmjopen.bmj.com/site/about/guidelines.xhtml

Strengths and limitations of this study

- This study used three functional health literacy tests, which enabled us to create a composite functional health literacy measure.
- This study had comprehensive tests of cognitive ability measured in both childhood and old age which allowed us to investigate whether childhood and old age cognitive ability independently played a role in the relationship between functional health literacy and mortality.
- The health literacy measures used here only assessed functional health literacy and therefore we cannot determine whether cognitive ability would attenuate the association between health literacy and mortality if we used multi-dimensional health literacy measures.
- Larger samples and a longer follow-up time are needed to determine the role of cognitive ability in the association between functional health literacy and cause-specific mortality.

For peer review only

For peer review only - http://bmjopen.bmj.com/site/about/guidelines.xhtml

INTRODUCTION

Health literacy is "the degree to which individuals have the capacity to obtain, process and understand basic health information and services needed to make basic health decisions".[1] This ability is thought to be multifaceted and encompass the set of skills required to navigate the health-care environment.[2-4] One component of health literacy is functional health literacy—the reading, writing, and numeracy skills required to understand health information.[3, 5, 6] Tests designed to assess functional health literacy have been developed to measure health-related reading and numeracy skills, such as the commonly used Test of Functional Health Literacy in Adults.[5, 6] This test requires participants to read materials often used in the health-care setting, such as a medicine bottle, and answer questions about these materials.

Performance on functional health literacy tests have been associated with a range of health outcomes. Individuals with lower functional health literacy are more likely to require emergency care and have poorer skills in relation to correctly taking medication and interpreting written health materials.[7] Individuals with higher functional health literacy are more likely to take part in health-promoting behaviours such as eating a healthy diet, and are more likely to take part in routine cancer screening.[8, 9]

Successful completion of functional health literacy measures rely on cognitive functions, such as processing capacity and reasoning.[10, 11] One dominant theory in intelligence research is that there is a distinction between fluid ability, the ability to problem solve using novel material, which tends to decline with increasing age, and crystallised ability, which is the knowledge acquired throughout life which remains relatively stable across the lifespan.[12-16] Successful completion of tests of functional health literacy likely requires both crystallised abilities such as specific knowledge relating to health, and fluid abilities such as reasoning.[10, 11] It is therefore unsurpring that performance on tests of functional

For peer review only

health literacy and cognitive function are strongly related.[17-24] Some tests of functional health literacy have been found to correlate more strongly with measures of cognitive ability than with each other.[23, 25, 26] This overlap is so strong that some have proposed that functional health literacy should not be considered a unique construct but, instead, should be thought of as a specific component of cognitive function.[26]

Given the association between performance on tests of functional health literacy and cognitive ability tests, researchers have investigated whether the relationship between functional health literacy and health remain when also measuring cognitive ability. Whereas most evidence suggests that cognitive function explains a large proportion of the association between functional health literacy and health, the degree of attenuation varies.[25, 27, 28] A study using participants from the Lothian Birth Cohort 1936[25]—the same sample used in the current study—investigated whether cognitive ability in childhood and late adulthood attenuated the association between functional health literacy and physical health. In models without cognitive function, functional health literacy was associated with all three of the measures of physical health assessed. Addition of cognitive ability in older age significantly attenuated the association between functional health literacy with physical fitness by 43%, and number of natural teeth by 39%; however, it did not attenuate the association between functional health literacy and body mass index (BMI). Conversely, whereas childhood cognitive ability did not attenuate the association between functional health literacy and physical fitness, it attenuated the association between functional health literacy and number of teeth by 30%, and BMI by 88%. In the fully adjusted model which included childhood and late adulthood cognitive ability, as well as other early-life factors, the association between functional health literacy and physical fitness, though attenuated by 43%, remained significant,[25] suggesting that functional health literacy may play a small but unique role in physical fitness.

For peer review only - http://bmjopen.bmj.com/site/about/guidelines.xhtml

Mortality is arguably one of the most important health outcomes to examine. Both cognitive ability[29, 30] and functional health literacy[31] have been found to predict mortality. Researchers have therefore investigated the degree to which cognitive function explains the association between functional health literacy and mortality. When not controlling for cognitive function, Baker et al.[32] found that individuals with inadequate compared to adequate health literacy had a 50% higher risk of dying. When additionally adjusting for cognitive function, the risk reduced to 27%, but remained significant. A similar pattern of attenuation was found in another study.[33] Thus, cognitive function did not fully explain this relationship. These two studies, however, used brief measures of functional health literacy and cognitive function.

The present study sought to better understand the relationship between functional health literacy, cognitive ability and mortality using data from the LBC1936. We note that this is the same sample as used in Mõttus et al.[25] to investigate the association between functional health literacy, cognitive ability and physical health. In this previous study,[25] physical health was measured concurrently with fluid ability and functional health literacy. The current analysis is different from and complementary to this previous study in that we followed up the participants for 8 years to determine mortality status—obviously a most important health outcome. Studies that have examined the role that cognitive function plays in the association between functional health literacy and mortality used brief cognitive measures collected at the same time as the functional health literacy tests.[32, 33] It is not known whether early life cognitive ability and cognitive ability in older age play different roles in the association between health literacy and mortality. The current analysis utilises cognitive test scores collected in childhood, which are thought to measure the trait of lifelong intelligence, and current cognitive ability in older age, measured at approximately 73 years and contemporaneously with functional health literacy. The aim of this study was to

For peer review only - http://bmjopen.bmj.com/site/about/guidelines.xhtml

determine whether childhood cognitive ability and current cognitive ability in older adulthood play unique roles in the association between functional health literacy and mortality.

METHODS

Participants

LBC1936 is a cohort study of 1091 older adults born in 1936, most of whom reside in the Lothian area in Scotland. Most had taken part in the Scottish Mental Survey 1947, which tested the intelligence of almost all children born in 1936 and attending Scottish schools on 4th June 1947.[34] LBC1936 consists of a sample of these individuals who were subsequently followed-up, for the first time, at age 70 years (wave 1). To date, these participants have been followed-up a further three times at approximately 3 year intervals (waves 2-4). LBC1936 was designed principally to investigate healthy, non-pathological, cognitive ageing. Detailed information on this cohort is provided elsewhere.[35, 36] The present study used a sub-sample of 795 (413 male, 382 female) LBC1936 participants who completed tests of health literacy at wave 2 when participants were approximately aged 73 years. Figure 1 shows a flow chart of how the analytic sample for this current study was derived.

Ethical approval was obtained from the Scotland A Research Ethics Committee (07/MRE00/58). Written informed consent was obtained from participants. This study conformed to the principles embodied in the Declaration of Helsinki.

Measures

Mortality and survival time

For peer review only - http://bmjopen.bmj.com/site/about/guidelines.xhtml

For peer review only

The General Register Office for Scotland was used to identify deaths. Deaths through to end of March 2017 were recorded and this date is used as the censoring date for participants who survived. Survival time was measured in days from date of attending study visit at wave 2 to date of death or censoring date.

Functional health literacy

Three functional health literacy tests were administered at wave 2.

Rapid Estimate of Adult Literacy in Medicine (REALM):[37] This test measures participants' ability to read and correctly pronounce medical words. Participants are presented a piece of paper with a list of 66 medical words and are asked to read these words aloud. The words range in difficulty from easy ("fat") to difficult ("impetigo"). One point is given for each correctly pronounced word. One week test-retest (r = 0.99)[37] and internal consistency (Cronbach's alpha = 0.98)[38] have been found to be very high.

Shortened Test of Functional Health Literacy in Adults (S-TOFHLA):[5, 6]. In the numeracy section, participants are provided with cards with medical information on them and are asked four questions about this information. The reading comprehension section comprised a 36-item task which involved participants reading two health-related passages where every fifth to seventh word was missing and participants were to select the missing word from four options. Participants had 12 minutes to complete both sections. Here, the British version of the S-TOFHLA[9] was used which substitutes the Medicaid passage for a passage about UK prescription fee exemptions. This measure is a shortened version of the Test of Functional Health Literacy in Adults, which is seen as the gold standard functional health literacy test[39]. Successful completion of the S-TOFHLA requires the ability to read and comprehend written words and numbers in a health context. Internal consistency is high for

For peer review only - http://bmjopen.bmj.com/site/about/guidelines.xhtml

reading comprehension (Cronbach's alpha = 0.97)[6] and adequate for numeracy (Cronbach's alpha = 0.68).[6] The S-TOFHLA has been found to correlate strongly with the REALM (r = 0.80).[6]

Newest Vital Sign (NVS):[40] Participants were presented with a nutrition label from a container of ice cream and were asked to answer six questions about the information provided on this label. The NVS assesses both reading comprehension and numeracy skills associated with health as participants need to use the written text and numbers on the label to answer the questions.[40] The NVS correlates with the S-TOFHLA at r = 0.59[40] and shows reasonable internal consistency (Cronbach's alpha = 0.76).[40]

General health literacy: The three functional health literacy measures used here have been found to correlate moderately with each other.[25] To capture the shared variance between these tests, a general measure of functional health literacy was created by entering scores on the three tests into a principal component analysis (PCA). Two of these measures had skewed distributions (see Supplementary Figures 1-8), therefore Spearman's rank correlation was used in the PCA. Only the first component had an eigenvalue greater than 1, and the scree slope indicated a single component; therefore scores from the first unrotated principal component were used as a composite of functional health literacy (general functional health literacy). This component accounted for 59.7% of the total variance, confirming there was substantial shared variance between the three functional health literacy tests. The REALM, S-TOFHLA and NVS loaded 0.74, 0.80, and 0.77, respectively, on this component.

Cognitive ability

Childhood cognitive ability (age-11 IQ): As part of the Scottish Mental Survey 1947, almost all 11 year old children in Scotland in 1947 sat the Moray House Test No. 12 (MHT);[34] a

For peer review only - http://bmjopen.bmj.com/site/about/guidelines.xhtml

45-minute, group-administered intelligence test that included tasks of verbal reasoning and spatial ability, and had a maximum score of 76. In LBC1936, scores on the MHT were adjusted for age in days at testing and then were converted into standard IQ-type scores with a mean of 100 and a standard deviation of 15. This score will be used as a measure of prior, or crystallised, ability.

Current fluid ability: Participants completed a lengthy cognitive assessment.[35, 36] As has been done in previous LBC1936 studies,[23, 25] six tests administered at wave 2 thought to measure fluid-type cognitive abilities that tend decline across the lifespan[14-16] were entered into a PCA. The following tests from the Wechsler Adult Intelligence Scale-III[41] that assess non-verbal reasoning, visuospatial ability, working memory, and processing speed were used: Matrix Reasoning, Block Design, Letter-Number Sequencing, Symbol Search, Digit Span Backwards, and Digit Symbol-coding. Only the first component had an eigenvalue greater than 1 and the scree slope indicated one component, and therefore scores from this first principal component were used as a measure of current fluid ability. This component accounted 50.2% of the total variance. The loadings for the six tests were: Matrix Reasoning = 0.69; Block Design = 0.71; Letter-Number Sequencing = 0.71; Symbol Search = 0.75; Digit Span Backwards = 0.64; Digit Symbol-coding = 0.75.

Covariates

Sociodemographic variables included in this analysis were education and occupational social class. Years of full-time education completed, recorded at wave 1 when participants were aged 70 years, was used to measure education. At wave 1, participants were assigned to one of the following occupational social classes based on their highest occupational status prior to retirement:[42] professional, managerial and technical, skilled, partly skilled manual,

For peer review only - http://bmjopen.bmj.com/site/about/guidelines.xhtml

unskilled manual. Female participants were assigned the occupational class of their husband if this was higher than their own. Skilled was separated into skilled non-manual and skilled manual. Only 5 participants in this sample were assigned the occupational class of unskilled, therefore partly skilled manual, and unskilled manual were combined into one class, hereafter referred to as manual (N = 31).

Three measures of health status measured at wave 2 were used. Self-reported health was measured by asking participants whether they rated their general health to be excellent, very good, good, fair or poor. Only a small number of participants who were recorded dead at the censoring date reported poor (N = 3) or excellent (N = 17) health. Therefore, poor and fair were collapsed into one category (fair/poor; N = 73), as were very good and excellent (very good/excellent; N = 487). Total score on the Hospital Anxiety and Depression Scale (HADS)[43] was used as a measure of mood state. Higher scores on the HADS represent higher levels of anxiety and depression. Activities of daily living were assessed using the Townsend Disability Scale.[44] Participants were given a score of 0 (no difficulty completing this activity) to 2 (not able complete this activity) for nine activities, and thus higher scores represent more functional disability.

Patient and public involvement

LBC1936 participants were not involved in the development of any part of this study. The results will be disseminated to participants via a quarterly newsletter sent to LBC1936 participants.

For peer review only - http://bmjopen.bmj.com/site/about/guidelines.xhtml

Statistical analysis

SPSS version 21.0 was used to carry out this analysis. To determine whether those recorded as alive or dead at censoring date differ on demographic, functional health literacy, cognitive function, or health status variables, chi-square tests were conducted for categorical variables, independent t-tests were used for normally-distributed continuous variables, and Mann-Whitney U tests were used for non-normal continuous variables. Spearman's rank-order correlation was used to examine the relationship between functional health literacy and cognitive ability scores. To investigate the association between functional health literacy and time to death, Cox proportional hazard regression was used. For each of the functional health literacy measures of interest (REALM, S-TOFHLA, NVS, and the component score of general functional health literacy) six models were run. In Model 1 the functional health literacy measure of interest and age and sex were entered. Years of education was added in Model 2 as this has been found to be associated with functional health literacy. To determine whether cognitive ability in childhood attenuated the association between functional health literacy and mortality, age-11 IQ was added (Model 3). In Model 4, fluid-type cognitive ability in older age was additionally added to determine its role in the association between functional health literacy and mortality. Occupational class was additionally included in Model 5. Finally, health status variables (self-reported health, HADS, and Townsend) were included in Model 6. Methods to control for multiple testing were not used here. We were interested in the change in the effect size of the association between functional health literacy and mortality following the inclusion of various cognitive, sociodemographic and health variables. In the results section of the main text here, only the hazard ratios (HRs) and 95% confidence intervals (CIs) for the functional health literacy measures are reported. A more detailed description of the results for all variables in the models is given in the supplementary materials.

For peer review only - http://bmjopen.bmj.com/site/about/guidelines.xhtml

RESULTS

A total of 796 participants completed the functional health literacy measures at wave 2 (Figure 1). Following removal of one participant without information on date of death, 130 participants had died, and 665 participants were alive at the censoring date. Participant characteristics are reported in Table 1 and functional health literacy and cognitive ability scores are shown in Table 2. Those who died were more likely to be from a lower occupational class, were more likely to report poorer health, and reported more disability than those who survived. Participants who had died had lower scores on all the functional health literacy measures, and had lower fluid cognitive ability scores in older age. Age-11 IQ did not differ between the two groups.

Table 3 shows the rank order correlations between functional health literacy and cognitive ability measures. These have been reported elsewhere.[23, 25] The three functional health literacy measures correlated moderately with each other (r = 0.35-0.44, $p < .001$), and higher scores on the functional health literacy measures were correlated with higher age-11 IQ (r = 0.44-0.51, $p < .001$), and higher fluid ability (r = 0.38-0.55, $p < .001$). The three functional health literacy measures tended to correlate more strongly with measures of cognitive ability than with each other. The general functional health literacy measure also showed a strong positive correlation with both age-11 IQ (r = 0.61, $p < .001$) and fluid ability in older age (r = 0.63, $p < .001$). The correlations between all variables examined in this analysis are reported in Supplementary Table 1.

For peer review only - http://bmjopen.bmj.com/site/about/guidelines.xhtml

Table 1 Participant characteristics for participants alive or dead at censoring date and *p*-values to determine whether these characteristics differed by survival status

	N	Alive	Dead	*p*-value
Survival time (years), mean (SD)	795	8.19 (0.66)	5.23 (2.14)	
Age (years) at wave 2, mean (SD)	795	72.54 (0.70)	72.41 (0.72)	.068
Sex, n (%)	795			.069
Male		336 (50.5)	77 (59.2)	
Female		329 (49.5)	53 (40.8)	
Years of education, mean (SD)	795	10.80 (1.16)	10.71 (1.10)	.417
Occupational class, n (%)	780			.001
Professional		142 (21.7)	12 (9.4)	
Managerial/technical		249 (38.1)	49 (38.6)	
Skilled: non-manual		140 (21.4)	26 (20.5)	
Skilled: manual		96 (14.7)	35 (27.6)	
Manual		26 (4.0)	5 (3.9)	
Self-reported health, n (%)	795			<.001
Poor/fair		47 (7.1)	26 (19.9)	
Good		195 (29.4)	40 (30.5)	
Very good/excellent		422 (63.5)	65 (49.6)	
HADS total, mean (SD)	794	7.02 (4.37)	7.42 (4.62)	.342
Townsend disability, mean (SD)	794	0.89 (1.82)	1.60 (2.48)	.001

HADS, Hospital Anxiety and Depression Scale.

For peer review only

For peer review only - http://bmjopen.bmj.com/site/about/guidelines.xhtml

Table 2 Mean scores (SD) on measures of functional health literacy and cognitive ability by survival status, and *p*-values to determine whether these scores differ by survival status

	N	Alive	Dead	*p*-value
REALM score	794	65.08 (2.39)	64.67 (3.02)	.015
S-TOFHLA score	744	38.00 (3.85)	36.69 (5.37)	.025
NVS score	789	2.92 (1.90)	2.48 (1.92)	.011
General functional health literacy	740	0.05 (0.98)	-0.24 (1.08)	.007
Age-11 IQ	752	101.08 (14.99)	98.55 (16.33)	.091
Current fluid ability	789	0.07 (0.99)	-0.34 (1.00)	<.001

REALM, Rapid Estimate of Adult Literacy in Medicine; S-TOFHLA, Shortened Test of Functional Health Literacy in Adults; NVS, Newest Vital Sign; IQ, Intelligence Quotient.

The HRs for the association between functional health literacy and mortality are shown in Table 4. HRs for all variables entered into the models are reported in Supplementary Tables 2-5. In all models, the assumptions of proportional hazards were met. Given the high correlations between functional health literacy and cognitive ability, variance inflation factors (VIF) were calculated to check for multicollinearity. VIF values for all models were low (highest VIF = 2.15), suggesting there was no multicollinearity in these models.

REALM: The HRs for the REALM represent the risk of dying for a one point increase in the REALM (max score = 66). The REALM did not significantly predict mortality in Model 1 (HR = 0.95, 95% CI 0.90 to 1.01) adjusting for age and sex, or subsequently with the addition of education (Model 2), age-11 IQ (Model 3), fluid ability (Model 4), occupational class (Model 5), or health status (Model 6).

For peer review only - http://bmjopen.bmj.com/site/about/guidelines.xhtml

Table 3 Rank order correlations between functional health literacy and cognitive ability measures

	1	2	3	4	5	6
1 REALM	-					
2 S-TOFHLA	0.40*	-				
3 NVS	0.35*	0.44*	-			
4 General functional health literacy	0.71*	0.80*	0.78*	-		
5 Age-11 IQ	0.44*	0.48*	0.51*	0.61*	-	
6 Current fluid ability	0.38*	0.55*	0.55*	0.63*	0.57*	-

*$p < .001$.

REALM, Rapid Estimate of Adult Literacy in Medicine; S-TOFHLA, Shortened Test of Functional Health Literacy in Adults; NVS, Newest Vital Sign; IQ, Intelligence Quotient.

S-TOFHLA: The HRs for the S-TOFHLA represent the risk of mortality for a one point increase in S-TOFHLA score (max score = 40). With age and sex controlled for, a one-point increase in S-TOFHLA reduced the risk of dying by 5% (Model 1 HR = 0.95, 95% CI 0.92 to 0.98). Inclusion of education (Model 2) and age-11 IQ (Model 3) did not attenuate this association. This association was attenuated and became non-significant in Model 4 with the inclusion of fluid ability (HR = 0.97, 95% CI 0.93 to 1.01), and remained non-significant and continued to reduce in size following the addition of occupational class (Model 5) and health status (Model 6).

NVS: The HRs for NVS represent the risk of mortality for a one point increase in NVS score (max score = 6). In Model 1, in which age and sex were entered as covariates, NVS significantly predicted mortality. A one point increase in NVS score reduced the risk of dying by 12% (HR = 0.88, 95% CI 0.80 to 0.97). The addition of years of education (Model 2) did

For peer review only - http://bmjopen.bmj.com/site/about/guidelines.xhtml

not attenuate this association. Age-11 IQ was added in Model 3 and this did little to change the association between NVS and mortality. The inclusion of fluid ability in Model 4 greatly attenuated the association between NVS and mortality, and this association became non-significant (HR = 0.96, 95% CI 0.86 to 1.08). This association remained non-significant following the inclusion of occupational class (Model 5) and health status variables (Model 6).

General functional health literacy: The HRs for general functional health literacy represent the risk of mortality for a one SD increase in general functional health literacy. General functional health literacy predicted mortality in Model 1, controlling for age and sex. A one SD increase in general functional health literacy reduced the risk of mortality by 23% (HR = 0.77, 95% CI 0.65 to 0.92). Including years of education in Model 2 and age-11 IQ in Model 3 did little to change the association between general functional health literacy and mortality. Current fluid ability was included in Model 4 and this attenuated the association between general functional health literacy and mortality and this association was no longer significant (HR = 0.87, 95% CI 0.67 to 1.13). Adding occupational social class in Model 5 did little to change the association between general functional health literacy and mortality. Health status variables were added in Model 6 and the association between general functional health literacy and mortality was further attenuated and remained non-significant.

All models were re-run using only participants who had complete data on all of the variables of interest. These models are shown in Supplementary Tables 6-9. The associations between functional health literacy and mortality were similar to those reported here, except that, in Model 1 for the REALM (Supplementary Table 6), higher scores on the REALM significantly reduced the risk of mortality. This association was no longer significant in Model 2, following the inclusion of age-11 IQ.

For peer review only

For peer review only - http://bmjopen.bmj.com/site/about/guidelines.xhtml

Table 4 Hazard ratios (95% confidence intervals) for the association between four measures of functional health literacy and mortality, controlling for sociodemographic, cognitive, and health variables

	Model 1<br>Age and sex	Model 2<br>+ education	Model 3<br>+ age-11 IQ	Model 4<br>+ current fluid<br>ability in older age	Model 5<br>+ occup class	Model 6<br>+ health status
REALM	0.95 (0.90 to 1.01)	0.96 (0.90 to 1.01)	0.96 (0.90 to 1.02)	0.97 (0.91 to 1.04)	0.97 (0.90 to 1.04)	1.00 (0.92 to 1.07)
	N = 794	N = 794	N = 752	N = 746	N = 731	N = 728
S-TOFHLA	0.95 (0.92 to 0.98)**	0.95 (0.92 to 0.98)**	0.95 (0.91 to 0.98)**	0.97 (0.93 to 1.01)	0.98 (0.94 to 1.02)	1.00 (0.95 to 1.05)
	N = 744	N = 744	N = 702	N = 697	N = 682	N = 680
NVS	0.88 (0.80 to 0.97)**	0.88 (0.80 to 0.97)*	0.89 (0.80 to 0.99)*	0.96 (0.86 to 1.08)	0.97 (0.86 to 1.09)	0.96 (0.85 to 1.08)
	N = 789	N = 789	N = 746	N = 742	N = 727	N = 724
General functional	0.77 (0.65 to 0.92)**	0.75 (0.61 to 0.90)**	0.74 (0.59 to 0.93)*	0.87 (0.67 to 1.13)	0.911 (0.70 to 1.19)	0.95 (0.72 to 1.25)
health literacy	N = 740	N = 740	N = 698	N = 694	N = 679	N = 677

*p < .05, **p < .01

IQ, intelligence quotient; occup class, occupational class; REALM, Rapid Estimate of Adult Literacy in Medicine; S-TOFHLA, Shortened Test of Functional Health Literacy in Adults; NVS, Newest Vital Sign.

For peer review only - http://bmjopen.bmj.com/site/about/guidelines.xhtml

Sensitivity analyses: Participants who may have a dementia or possible pathological cognitive impairment were not removed prior to running these analyses. One participant self-reported a diagnosis of dementia at the wave 2 assessment. Five participants in this sample have mini-mental state exam scores below the often-used cutoff of 24[45] (one participant scored 20/30, one scored 22/30 and three scored 23/30), which suggests a possible cognitive impairment. To determine whether the presence of dementia or possible cognitive impairment affects the results, these analyses were re-run excluding these 6 individuals. All associations were very similar to those reported above (results not shown; available from the authors) and therefore the presence of dementia or possible cognitive impairment did not affect the main results.

DISCUSSION

This study investigated whether prior cognitive ability measured in childhood and current fluid cognitive ability measured in older adulthood played different roles in the association between functional health literacy and mortality. Three measures of functional health literacy were used; the REALM, S-TOFHLA, and NVS. These three measures were also used to create a composite measure of functional health literacy. The REALM, a test that requires only the ability to read and correctly pronounce medical words, did not predict mortality, even in minimally adjusted models (though it had a slightly stronger and significant association when only those with full data were included, as shown in supplementary analysis). When using functional health literacy tests that assessed reading comprehension and numeracy (S-TOFHLA, NVS, and general functional health literacy), functional health literacy predicted mortality in models adjusting for age, sex and education only. Individuals who had higher scores on the S-TOFHLA, NVS, and general functional health literacy had a lower risk of mortality than those with lower scores. Accounting for prior intelligence

For peer review only - http://bmjopen.bmj.com/site/about/guidelines.xhtml

measured in childhood did not change this association. The association between functional health literacy and mortality disappeared when contemporaneous fluid ability was accounted for. The attenuation was particularly large for the NVS and general functional health literacy.

Two previous studies used functional health literacy tests that measure reading comprehension and numeracy to investigate the role that cognitive function plays in the association between functional health literacy and mortality.[32, 33] These studies measured cognitive function concurrently with health literacy in middle-age or older adulthood and found that, although the size of the association between functional health literacy and mortality was reduced, functional health literacy still predicted mortality when cognitive function was controlled for.[32, 33] We investigated the role that both childhood cognitive ability, and cognitive ability in older age have on the association between functional health literacy and mortality. Here, fluid ability, but not childhood intelligence, attenuated the association between functional health literacy and mortality such that the association was no longer significant. Childhood cognitive ability, which was measured decades prior to the functional health literacy assessment, is thought to reflect the relatively stable trait of lifelong intelligence, whereas current fluid ability, which was measured when participants were approximately 73 years old, is a measure of current cognitive competence.[23] These results suggest that, whereas childhood intelligence did not play a role in the association between functional health literacy and mortality, current fluid-type cognitive ability in older adulthood accounted for a large proportion of this association.

A strength of this current study is that detailed measures of cognitive ability were used. Childhood intelligence was measured using a standardised test of intelligence which had good concurrent validity with other intelligence tests.[35] The fluid ability measure comprised many standardised neuropsychological tests. Both Baker et al.[32] and Bostock and Steptoe[33] used brief measures of cognitive function. Baker et al.[32] used specific

For peer review only - http://bmjopen.bmj.com/site/about/guidelines.xhtml

items from the mini-mental state exam, a measure designed to screen for cognitive impairment[45] which is insensitive to individual differences in healthy cognitive ageing. Bostock and Steptoe[33] used three brief cognitive tests administered in a non-standardised way in the participants' own home. These studies may not have used tests sensitive enough, or that covered a necessary range of cognitive functions, to fully account for the association between health literacy and mortality.

Another advantage of the current study is the use of three different tests of functional health literacy. All tests were used to measure functional health literacy; however, each test required the participant to carry out different health-related tasks. Whereas the REALM required the participant only to read and correctly pronounce words, the S-TOFHLA and NVS are more cognitively demanding tasks that assessed both reading comprehension and numeracy skills. Using these three measures enabled us to investigate whether different patterns of association between functional health literacy and mortality were found when using the different tests. By using three measures of functional health literacy, we were also able to create a composite measure of functional health literacy. This general measure was derived with the aim of creating a score that captures the shared variance between the three functional health literacy tests, providing a more comprehensive measure of functional health literacy.

The results of this study support the proposal by Reeve and Basalik[26] that functional health literacy may not be a unique construct; instead, it is tenable that tests of functional health literacy may in fact be largely measuring cognitive ability. First we found, as has been reported elsewhere,[23, 25] that tests of health literacy tended to correlate more strongly with tests of cognitive ability than with each other. The original paper describing the S-TOFHLA found that this test correlated with the REALM at $r = 0.80$,[6] suggesting these tests are measuring the same underlying ability. However, other studies have found moderate correlations between these tests, similar to ours.[46] Second, we found that the NVS, S-

For peer review only - http://bmjopen.bmj.com/site/about/guidelines.xhtml

TOFHLA and general functional health literacy no longer predicted mortality when accounting for fluid cognitive ability. The results of our study suggest that health literacy may not be independent of cognitive ability. This attenuation is likely to be because there is an overlap in the content of tests of fluid ability and the NVS and S-TOFHLA. The NVS and S-TOFHLA are cognitively demanding tasks that are likely to be substantially measuring fluid-type cognitive abilities, such as working memory and reasoning, that decline with increasing age.[15] Childhood cognitive ability did not attenuate the association between functional health literacy and mortality, suggesting that the NVS and S-TOFHLA are measuring current fluid-type cognitive capability in old age, and not lifelong intelligence. Current fluid ability in older age may be driving much of the association between functional health literacy and mortality largely because tests of functional health literacy are assessing mostly the same underlying abilities as measures of fluid ability.

Some researchers have questioned the validity of some of the functional health literacy tests used here. The Test of Functional Health Literacy in Adults is often reported as the gold standard functional health literacy test.[39] However, the NVS has been found to have poor concurrent validity with the Test of Functional Health Literacy in Adults.[39] In support of this, we found that the rank-order correlation between the NVS and S-TOFHLA was modest (r = 0.44). Concerns have been raised about the fact that the REALM assesses only the ability to read and pronounce words.[38] Knowing how to pronounce medical words may not be directly related to the ability to understand medical information, and therefore this may not adequately cover all the domains of functional health literacy.[38] Indeed, all the tests used here were designed to largely measure the component of health literacy known as functional health literacy. None of these measures assess other components of health literacy such as the skills required to critically analyse health information or the communicative skills needed to participate and navigate in the health-care environment.[3] Assessments of health literacy that

For peer review only - http://bmjopen.bmj.com/site/about/guidelines.xhtml

are designed to measure a much broader range of health literacy skills are available, such as the Health Literacy Questionnaire (HLQ)[47] and the European Health Literacy Survey Questionnaire (HLS-EU-Q).[48] The HLQ assesses nine dimensions of health literacy, including the ability to actively manage health and navigate the health care system.[47] Whereas the HLS-EU-Q measures self-reported skills in being able to access, understand, appraise, and apply health-related information in the health-care setting, as well as in disease prevention and health promotion.[48] Fluid cognitive ability may not play a role in the association between health literacy and mortality if we used these self-reported, broad, measures of health literacy, rather than the objective, but narrow, functional health literacy tests used here.

There are some limitations to this study. The LBC1936 participants were followed-up for the first time at age 70 years and therefore the sample used in this analysis will likely suffer from a survival bias as this sample is made up of individuals who have survived to the age of 70 years. LBC1936 participants also tended to have higher scores on the Moray House Test (age-11 IQ test) than Scottish-wide and Edinburgh-wide participants who also sat this test in 1947 as part of the Scottish Mental Survey.[36] Thus, individuals in this sample tended to be brighter than the original Scottish Mental Survey 1947 participants. This analysis only examined the association between functional health literacy and all-cause mortality. It is possible that there are different relationships between functional health literacy and cause-specific mortality, for example functional health literacy may only predict deaths linked to unhealthy lifestyles, such as cardiovascular disease. The follow-up period in this study was relatively short, and therefore only a small percentage of participants had died. Future studies should investigate mortality over a longer follow-up period and in larger samples to examine whether there are different patterns of association between functional health literacy and cause-specific mortality.

For peer review only - http://bmjopen.bmj.com/site/about/guidelines.xhtml

We investigated whether childhood cognitive ability and fluid ability in older age play independent roles in the association between functional health literacy and mortality. The results indicate that fluid-type cognitive capability may account for the association between functional health literacy and mortality, while childhood cognitive ability—an indicator of lifelong intelligence—does not. Researchers and clinicians should be aware that lower functional health literacy scores may actually reflect lower cognitive ability in older age, and that current cognitive capacity in older adulthood, but not lifelong intelligence, may be driving the association between functional health literacy and mortality. Future research examining the association between functional health literacy and mortality, and other health indicators, should also include measures of cognitive ability to be able to properly disentangle the relationship between functional health literacy and health.

For peer review only

Contributors CFR discussed and planned the study and analyses, analysed the data, interpreted the data and drafted the initial manuscript. JMS discussed and planned the study and analyses, interpreted the data and contributed to the manuscript. IJD discussed and planned the study and analyses, interpreted the data and contributed to the manuscript.

Funding This work was supported by the University of Edinburgh Centre for Cognitive Ageing and Cognitive Epidemiology, part of the cross council Lifelong Health and Wellbeing Initiative, funded by the Biotechnology and Biological Sciences Research Council (BBSRC), and Medical Research Council (MRC) (grant number MR/K026992/1). The Lothian Birth Cohort 1936 (LBC1936) is supported by Age UK (Disconnected Mind project).

Completing interests None declared.

Ethical approval Ethical approval was obtained from the Scotland A Research Ethics Committee (07/MRE00/58).

Data sharing statement Lothian Birth Cohort 1936 data can be requested from the Lothian Birth Cohort 1936 research team, following completion of a data request application. More information can be found at: http://www.lothianbirthcohort.ed.ac.uk/content/collaboration

Acknowledgements We thank the LBC1936 participants and LBC1936 research team members.

For peer review only

For peer review only - http://bmjopen.bmj.com/site/about/guidelines.xhtml

REFERENCES

1 Institute of Medicine. Health Literacy: A Prescription to End Confusion. Washinton, DC: The National Academies Press, 2004.

2 Baker DW. The meaning and the measure of health literacy. *J Gen Intern Med* 2006;21(8):878-83. doi:10.1111/j.1525-1497.2006.00540.x

3 Nutbeam D. Health literacy as a public health goal: a challenge for contemporary health education and communication strategies into the 21st century. *Health Promotion International 2000*;15(3):259-67.

4 Nutbeam D. The evolving concept of health literacy. *Soc Sci Med* 2008;67(12):2072-78. doi:10.1016/j.socscimed.2008.09.050 [published Online First: 25 October 2008].

5 Parker RM, Baker DW, Williams MV, et al. The test of functional health literacy in adults. *J Gen Intern Med* 1995;10(10):537-41.

6 Baker DW, Williams MV, Parker RM, et al. Development of a brief test to measure functional health literacy. *Patient Educ Couns* 1999;38(1):33-42.

7 Berkman ND, Sheridan SL, Donahue KE, et al. Low health literacy and health outcomes: an updated systematic review. *Ann Intern Med* 2011;155(2):97-107. doi:10.7326/0003-4819-155-2-201107190-00005 [published Online First: 19 July 2011].

8 Kobayashi LC, Wardle J, von Wagner C. Limited health literacy is a barrier to colorectal cancer screening in England: evidence from the English Longitudinal Study of Ageing. *Prev Med* 2014;61:100-105. doi:10.1016/j.ypmed.2013.11.012 [published Online First: 25 November 2013].

9 Wagner CV, Knight K, Steptoe A, et al. Functional health literacy and health-promoting behaviour in a national sample of British adults. *J Epidemiol Community Health* 2007;61(12):1086-90. doi:10.1136/jech.2006.053967.

For peer review only - http://bmjopen.bmj.com/site/about/guidelines.xhtml

10 Chin J, Morrow DG, Stine-Morrow EA, et al. The process-knowledge model of health literacy: evidence from a componential analysis of two commonly used measures. *J Health Commun* 2011;16 Suppl 3:222-41. doi:10.1080/10810730.2011.604702 [published Online First: 27 September 2011].

11 Morrow D, Chin J. Decision Making and Health Literacy among Older Adults. In: Strough TMGN, Löckenhoff C, eds. Aging and Decision Making: Empirical and Applied Perspectives. London: Academic Press, 2015:261-82.

12 Cattell RB. Theory of fluid and crystallized intelligence: A critical experiment. *J Educ Psychol* 1963;54(1):1-22. doi:10.1037/h0046743.

13 Horn JL. A basis for research on age differences in cognitive capabilities. In: McArdle JJ, Woodcock RW, eds. Human cognitive abilities in theory and practice. New York: Routledge, 1998:57-91.

14 Park DC, Reuter-Lorenz P. The adaptive brain: aging and neurocognitive scaffolding. *Annu Rev Psychol* 2009;60:173-96. doi:10.1146/annurev.psych.59.103006.093656.

15 Salthouse TA. Selective review of cognitive aging. *J Int Neuropsychol Soc* 2010;16(5):754-60. doi:10.1017/s1355617710000706.

16 Schaie KW. Intellectual development in adulthood: The Seattle longitudinal study. Cambridge: Cambridge University Press, 1996.

17 Bennett JS, Boyle PA, James BD, et al. Correlates of health and financial literacy in older adults without dementia. *BMC Geriatr* 2012;12:30. doi:10.1186/1471-2318-12-30 [published Online First: 12 June 2012].

18 Dahlke AR, Curtis LM, Federman AD, et al. The mini mental status exam as a surrogate measure of health literacy. *J Gen Intern Med* 2014;29(4):615-20. doi:10.1007/s11606-013-2712-x [published Online First: 11 Dec 2013].

For peer review only

For peer review only - http://bmjopen.bmj.com/site/about/guidelines.xhtml

19 Federman AD, Sano M, Wolf MS, et al. Health literacy and cognitive performance in older adults. *J Am Geritar Soc* 2009;57(8):1475-80. doi:10.1111/j.1532-5415.2009.02347.x [published Online First: 8 June 2009].

20 Kobayashi LC, Wardle J, Wolf MS, et al. Cognitive Function and Health Literacy Decline in a Cohort of Aging English Adults. *J Gen Intern Med* 2015;30(7):958-64. doi:10.1007/s11606-015-3206-9 [published Online First: 14 February 2015].

21 Kobayashi LC, Smith SG, O'Conor R, et al. The role of cognitive function in the relationship between age and health literacy: a cross-sectional analysis of older adults in Chicago, USA. *BMJ Open* 2015;5(4):e007222. doi:10.1136/bmjopen-2014-007222 [published Online First: 23 April 2015].

22 Levinthal BR, Morrow DG, Tu W, et al. Cognition and health literacy in patients with hypertension. *J Gen Intern Med* 2008;23(8):1172-6. doi:10.1007/s11606-008-0612-2 [published Online First:6 May 2008].

23 Murray C, Johnson W, Wolf MS, et al. The Association between Cognitive Ability across the Lifespan and Health Literacy in Old Age: The Lothian Birth Cohort 1936. *Intelligence* 2011;39(4):178-87. doi:10.1016/j.intell.2011.04.001 [published Online First: 4 May 2011].

24 Ownby RL, Acevedo A, Waldrop-Valverde D, et al. Abilities, skills and knowledge in measures of health literacy. *Patient Educ Couns* 2014;95(2):211-7. doi:10.1016/j.pec.2014.02.002 [published Online First: 16 February 2014].

25 Mõttus R, Johnson W, Murray C, et al. Towards understanding the links between health literacy and physical health. *Health Psychol* 2014;33(2):164-73. doi:10.1037/a0031439.

26 Reeve CL, Basalik D. Is health literacy an example of construct proliferation? A conceptual and empirical evaluation of its redundancy with general cognitive ability.

For peer review only - http://bmjopen.bmj.com/site/about/guidelines.xhtml

Intelligence 2014;44:93-102. doi:10.1016/j.intell.2014.03.004[published Online First: 16 April 2014].

27 O'Conor R, Wolf MS, Smith SG, et al. Health literacy, cognitive function, proper use, and adherence to inhaled asthma controller medications among older adults with asthma. *Chest* 2015;147(5):1307-15. doi:10.1378/chest.14-0914 [published Online First: 2 October 2014].

28 Serper M, Patzer RE, Curtis LM, et al. Health Literacy, Cognitive Ability, and Functional Health Status among Older Adults. *Health Serv Res* 2014;49(4):1249-67. doi:10.1111/1475-6773.12154 [published Online First: 30 Jan 2014].

29 Murray C, Pattie A, Starr JM, et al. Does Cognitive Ability Predict Mortality in the Ninth Decade? The Lothian Birth Cohort 1921. *Intelligence* 2012;40(5):490-98. doi:10.1016/j.intell.2012.05.003 [published Online First: 12 June 2012].

30 Pavlik VN, de Moraes SA, Szklo M, et al. Relation between cognitive function and mortality in middle-aged adults: the atherosclerosis risk in communities study. *Am J Epidemiol* 2003;157(4):327-34. doi:10.1093/aje/kwf209.

31 Baker DW, Wolf MS, Feinglass J, et al. Health literacy and mortality among elderly persons. *Arch Intern Med* 2007;167(14):1503-9. doi:10.1001/archinte.167.14.1503.

32 Baker DW, Wolf MS, Feinglass J, et al. Health literacy, cognitive abilities, and mortality among elderly persons. *J Gen Intern Med* 2008;23(6):723-6. doi:10.1007/s11606-008-0566-4 [published Online First: 11 March 2008].

33 Bostock S, Steptoe A. Association between low functional health literacy and mortality in older adults: longitudinal cohort study. *BMJ* 2012;344:e1602. doi:10.1136/bmj.e1602[published Online First: 15 March 2012].

For peer review only - http://bmjopen.bmj.com/site/about/guidelines.xhtml

34 Scottish Council for Research in Education. The trend of Scottish intelligence: A comparison of the 1947 and 1932 surveys of intelligence of eleven-year-old pupils. London, UK: University of London Press, 1949.

35 Deary IJ, Gow AJ, Taylor MD, et al. The Lothian Birth Cohort 1936: a study to examine influences on cognitive ageing from age 11 to age 70 and beyond. *BMC Geriatr* 2007;7:28. doi: 10.1186/1471-2318-7-28 [published Online First: 5 December 2007].

36 Deary IJ, Gow AJ, Pattie A, et al. Cohort profile: the Lothian Birth Cohorts of 1921 and 1936. *Int J Epidemiol* 2012;41(6):1576-84. doi:10.1093/ije/dyr197 [published Online First: 14 December 2011].

37 Davis TC, Long SW, Jackson RH, et al. Rapid estimate of adult literacy in medicine: a shortened screening instrument. *Fam Med* 1993;25(6):391-95.

38 Dumenci L, Matsuyama RK, Kuhn L, et al. On the Validity of the Rapid Estimate of Adult Literacy in Medicine (REALM) Scale as a Measure of Health Literacy. *Commun Methods Meas* 2013;7(2):134-43. doi:10.1080/19312458.2013.789839 [published Online First: 6 June 2013].

39 Mancuso JM. Assessment and measurement of health literacy: an integrative review of the literature. *Nurs Health Sci* 2009;11(1):77-89. doi:10.1111/j.1442-2018.2008.00408.x [published Online First: 10 February 2009].

40 Weiss BD, Mays MZ, Martz W, et al. Quick assessment of literacy in primary care: the newest vital sign. *Ann Fam Med* 2005;3(6):514-22. doi:10.1370/afm.405.

41 Wechsler D. WAIS-III UK administration and scoring manual. London, UK: Psychological Corporation, 1998.

42 General Register Office. Census 1951: Classification of Occupatioans. London, UK, 1956.

43 Zigmond AS, Snaith RP. The Hospital Anxiety and Depression Scale. *Acta Psychiatr Scand* 1983;67(6):361-70. doi:10.1111/j.1600-0447.1983.tb09716.x.

For peer review only

For peer review only - http://bmjopen.bmj.com/site/about/guidelines.xhtml

44 Townsend P. Poverty in the United Kingdom: a survey of household resources and standards of living. Harmondsworth, UK: Penguin, 1979.

45 Folstein MF, Folstein SE, McHugh PR. "Mini-mental state": a practical method for grading the cognitive state of patients for the clinician. *J Psychiatr Res* 1975;12(3):189-98. doi:10.1016/0022-3956(75)90026-6.

46 Jordan JE, Osborne RH, Buchbinder R. Critical appraisal of health literacy indices revealed variable underlying constructs, narrow content and psychometric weaknesses. *J Clin Epidemiol* 2011;64(4):366-79. doi: 10.1016/j.jclinepi.2010.04.005. [published Online First: 17 July 2010].

47 Osborne RH, Batterham RW, Elsworth GR, et al. The grounded psychometric development and initial validation of the Health Literacy Questionnaire (HLQ). *BMC Public Health* 2013;13(1):658. doi: 10.1186/1471-2458-13-658.

48 Sørensen K, Van den Broucke S, Pelikan JM, et al. Measuring health literacy in populations: illuminating the design and development process of the European Health Literacy Survey Questionnaire (HLS-EU-Q). *BMC Public Health* 2013;13(1):948. doi:10.1186/1471-2458-13-948. [published Online First: 10 October 2013].

For peer review only

Figure 1 Flow diagram of the sample used to investigate the role of cognitive ability in the association between health literacy and mortality (n = 795)

For peer review only

For peer review only - http://bmjopen.bmj.com/site/about/guidelines.xhtml

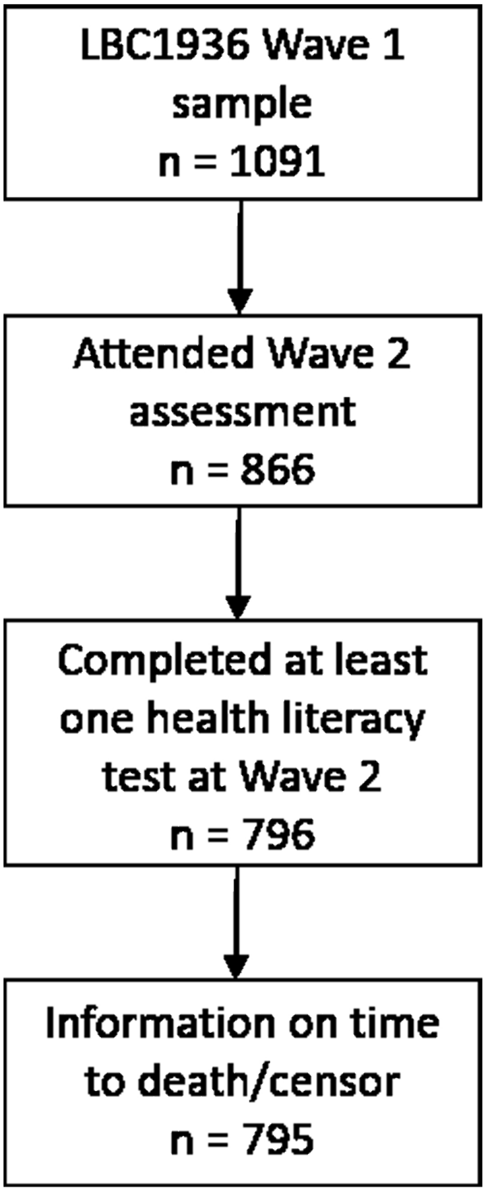

Figure 1 Flow diagram of the sample used to investigate the role of cognitive ability in the association between health literacy and mortality (n = 795)

90x225mm (300 x 300 DPI)

Supplementary material for: The role of cognitive ability in the association between functional health literacy and mortality in the Lothian Birth Cohort 1936: a prospective cohort study

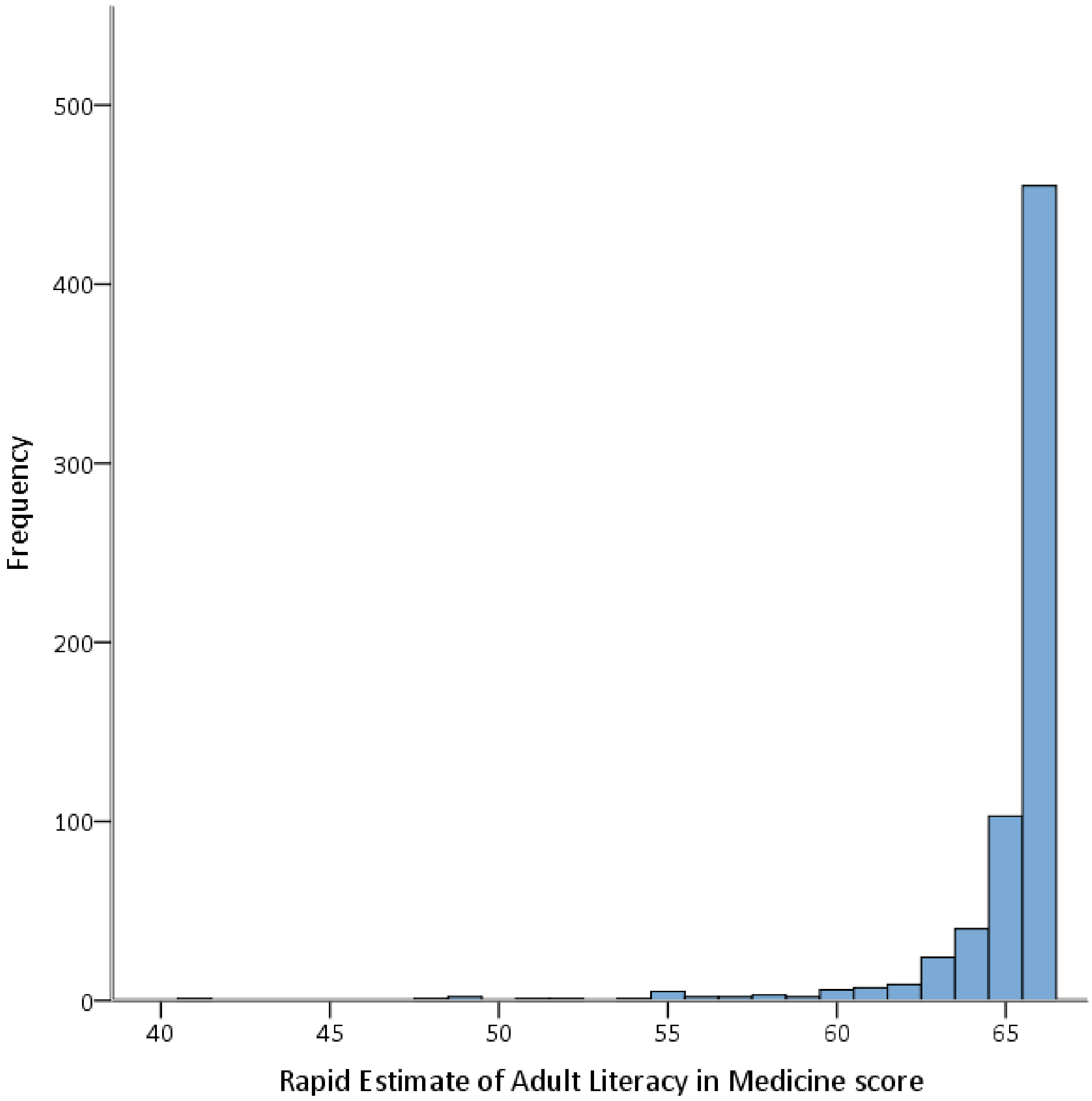

Supplementary Figure 1 Distribution of scores on the Rapid Estimate of Adult Literacy in Medicine for participants who were alive at censoring date

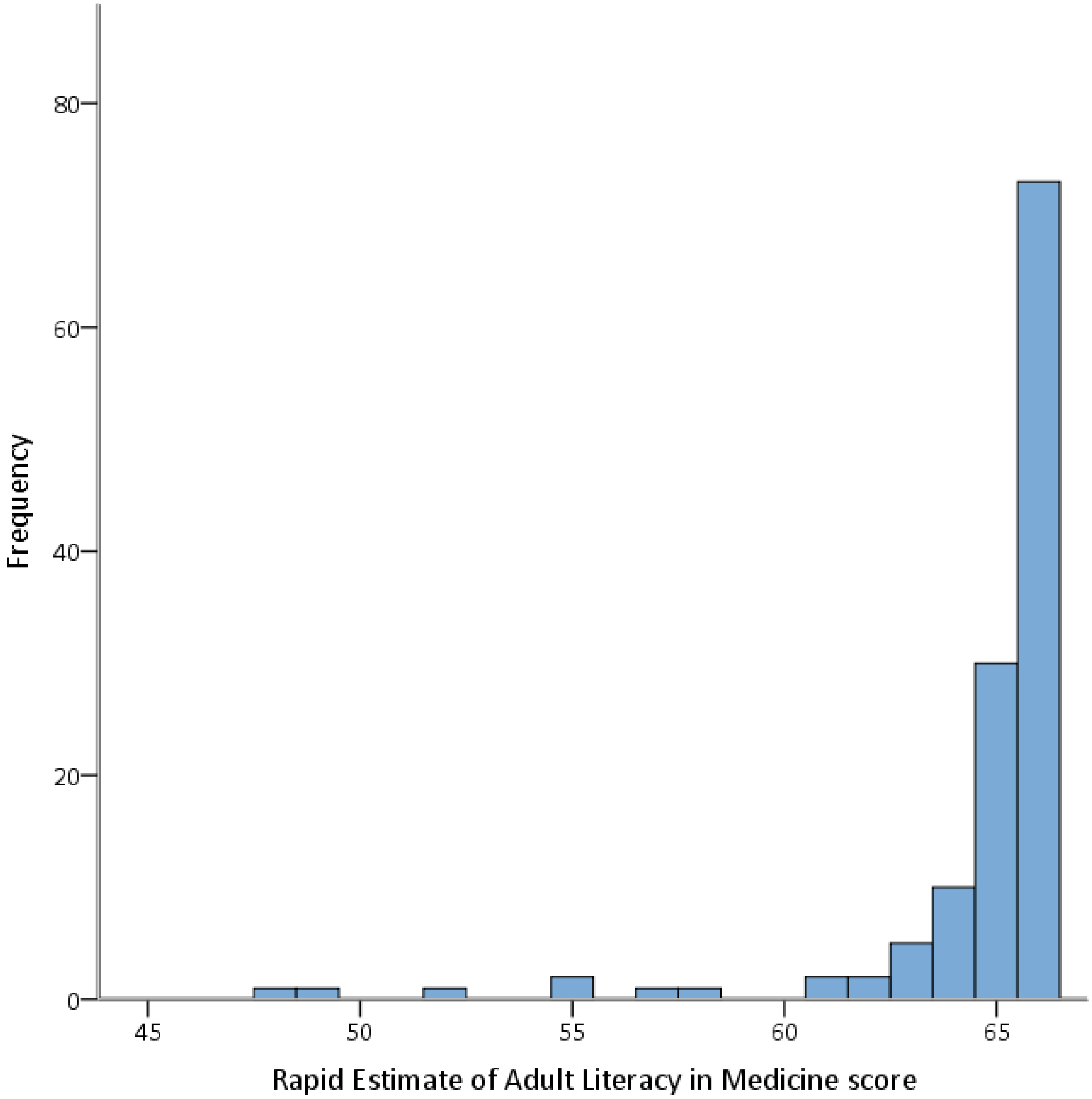

Supplementary Figure 2 Distribution of scores on the Rapid Estimate of Adult Literacy in Medicine for participants who had died by censoring date

For peer review only - http://bmjopen.bmj.com/site/about/guidelines.xhtml

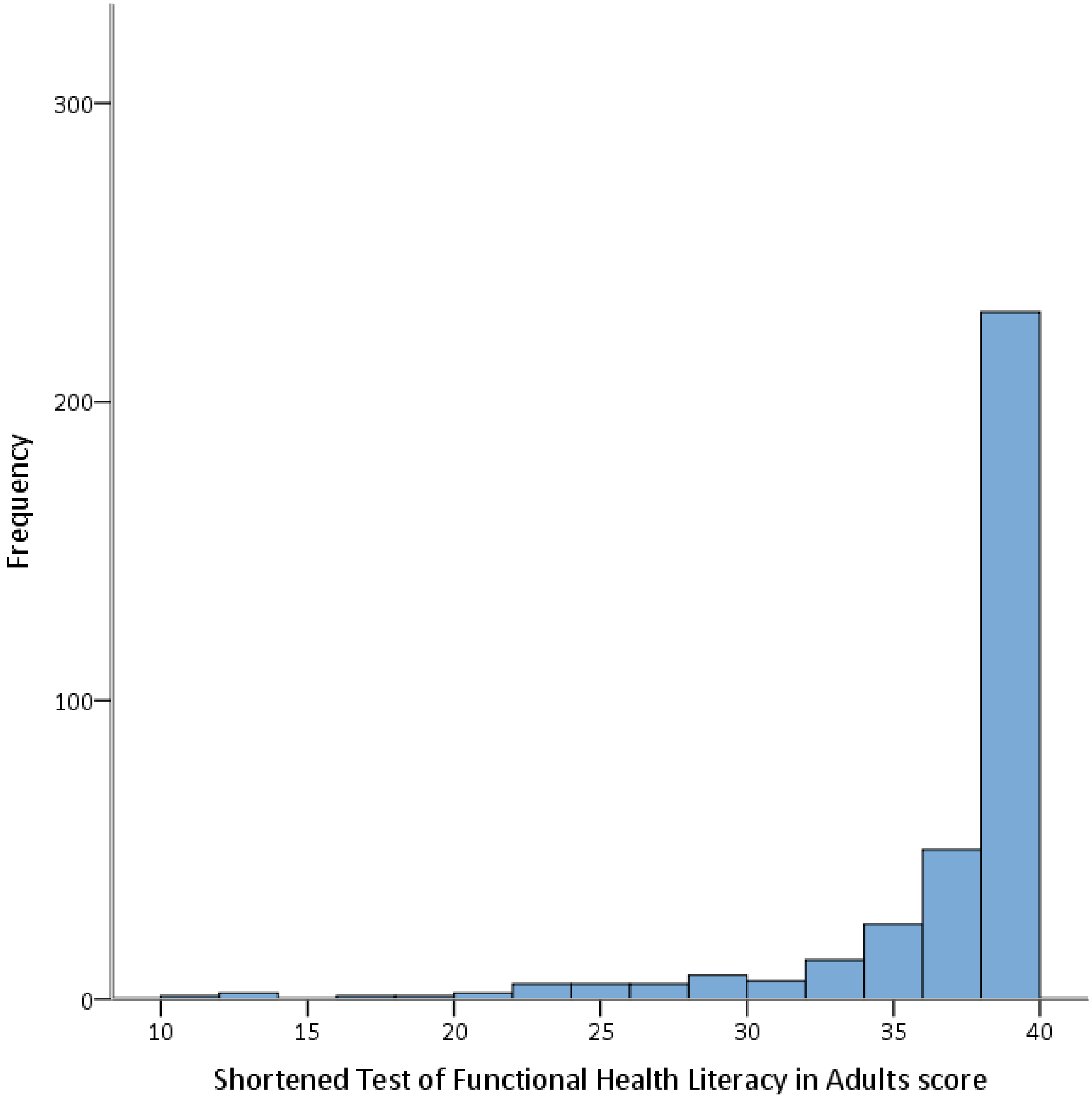

Supplementary Figure 3 Distribution of scores on the Shortened Test of Functional Health Literacy in Adults for participants who were alive at censoring date

For peer review only - http://bmjopen.bmj.com/site/about/guidelines.xhtml

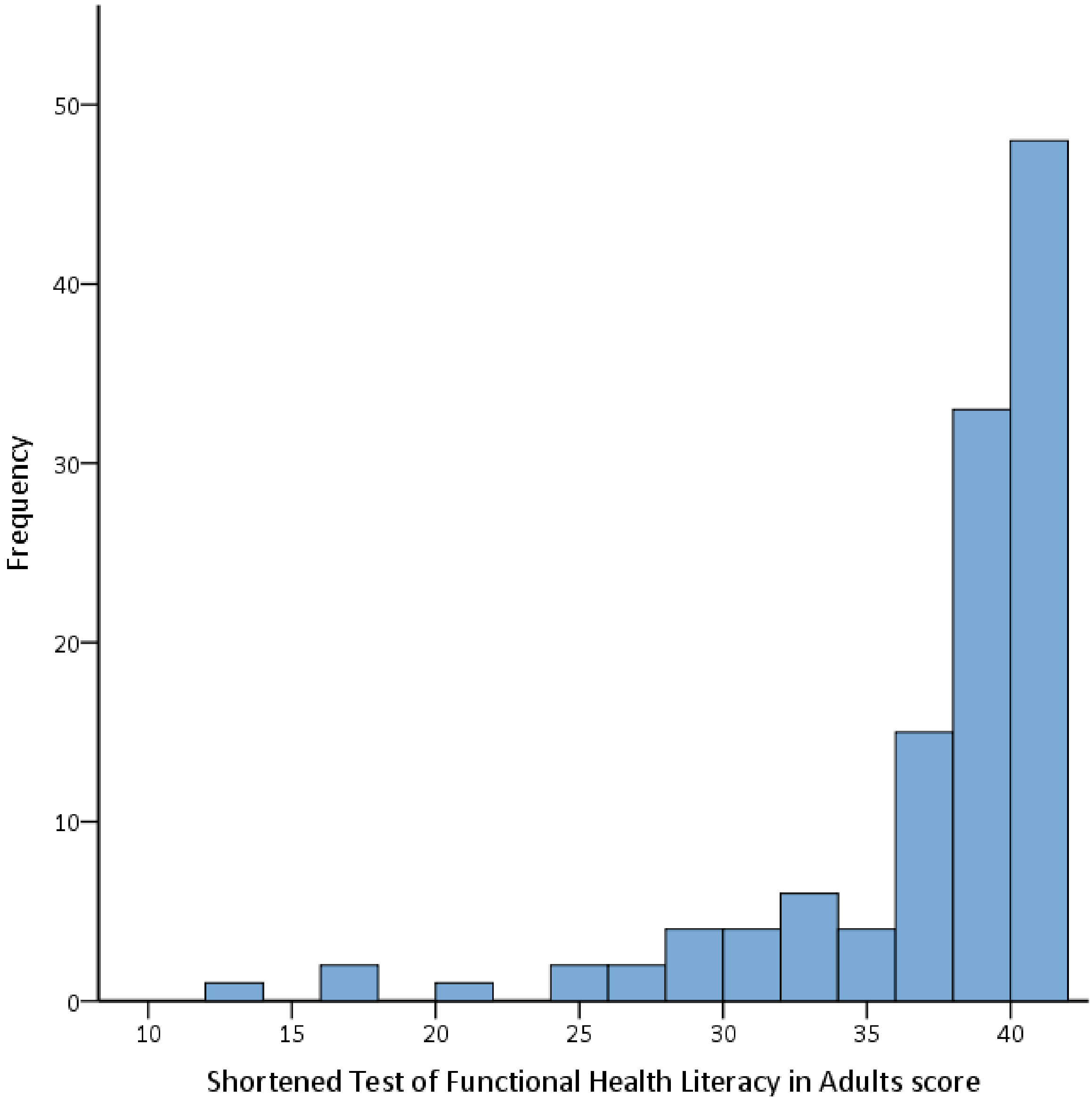

Supplementary Figure 4 Distribution of scores on the Shortened Test of Functional Health Literacy in Adults for participants who has died by censoring date

For peer review only - http://bmjopen.bmj.com/site/about/guidelines.xhtml

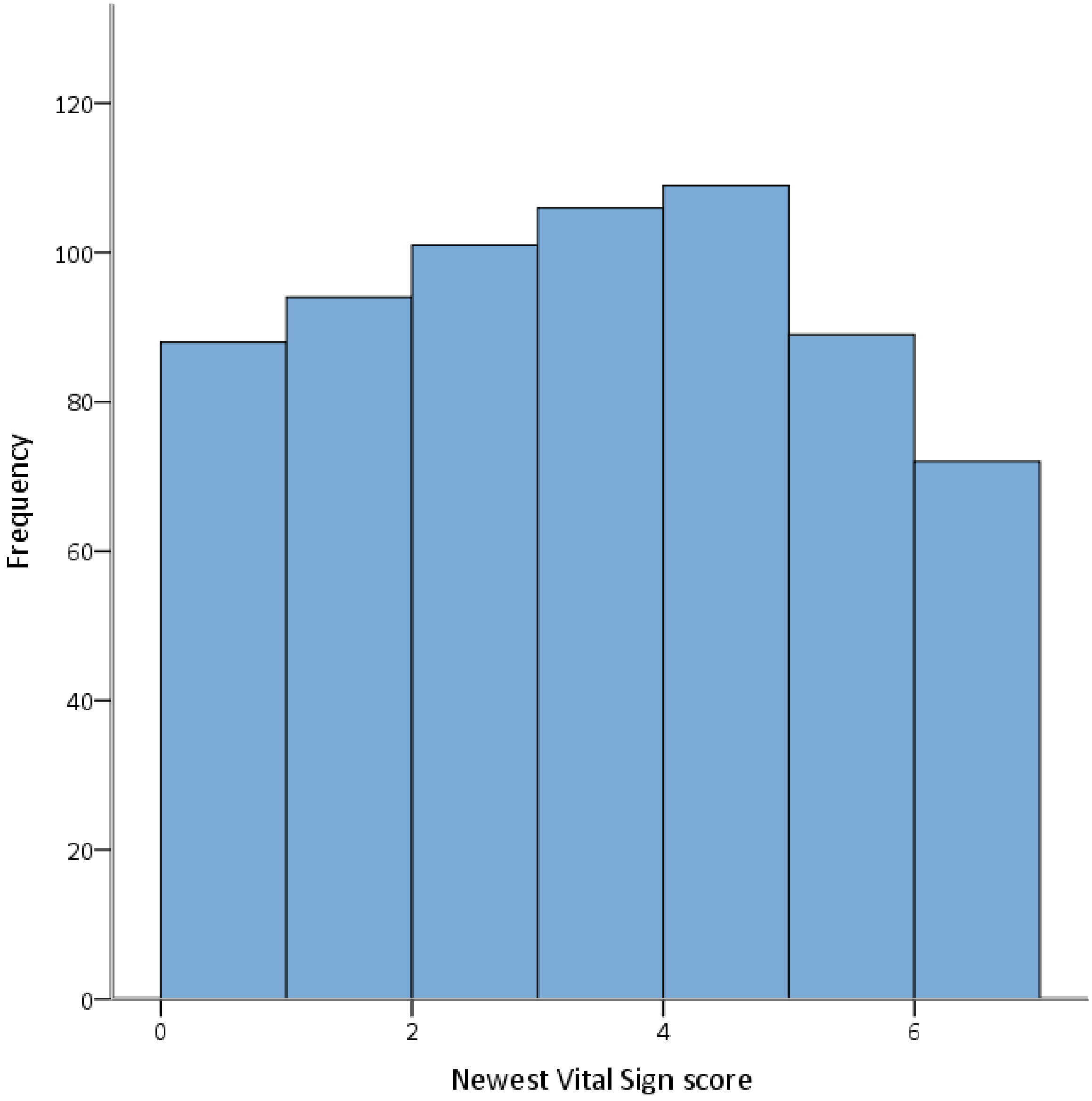

Supplementary Figure 5 Distribution of scores on the Newest Vital Sign for participants who were alive at censoring date

For peer review only - http://bmjopen.bmj.com/site/about/guidelines.xhtml

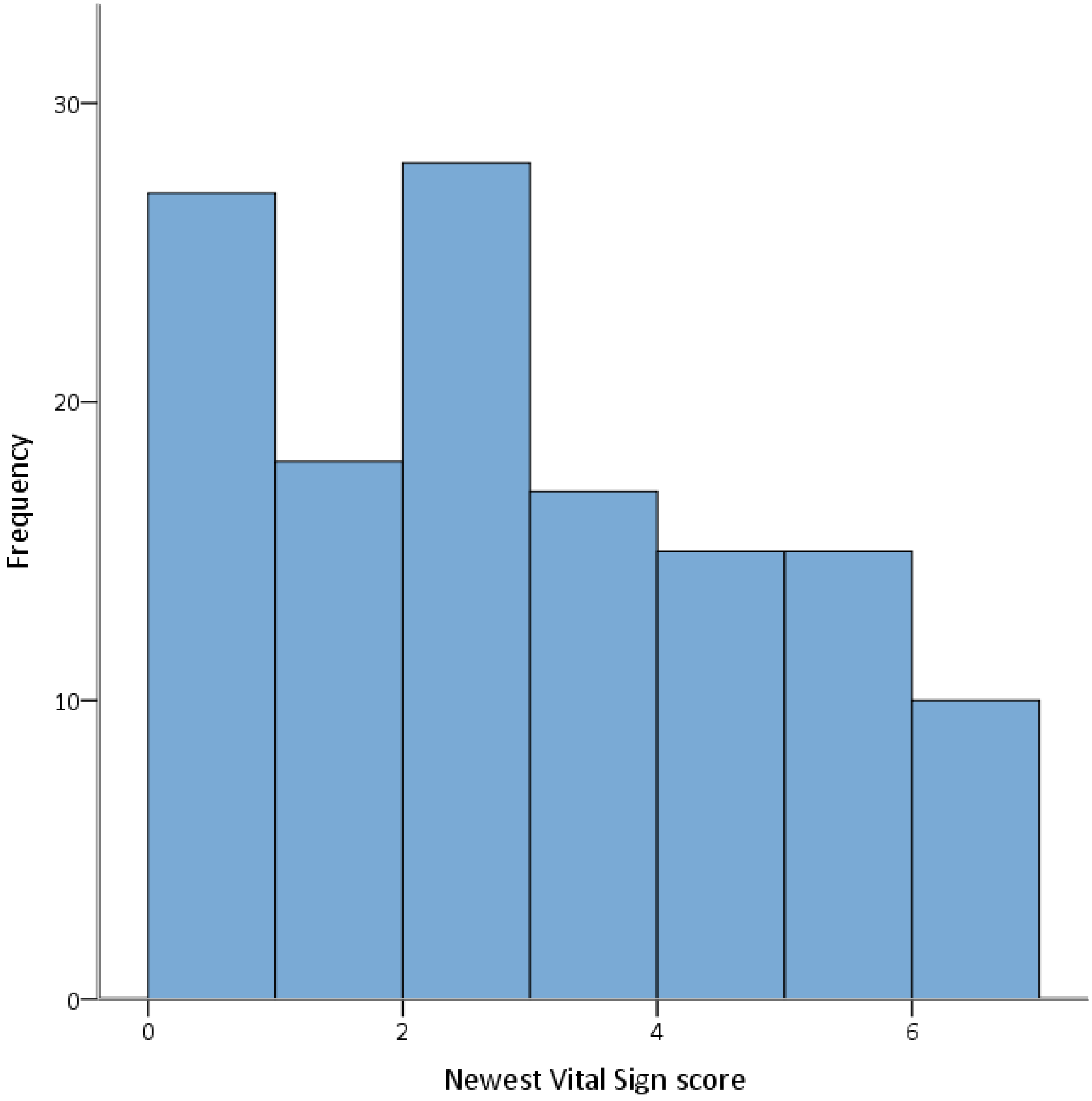

Supplementary Figure 6 Distribution of scores on the Newest Vital Sign for participants who had died by censoring date

For peer review only - http://bmjopen.bmj.com/site/about/guidelines.xhtml

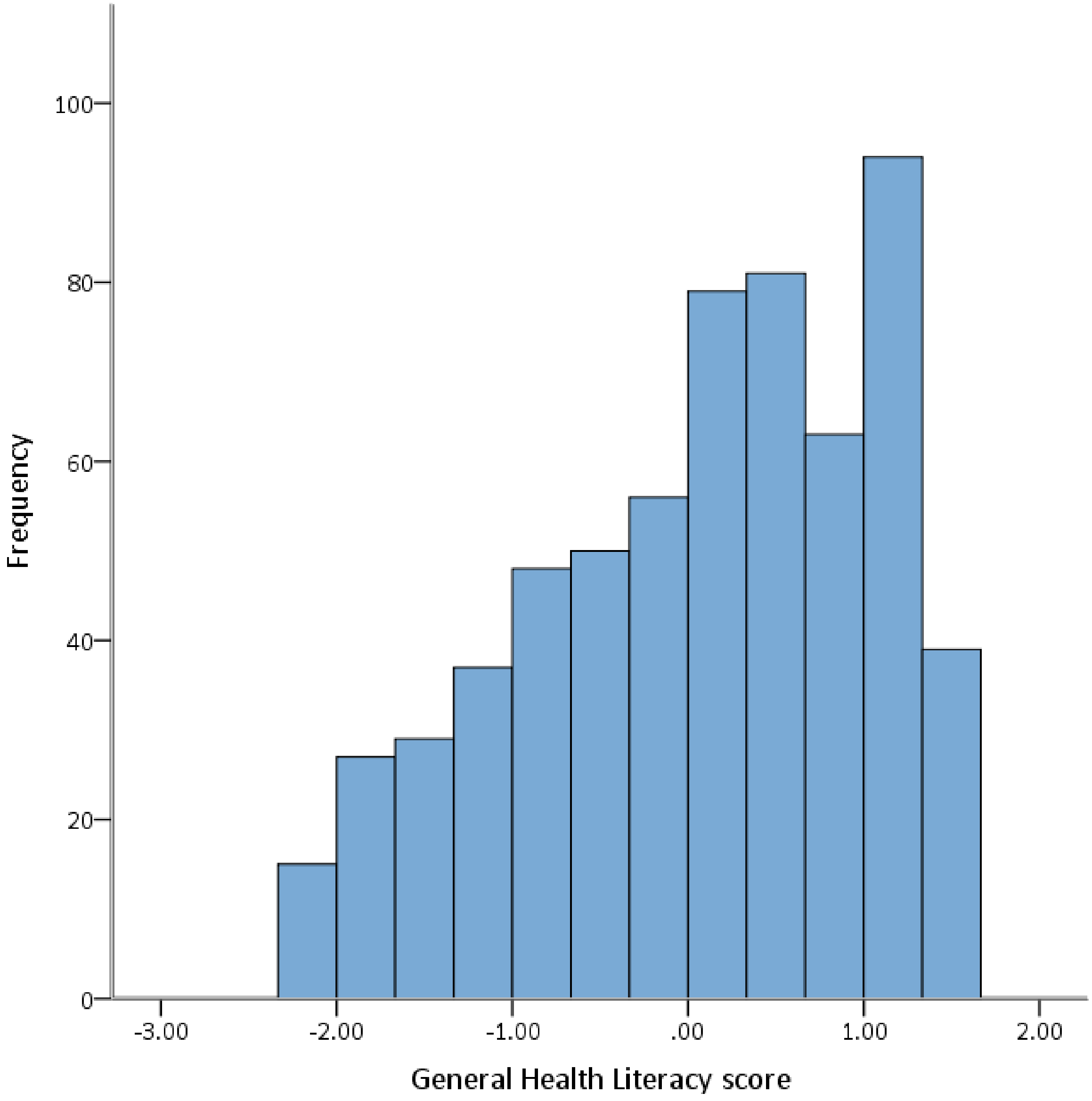

Supplementary Figure 7 Distribution of scores on General Health Literacy for participants who were alive at censoring date

only

For peer review only - http://bmjopen.bmj.com/site/about/guidelines.xhtml

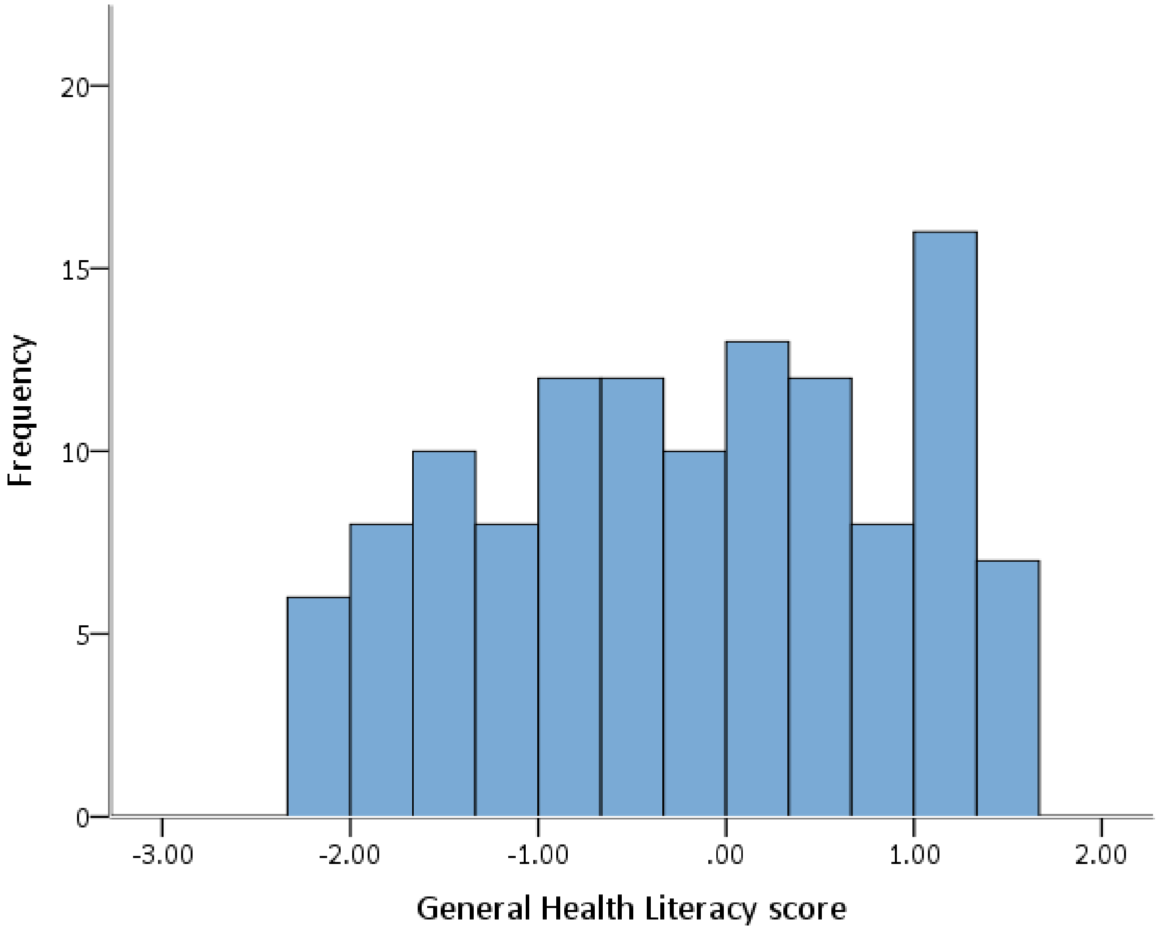

Supplementary Figure 8 Distribution of scores on General Health Literacy for participants who had died by censoring date

For peer review only - http://bmjopen.bmj.com/site/about/guidelines.xhtml

Supplementary Table 1 Rank order correlations between sociodemographic, functional health literacy, cognitive and health variables

	1	2	3	4	5	6	7	8	9	10	11	12	13
1 Age	-												
2 Sex†	0.04	-											
3 REALM	-0.12**	0.17***	-										
4 S-TOFHLA	-0.05	0.10**	0.40***	-									
5 NVS	-0.12**	0.01	0.35***	0.44***	-								
6 General functional health literacy	-0.09*	0.14***	0.71***	0.80***	0.78***	-							
7 Age-11 IQ	-0.07*	0.11**	0.44***	0.48***	0.51***	0.61***	-						
8 Fluid ability	-0.13***	0.00	0.38***	0.55***	0.55***	0.63***	0.57***	-					
9 Education	-0.05	0.03	0.31***	0.33***	0.37***	0.45***	0.45***	0.37***	-				
10 Occup class	0.05	-0.15***	-0.31***	-0.31***	-0.32***	-0.39***	-0.40***	-0.35***	-0.47***	-			
11 Self-rated health	-0.02	0.06	0.12**	0.20***	0.11**	0.18***	0.17***	0.24***	0.11**	-0.11**	-		
12 HADS	0.06	0.08*	-0.07	-0.13**	-0.11**	-0.14***	-0.13***	-0.22***	-0.08*	0.08*	-0.32***	-	
13 Townsend	0.13***	0.16***	-0.08*	-0.12**	-0.15***	-0.14***	-0.12**	-0.17***	-0.12**	0.09*	-0.35***	0.22***	-

**p* < .05, ***p* < .01, ****p* < .001.

†Correlations are point-biseral correlations. Female is coded 1 and male is coded 2.

Occupational class (ranging from 1-professional to 4-manual) and self-rated health (ranging from 1-poor/fair to 3-very good/excellent) are entered as ordinal variables.

REALM, Rapid Estimate of Adult Literacy in Medicine; S-TOFHLA, Shortened Test of Functional Health Literacy in Adults; NVS, Newest Vital Sign; IQ, Intelligence Quotient; Occup class, occupational class; HADS, Hospital Anxiety and Depression Scale; Townsend, Townsend Disability Scale.

For peer review only

DETAILED RESULTS

REALM: Hazard ratios (HRs) and 95% confidence intervals (CIs) for the associations between the REALM and mortality are shown in Supplementary Table 2. In Model 1, in which age and sex were controlled, the REALM did not significantly predict mortality (HR = 0.954, 95% CI 0.904 to 1.007), nor did age or sex. The REALM remained a non-significant predictor of mortality in Model 2, with the addition of years of education. Years of education did not predict mortality (HR = 0.963, 95% CI 0.822 to 1.128). Age-11 IQ was added in Model 3, and this did little to change the association between the REALM and mortality. Age-11 IQ did not predict mortality (HR = 0.993, 95% CI 0.980 to 1.006). The REALM remained a non-significant predictor of mortality following the inclusion of current fluid ability in Model 4. A one SD increase in fluid ability score reduced the risk of death by 37.9% (HR = 0.621, 95% CI 0.496 to 0.777). In Model 5, occupational social class was included in the model. The REALM remained non-significant. Individuals with a managerial/technical social class (HR = 2.278, 95% CI 1.161 to 4.470), a skilled non-manual social class (HR = 2.464, 95% CI 1.167 to 5.201) or a skilled manual social class (HR = 3.608, 95% CI 1.647 to 7.907) had a higher risk of death than individuals with a professional social class. Health status variables were additionally added in Model 6. The REALM remained a non-significant predictor of mortality. In this model, individuals with more years of education had a higher risk of dying (HR = 1.232, 95% CI 1.018 to 1.492). Risk of death for those who self-reported their health as fair or poor was over 2 times greater than those who reported their health to be very good or excellent (HR = 2.071, 95% CI 1.147 to 3.739). Whereas HADS score did not predict mortality, Townsend disability did. A one-point increase on the Townsend disability scale increased risk of mortality by 13.3% (HR = 1.133, 95% CI 1.044 to 1.229).

S-TOFHLA: The HRs for the association between S-TOFHLA and mortality are shown in Supplementary Table 3. In Model 1, controlling for age and sex, S-TOFHLA significantly predicted mortality. A one-point increase in S-TOFHLA reduced the risk of death by 5.2% (HR = 0.948, 95% CI 0.919 to 0.978). In this model, age and sex did not predict mortality. Adding years of education in Model 2 did not change the association between the S-TOFHLA and mortality. Years of education did

For peer review only

not predict mortality (HR = 1.020, 95% CI 0.870 to 1.197). The inclusion of age-11 IQ in Model 3 did not change the association between the S-TOFHLA and mortality. Age-11 IQ also did not predict mortality (HR = 0.997, 95% CI 0.983 to 1.011). The association between the S-TOFHLA and mortality was attenuated and became non-significant (HR = 0.967, 95% CI 0.929 to 1.007) in Model 4, additionally accounting current fluid ability. Current fluid ability significantly predicted mortality in this model. A one SD increase in fluid ability reduced the risk of death by 30.5% (HR = 0.695, 95% CI 0.545 to 0.887). Occupational class was included in Model 5, and the association between S-TOFHLA and mortality remained non-significant. Individuals with more years of education, controlling for other sociodemographic variables and cognitive function, had increased risk of death (HR = 1.219, 95% CI 1.004 to 1.481). Risk of dying was three times greater for participants with a skilled manual social class, compared to individuals with a professional social class (HR = 3.096, 95% CI 1.385 to 6.922). S-TOFHLA remained a non-significant predictor or mortality in Model 6, which included health status variables. Self-reporting health as fair or poor, compared to very good or excellent, was associated with increased risk of mortality (HR = 2.209, 95% CI 1.216 to 4.014). Higher scores on the HADS were not associated with mortality, while a higher Townsend disability score increased risk of death (HR = 1.131, 95% CI 1.039 to 1.232).

NVS: HRs for the association between NVS and mortality are shown in Supplementary Table 4. In Model 1, in which age and sex were entered as covariates, NVS significantly predicted mortality. A one point increase in NVS score reduced the risk of death by 11.8% (HR = 0.882, 95% CI 0.805 to 0.966). Age and sex did not predict mortality. Years of education was included in Model 2 and this did not change the association between the NVS and mortality. Years of education did not predict mortality (HR = 1.007, 95% CI 0.855 to 1.186). Age-11 IQ was additionally added to the model in Model 3 and this did little to change the association between NVS and mortality and this association remained significant. Age-11 IQ did not predict mortality (HR = 0.995, 95% CI 0.982 to 1.008). The inclusion of fluid ability in Model 4 greatly attenuated the association between NVS and mortality, and this became non-significant (HR = 0.963, 95% CI 0.860 to 1.078). Fluid ability was strongly associated with risk of death. A one SD increase in fluid ability score reduced risk of dying by 37.0%

For peer review only - http://bmjopen.bmj.com/site/about/guidelines.xhtml

For peer review only

(HR = 0.630, 95% CI 0.496 to 0.800). The association between NVS and mortality remained non-significant in Model 5 following inclusion of occupational class in the model. Compared to those with a professional social class, participants with managerial or technical (HR = 2.288, 95% CI 1.166 to 4.490), skilled non-manual (HR = 2.421, 95% CI 1.147 to 5.112), and skilled manual (HR = 3.631, 95% CI 1.658 to 7.951) social class had an increased risk of death. Finally, health status variables were included in Model 6. The inclusion of health status variables did little to change the association between NVS and mortality, which remained non-significant. In this model, having more years of education was associated with increased risk of mortality (HR = 1.242, 95% CI 1.023 to 1.508). Those who reported their health as fair or poor had 2.10 times (HR = 2.099, 95% CI 1.167 to 3.775) increased risk of mortality, compared to those who self-reported their health as very good or excellent. Participants with higher scores on the Townsend disability scale also had an increased risk of mortality (HR = 1.132, 95% CI 1.044 to 1.228).

General functional health literacy: HRs for the association between general functional health literacy and mortality are shown in Supplementary Table 5. General functional health literacy predicted mortality in Model 1 (HR = 0.774, 95% CI 0.650 to 0.922), while age and sex did not. A one point increase in the general functional health literacy score reduced the risk of mortality by 22.6%. Adding years of education (Model 2) did little to change the association between general functional health literacy and mortality and this association remained significant. Years of education was not associated with mortality (HR = 1.080, 95% CI 0.909 to 1.284). General functional health literacy remained a significant predictor of mortality when age-11 IQ was added in Model 3. Age-11 IQ did not predict morality (HR = 0.999, 95% CI 0.984 to 1.014). The inclusion of current fluid ability in Model 4 attenuated the association between general functional health literacy and risk of death, and this association became non-significant (HR = 0.871, 95% CI 0.674 to 1.125). Fluid ability was a significant predictor of mortality, such that a one SD increase in fluid ability reduced risk of death by 31.3% (HR = 0.687, 95% CI 0.531 to 0.887). Including occupational social class in Model 5 did little to change the association between general functional health literacy and mortality, and this association remained non-significant. In Model 5, individuals with more years of education had a greater risk of

For peer review only - http://bmjopen.bmj.com/site/about/guidelines.xhtml

death (HR = 1.240, 95% CI 1.019 to 1.508), and those with an occupational social class of skilled manual (HR = 3.134, 95% CI 1.405 to 6.991), when compared to those with a professional occupational class, had an increased risk of mortality. Finally, health status variables were added in Model 6. The association between general functional health literacy and mortality was attenuated further and remained non-significant. Reporting fair or poor health, compared to reporting very good or excellent health increased the risk of mortality (HR = 2.229, 95% CI 1.229 to 4.042). Higher Townsend disability scores were also associated with increased risk of death (HR = 1.128, 95% CI 1.040 to 1.225). In this final model, controlling for sociodemographics and health variables, as well as age-11 IQ, the association between fluid ability and mortality was attenuated and became non-significant (HR = 0.770, 95% CI 0.589 to 1.007).

For peer review only - http://bmjopen.bmj.com/site/about/guidelines.xhtml

Supplementary Table 2 Hazard ratios (95% confidence intervals) for the association between REALM and mortality, controlling for sociodemographic, cognitive ability, and health status variables

	Model 1<br>Age and sex<br>N = 794	Model 2<br>+ education<br>N = 794	Model 3<br>+ age-11 IQ<br>N = 752	Model 4<br>+ current fluid ability<br>N = 746	Model 5<br>+ occup class<br>N = 731	Model 6<br>+ health status<br>N = 728
REALM	0.954 (0.904 to 1.007)	0.957 (0.905 to 1.013)	0.962 (0.903 to 1.025)	0.971 (0.907 to 1.039)	0.970 (0.904 to 1.040)	0.996 (0.924 to 1.074)
Age	0.940 (0.725 to 1.219)	0.939 (0.724 to 1.218)	0.944 (0.725 to 1.231)	0.879 (0.669 to 1.154)	0.908 (0.686 to 1.203)	0.933 (0.704 to 1.235)
Sex						
Female	Reference	Reference	Reference	Reference	Reference	Reference
Male	1.297 (0.909 to 1.850)	1.298 (0.910 to 1.852)	1.252 (0.869 to 1.802)	1.333 (0.927 to 1.918)	1.176 (0.787 to 1.756)	1.364 (0.898 to 2.073)
Years of education		0.963 (0.822 to 1.128)	1.022 (0.862 to 1.211)	1.089 (0.916 to 1.295)	1.201 (0.995 to 1.450)	1.232 (1.018 to 1.492)*
Age-11 IQ			0.993 (0.980 to 1.006)	1.008 (0.993 to 1.023)	1.009 (0.994 to 1.024)	1.008 (0.993 to 1.024)
Fluid ability				0.621 (0.496 to 0.777)***	0.662 (0.526 to 0.834)***	0.727 (0.574 to 0.922)**
Occupational class						
Professional					Reference	Reference
Managerial/technical					2.278 (1.161 to 4.470)*	2.218 (1.127 to 4.365)*
Skilled: non-manual					2.464 (1.167 to 5.201)*	2.596 (1.232 to 5.474)*
Skilled: manual					3.608 (1.647 to 7.907)**	3.393 (1.532 to 7.516)**
Partly skilled/ unskilled manual					2.054 (0.651 to 6.473)	2.067 (0.656 to 6.510)
Self-rated health						
Very good/excellent						Reference
Good						1.153 (0.742 to 1.791)
Fair/poor						2.071 (1.147 to 3.739)*
HADS total score						0.972 (0.929 to 1.018)
Townsend disability						1.133 (1.044 to 1.229)**

* $p < .05$, **$p < .01$, ***$p < .001$.

REALM, Rapid Estimate of Adult Literacy in Medicine; IQ, Intelligence Quotient; occup class, Occupational class; HADS, Hospital Anxiety and Depression Scale.

For peer review only - http://bmjopen.bmj.com/site/about/guidelines.xhtml

For peer review only

Supplementary Table 3 Hazard ratios (95% confidence intervals) for the association between S-TOFHLA and mortality, controlling for sociodemographic, cognitive ability, and health variables

	Model 1<br>Age and sex<br>N = 744	Model 2<br>+ education<br>N = 744	Model 3<br>+ age-11 IQ<br>N = 702	Model 4<br>+ current fluid ability<br>N = 697	Model 5<br>+ occup class<br>N = 682	Model 6<br>+ health status<br>N = 680
S-TOFHLA	0.948 (0.919 to 0.978)**	0.947 (0.917 to 0.978)**	0.947 (0.913 to 0.982)**	0.967 (0.929 to 1.007)	0.976 (0.935 to 1.019)	0.998 (0.953 to 1.046)
Age	0.882 (0.665 to 1.170)	0.882 (0.665 to 1.170)	0.889 (0.666 to 1.186)	0.871 (0.652 to 1.164)	0.919 (0.682 to 1.238)	0.936 (0.697 to 1.256)
Sex						
Female	Reference	Reference	Reference	Reference	Reference	Reference
Male	1.307 (0.909 to 1.879)	1.309 (0.910 to 1.881)	1.277 (0.881 to 1.851)	1.349 (0.930 to 1.956)	1.204 (0.797 to 1.818)	1.352 (0.881 to 2.074)
Years of education		1.020 (0.870 to 1.197)	1.066 (0.896 to 1.268)	1.111 (0.932 to 1.326)	1.219 (1.004 to 1.481)*	1.249 (1.026 to 1.520)*
Age-11 IQ			0.997 (0.983 to 1.011)	1.006 (0.991 to 1.022)	1.007 (0.991 to 1.022)	1.006 (0.991 to 1.022)
Fluid ability				0.695 (0.545 to 0.887)**	0.717 (0.557 to 0.922)*	0.759 (0.587 to 0.982)*
Occupational class						
Professional					Reference	Reference
Managerial/technical					1.889 (0.956 to 3.734)	1.844 (0.931 to 3.650)
Skilled: non-manual					2.108 (0.994 to 4.470)	2.207 (1.042 to 4.673)*
Skilled: manual					3.096 (1.385 to 6.922)**	2.881 (1.275 to 6.509)*
Partly skilled/ unskilled manual					1.786 (0.566 to 5.636)	1.773 (0.562 to 5.598)
Self-rated health						
Very good/excellent						Reference
Good						1.147 (0.728 to 1.807)
Fair/poor						2.209 (1.216 to 4.014)**
HADS total score						0.974 (0.930 to 1.021)
Townsend disability						1.131 (1.039 to 1.232)**

*$p < .05$, **$p < .01$, ***$p < .001$.

S-TOFHLA, Shortened Test of Functional Health Literacy in Adults; IQ, Intelligence Quotient; occup class, Occupational class; HADS, Hospital Anxiety and Depression Scale.

For peer review only - http://bmjopen.bmj.com/site/about/guidelines.xhtml

Supplementary Table 4 Hazard ratios (95% confidence intervals) for the association between NVS and mortality, controlling for sociodemographic, cognitive ability, and health variables

	Model 1<br>Age and sex<br>N = 789	Model 2<br>+ education<br>N = 789	Model 3<br>+ age-11 IQ<br>N = 746	Model 4<br>+ current fluid ability<br>N = 742	Model 5<br>+ occup class<br>N = 727	Model 6<br>+ health status<br>N = 724
NVS	0.882 (0.805 to 0.966)**	0.880 (0.799 to 0.970)*	0.892 (0.802 to 0.992)*	0.963 (0.860 to 1.078)	0.967 (0.861 to 1.086)	0.961 (0.853 to 1.082)
Age	0.942 (0.727 to 1.221)	0.942 (0.726 to 1.221)	0.942 (0.722 to 1.228)	0.890 (0.678 to 1.168)	0.919 (0.694 to 1.217)	0.937 (0.708 to 1.242)
Sex						
Female	Reference	Reference	Reference	Reference	Reference	Reference
Male	1.343 (0.946 to 1.906)	1.343 (0.947 to 1.907)	1.279 (0.892 to 1.834)	1.346 (0.939 to 1.928)	1.180 (0.791 to 1.760)	1.355 (0.893 to 2.057)
Years of education		1.007 (0.855 to 1.186)	1.056 (0.888 to 1.257)	1.093 (0.917 to 1.302)	1.208 (0.998 to 1.463)	1.242 (1.023 to 1.508)*
Age-11 IQ			0.995 (0.982 to 1.008)	1.007 (0.993 to 1.021)	1.008 (0.993 to 1.023)	1.009 (0.994 to 1.023)
Fluid ability				0.630 (0.496 to 0.800)***	0.670 (0.524 to 0.857)**	0.748 (0.580 to 0.966)*
Occupational class						
Professional					Reference	Reference
Managerial/technical					2.288 (1.166 to 4.490)*	2.243 (1.140 to 4.414)*
Skilled: non-manual					2.421 (1.147 to 5.112)*	2.593 (1.231 to 5.463)*
Skilled: manual					3.631 (1.658 to 7.951)**	3.360 (1.522 to 7.415) **
Partly skilled/ unskilled manual					2.125 (0.677 to 6.669)	2.086 (0.661 to 6.578)
Self-rated health						
Very good/excellent						Reference
Good						1.175 (0.756 to 1.826)
Fair/poor						2.099 (1.167 to 3.775)*
HADS total score						0.973 (0.930 to 1.018)
Townsend disability						1.132 (1.044 to 1.228)**

*$p < .05$, **$p < .01$.

NVS, Newest Vital Sign; IQ, Intelligence Quotient; occup class, Occupational class; HADS, Hospital Anxiety and Depression Scale.

For peer review only - http://bmjopen.bmj.com/site/about/guidelines.xhtml

Supplementary Table 5 Hazard ratios (95% confidence intervals) for the association between general functional health literacy and mortality, controlling for sociodemographic, cognitive ability, and health variables

	Model 1<br>Age and sex<br>N = 740	Model 2<br>+ education<br>N = 740	Model 3<br>+ age-11 IQ<br>N = 698	Model 4<br>+ current fluid ability<br>N = 694	Model 5<br>+ occup class<br>N = 679	Model 6<br>+ health status<br>N = 677
General functional health literacy	0.774 (0.650 to 0.922)**	0.746 (0.615 to 0.905)**	0.738 (0.585 to 0.931)*	0.871 (0.674 to 1.125)	0.911 (0.700 to 1.186)	0.950 (0.725 to 1.245)
Age	0.897 (0.678 to 1.187)	0.893 (0.675 to 1.182)	0.902 (0.677 to 1.200)	0.885 (0.663 to 1.182)	0.933 (0.693 to 1.257)	0.942 (0.700 to 1.266)
Sex						
Female	Reference	Reference	Reference	Reference	Reference	Reference
Male	1.276 (0.886 to 1.838)	1.272 (0.883 to 1.833)	1.238 (0.852 to 1.799)	1.327 (0.912 to 1.930)	1.178 (0.778 to 1.784)	1.337 (0.869 to 2.056)
Years of education		1.080 (0.909 to 1.284)	1.119 (0.936 to 1.339)	1.134 (0.948 to 1.357)	1.240 (1.019 to 1.508)*	1.255 (1.030 to 1.528)*
Age-11 IQ			0.999 (0.984 to 1.014)	1.006 (0.991 to 1.022)	1.006 (0.991 to 1.022)	1.007 (0.992 to 1.023)
Fluid ability				0.687 (0.531 to 0.887)**	0.707 (0.543 to 0.921)*	0.770 (0.589 to 1.007)
Occupational class						
Professional					Reference	Reference
Managerial/technical					1.901 (0.962 to 3.756)	1.870 (0.945 to 3.700)
Skilled: non-manual					2.076 (0.979 to 4.401)	2.192 (1.035 to 4.640)*
Skilled: manual					3.134 (1.405 to 6.991)**	2.823 (1.252 to 6.365)*
Partly skilled/ unskilled manual					1.824 (0.580 to 5.741)	1.759 (0.557 to 5.561)
Self-rated health						
Very good/excellent						Reference
Good						1.152 (0.733 to 1.810)
Fair/poor						2.229 (1.229 to 4.042)**
HADS total score						0.975 (0.931 to 1.022)
Townsend disability						1.128 (1.040 to 1.225)**

* $p < .05$, ** $p < .01$.

General functional health literacy, general measure of functional health literacy created by entering the REALM, S-TOFHLA and NVS into a PCA; IQ, Intelligence Quotient; occup class, Occupational class; HADS, Hospital Anxiety and Depression Scale.

For peer review only - http://bmjopen.bmj.com/site/about/guidelines.xhtml

Supplementary Table 6 Hazard ratios (95% confidence intervals) for the association between REALM and mortality, controlling for sociodemographic, cognitive ability, and health status variables. Models are run on a sub-sample participants with all variables of interest (N = 728).

	Model 1<br>Age and sex	Model 2<br>+ education	Model 3<br>+ age-11 IQ	Model 4<br>+ current fluid ability	Model 5<br>+ occup class	Model 6<br>+ health status
REALM	0.944 (0.894 to 0.997)*	0.946 (0.894 to 1.001)	0.959 (0.900 to 1.021)	0.966 (0.904 to 1.033)	0.969 (0.904 to 1.039)	0.996 (0.924 to 1.074)
Age	1.002 (0.763 to 1.316)	1.001 (0.762 to 1.315)	0.999 (0.761 to 1.312)	0.931 (0.704 to 1.231)	0.930 (0.700 to 1.234)	0.933 (0.704 to 1.235)
Sex						
Female	Reference	Reference	Reference	Reference	Reference	Reference
Male	1.303 (0.897 to 1.892)	1.304 (0.898 to 1.893)	1.289 (0.887 to 1.872)	1.358 (0.935 to 1.971)	1.224 (0.815 to 1.836)	1.364 (0.898 to 2.073)
Years of education		0.981 (0.831 to 1.158)	1.010 (0.848 to 1.204)	1.077 (0.902 to 1.287)	1.203 (0.994 to 1.455)	1.232 (1.018 to 1.492)*
Age-11 IQ			0.993 (0.980 to 1.006)	1.007 (0.992 to 1.023)	1.009 (0.993 to 1.025)	1.008 (0.993 to 1.024)
Fluid ability				0.632 (0.503 to 0.794)***	0.666 (0.528 to 0.841)**	0.727 (0.574 to 0.922)**
Occupational class						
Professional					Reference	Reference
Managerial/technical					2.201 (1.118 to 4.333)*	2.218 (1.127 to 4.365)*
Skilled: non-manual					2.482 (1.175 to 5.245)*	2.596 (1.232 to 5.474)*
Skilled: manual					3.570 (1.627 to 7.837)**	3.393 (1.532 to 7.516)**
Partly skilled/ unskilled manual					2.023 (0.641 to 6.388)	2.067 (0.656 to 6.510)
Self-rated health						
Very good/excellent						Reference
Good						1.153 (0.742 to 1.791)
Fair/poor						2.071 (1.147 to 3.739)*
HADS total score						0.972 (0.929 to 1.018)
Townsend disability						1.133 (1.044 to 1.229)**

**p* < .05, ***p* < .01, ****p* < .001.

REALM, Rapid Estimate of Adult Literacy in Medicine; IQ, Intelligence Quotient; occup class, Occupational class; HADS, Hospital Anxiety and Depression Scale.

For peer review only - http://bmjopen.bmj.com/site/about/guidelines.xhtml

Supplementary Table 7 Hazard ratios (95% confidence intervals) for the association between S-TOFHLA and mortality, controlling for sociodemographic, cognitive ability, and health variables. Models are run on a subsample of participants with all variables of interest (N = 680).

	Model 1<br>Age and sex	Model 2<br>+ education	Model 3<br>+ age-11 IQ	Model 4<br>+ current fluid ability	Model 5<br>+ occup class	Model 6<br>+ health status
S-TOFHLA	0.947 (0.917 to 0.978)**	0.945 (0.914 to 0.977)**	0.949 (0.913 to 0.985)**	0.969 (0.930 to 1.010)	0.975 (0.934 to 1.018)	0.998 (0.953 to 1.046)
Age	0.924 (0.688 to 1.242)	0.925 (0.688 to 1.242)	0.927 (0.690 to 1.245)	0.911 (0.677 to 1.224)	0.919 (0.681 to 1.240)	0.936 (0.697 to 1.256)
Sex						
Female	Reference	Reference	Reference	Reference	Reference	Reference
Male	1.304 (0.893 to 1.902)	1.306 (0.895 to 1.905)	1.298 (0.889 to 1.896)	1.356 (0.928 to 1.981)	1.233 (0.814 to 1.866)	1.352 (0.881 to 2.074)
Years of education		1.033 (0.874 to 1.222)	1.046 (0.875 to 1.250)	1.092 (0.911 to 1.309)	1.208 (0.994 to 1.469)	1.249 (1.026 to 1.520)*
Age-11 IQ			0.997 (0.983 to 1.011)	1.006 (0.991 to 1.022)	1.007 (0.992 to 1.023)	1.006 (0.991 to 1.022)
Fluid ability				0.699 (0.545 to 0.895)**	0.717 (0.556 to 0.923)*	0.759 (0.587 to 0.982)*
Occupational class						
Professional					Reference	Reference
Managerial/technical					1.853 (0.935 to 3.670)	1.844 (0.931 to 3.650)
Skilled: non-manual					2.105 (0.992 to 4.464)	2.207 (1.042 to 4.673)*
Skilled: manual					3.038 (1.358 to 6.796)**	2.881 (1.275 to 6.509)*
Partly skilled/<br>unskilled manual					1.755 (0.556 to 5.541)	1.773 (0.562 to 5.598)
Self-rated health						
Very good/excellent						Reference
Good						1.147 (0.728 to 1.807)
Fair/poor						2.209 (1.216 to 4.014)**
HADS total score						0.974 (0.930 to 1.021)
Townsend disability						1.131 (1.039 to 1.232)**

*$p < .05$, **$p < .01$.

S-TOFHLA, Shortened Test of Functional Health Literacy in Adults; IQ, Intelligence Quotient; occup class, Occupational class; HADS, Hospital Anxiety and Depression Scale.

For peer review only

Supplementary Table 8 Hazard ratios (95% confidence intervals) for the association between NVS and mortality, controlling for sociodemographic, cognitive ability, and health variables. Models are run on a sub-sample of participants with all variables of interest (N = 724).

	Model 1<br>Age and sex	Model 2<br>+ education	Model 3<br>+ age-11 IQ	Model 4<br>+ current fluid ability	Model 5<br>+ occup class	Model 6<br>+ health status
NVS	0.880 (0.800 to 0.968)**	0.875 (0.790 to 0.968)*	0.887 (0.796 to 0.989)*	0.953 (0.850 to 1.070)	0.960 (0.854 to 1.079)	0.961 (0.853 to 1.082)
Age	0.993 (0.756 to 1.306)	0.993 (0.756 to 1.306)	0.992 (0.754 to 1.304)	0.944 (0.714 to 1.248)	0.940 (0.709 to 1.248)	0.937 (0.708 to 1.242)
Sex						
Female	Reference	Reference	Reference	Reference	Reference	Reference
Male	1.346 (0.933 to 1.943)	1.346 (0.933 to 1.943)	1.326 (0.916 to 1.919)	1.373 (0.950 to 1.986)	1.228 (0.820 to 1.840)	1.355 (0.893 to 2.057)
Years of education		1.029 (0.866 to 1.222)	1.048 (0.876 to 1.253)	1.084 (0.905 to 1.298)	1.212 (0.999 to 1.470)	1.242 (1.023 to 1.508)*
Age-11 IQ			0.995 (0.982 to 1.008)	1.006 (0.992 to 1.021)	1.008 (0.993 to 1.023)	1.009 (0.994 to 1.023)
Fluid ability				0.645 (0.506 to 0.822)***	0.678 (0.529 to 0.869)**	0.748 (0.580 to 0.966)*
Occupational class						
Professional					Reference	Reference
Managerial/technical					2.211 (1.123 to 4.354)*	2.243 (1.140 to 4.414)*
Skilled: non-manual					2.435 (1.152 to 5.146)*	2.593 (1.231 to 5.463)*
Skilled: manual					3.590 (1.637 to 7.874)**	3.360 (1.522 to 7.415) **
Partly skilled/ unskilled manual					2.101 (0.668 to 6.604)	2.086 (0.661 to 6.578)
Self-rated health						
Very good/excellent						Reference
Good						1.175 (0.756 to 1.826)
Fair/poor						2.099 (1.167 to 3.775)*
HADS total score						0.973 (0.930 to 1.018)
Townsend disability						1.132 (1.044 to 1.228)**

**p* < .05, ***p* < .01.

NVS, Newest Vital Sign; IQ, Intelligence Quotient; occup class, Occupational class; HADS, Hospital Anxiety and Depression Scale.

For peer review only

Supplementary Table 9 Hazard ratios (95% confidence intervals) for the association between general functional health literacy and mortality, controlling for sociodemographic, cognitive ability, and health variables. Models are run on a sub-sample of participants with all variables of interest (N = 677).

	Model 1<br>Age and sex	Model 2<br>+ education	Model 3<br>+ age-11 IQ	Model 4<br>+ current fluid ability	Model 5<br>+ occup class	Model 6<br>+ health status
General health literacy	0.769 (0.640 to 0.924)**	0.736 (0.602 to 0.901)**	0.742 (0.586 to 0.939)*	0.868 (0.669 to 1.126)	0.903 (0.694 to 1.176)	0.950 (0.725 to 1.245)
Age	0.940 (0.701 to 1.260)	0.937 (0.699 to 1.256)	0.937 (0.699 to 1.257)	0.925 (0.688 to 1.243)	0.934 (0.692 to 1.260)	0.942 (0.700 to 1.266)
Sex						
Female	Reference	Reference	Reference	Reference	Reference	Reference
Male	1.264 (0.863 to 1.851)	1.256 (0.858 to 1.840)	1.255 (0.857 to 1.839)	1.328 (0.906 to 1.947)	1.205 (0.794 to 1.829)	1.337 (0.869 to 2.056)
Years of Education		1.096 (0.915 to 1.312)	1.098 (0.914 to 1.320)	1.114 (0.927 to 1.340)	1.229 (1.010 to 1.497)*	1.255 (1.030 to 1.528)*
Age-11 IQ			0.999 (0.984 to 1.014)	1.006 (0.991 to 1.022)	1.007 (0.991 to 1.023)	1.007 (0.992 to 1.023)
Fluid ability				0.692 (0.534 to 0.898)**	0.708 (0.543 to 0.922)*	0.770 (0.589 to 1.007)
Occupational class						
Professional					Reference	Reference
Managerial/technical					1.863 (0.941 to 3.689)	1.870 (0.945 to 3.700)
Skilled: non-manual					2.070 (0.976 to 4.390)	2.192 (1.035 to 4.640)*
Skilled: manual					3.072 (1.377 to 6.857)**	2.823 (1.252 to 6.365)*
Partly skilled/ unskilled manual					1.794 (0.570 to 5.649)	1.759 (0.557 to 5.561)
Self-rated health						
Very good/excellent						Reference
Good						1.152 (0.733 to 1.810)
Fair/poor						2.229 (1.229 to 4.042)**
HADS total score						0.975 (0.931 to 1.022)
Townsend disability						1.128 (1.040 to 1.225)**

*$p < .05$, **$p < .01$.

General health literacy, general measure of health literacy created by entering the REALM, S-TOFHLA and NVS into a PCA; IQ, Intelligence Quotient; occup class, Occupational class; HADS, Hospital Anxiety and Depression Scale.

For peer review only - http://bmjopen.bmj.com/site/about/guidelines.xhtml

STROBE 2007 (v4) Statement—Checklist of items that should be included in reports of *cohort studies*

Section/Topic	Item #	Recommendation	Reported on page #
**Title and abstract**	1	(*a*) Indicate the study's design with a commonly used term in the title or the abstract	1, 2
		(*b*) Provide in the abstract an informative and balanced summary of what was done and what was found	2
**Introduction**			
Background/rationale	2	Explain the scientific background and rationale for the investigation being reported	4-6
Objectives	3	State specific objectives, including any prespecified hypotheses	6-7
**Methods**			
Study design	4	Present key elements of study design early in the paper	6-7, 7-12
Setting	5	Describe the setting, locations, and relevant dates, including periods of recruitment, exposure, follow-up, and data collection	7-12
Participants	6	(*a*) Give the eligibility criteria, and the sources and methods of selection of participants. Describe methods of follow-up	7
		(*b*) For matched studies, give matching criteria and number of exposed and unexposed	NA
Variables	7	Clearly define all outcomes, exposures, predictors, potential confounders, and effect modifiers. Give diagnostic criteria, if applicable	7-11
Data sources/ measurement	8*	For each variable of interest, give sources of data and details of methods of assessment (measurement). Describe comparability of assessment methods if there is more than one group	7-11
Bias	9	Describe any efforts to address potential sources of bias	NA
Study size	10	Explain how the study size was arrived at	7, Figure 1, 13
Quantitative variables	11	Explain how quantitative variables were handled in the analyses. If applicable, describe which groupings were chosen and why	12
Statistical methods	12	(*a*) Describe all statistical methods, including those used to control for confounding	12
		(*b*) Describe any methods used to examine subgroups and interactions	NA
		(*c*) Explain how missing data were addressed	7, figure 1, 13
		(*d*) If applicable, explain how loss to follow-up was addressed	NA
		(*e*) Describe any sensitivity analyses	19
**Results**			

For peer review only

Participants	13*	(a) Report numbers of individuals at each stage of study—eg numbers potentially eligible, examined for eligibility, confirmed eligible, included in the study, completing follow-up, and analysed	7, Figure 1, 13
		(b) Give reasons for non-participation at each stage	7
		(c) Consider use of a flow diagram	Figure 1
Descriptive data	14*	(a) Give characteristics of study participants (eg demographic, clinical, social) and information on exposures and potential confounders	14, 15
		(b) Indicate number of participants with missing data for each variable of interest	14, 15
		(c) Summarise follow-up time (eg, average and total amount)	14
Outcome data	15*	Report numbers of outcome events or summary measures over time	14, 15
Main results	16	(*a*) Give unadjusted estimates and, if applicable, confounder-adjusted estimates and their precision (eg, 95% confidence interval). Make clear which confounders were adjusted for and why they were included	18
		(*b*) Report category boundaries when continuous variables were categorized	14
		(*c*) If relevant, consider translating estimates of relative risk into absolute risk for a meaningful time period	NA
Other analyses	17	Report other analyses done—eg analyses of subgroups and interactions, and sensitivity analyses	17, 19
**Discussion**			
Key results	18	Summarise key results with reference to study objectives	19-20
**Limitations**			
Interpretation	20	Give a cautious overall interpretation of results considering objectives, limitations, multiplicity of analyses, results from similar studies, and other relevant evidence	20-24
Generalisability	21	Discuss the generalisability (external validity) of the study results	22-23
**Other information**			
Funding	22	Give the source of funding and the role of the funders for the present study and, if applicable, for the original study on which the present article is based	25

*Give information separately for cases and controls in case-control studies and, if applicable, for exposed and unexposed groups in cohort and cross-sectional studies.

Note: An Explanation and Elaboration article discusses each checklist item and gives methodological background and published examples of transparent reporting. The STROBE checklist is best used in conjunction with this article (freely available on the Web sites of PLoS Medicine at http://www.plosmedicine.org/, Annals of Internal Medicine at http://www.annals.org/, and Epidemiology at http://www.epidem.com/). Information on the STROBE Initiative is available at www.strobe-statement.org.

For peer review only
